# Supplementary material for: Regression plane concept for analysing continuous cellular processes with machine learning
Source: Nat Commun. 2021 May 5;12:2532. doi: 10.1038/s41467-021-22866-x (PMC8100172; doi:10.1038/s41467-021-22866-x)
Supplement: Supplementary file 1 — Supplementary Information [file 41467_2021_22866_MOESM1_ESM.pdf]

# Supplementary Information

## Regression plane concept for analysing continuous cellular processes with machine learning

Abel Szkalisity[1,2], Filippo Piccinini[3], Attila Beleon[1], Tamas Balassa[1], Istvan Gergely Varga[4], Ede Migh[1], Csaba Molnar[1], Lassi Paavolainen[5], Sanna Timonen[5], Indranil Banerjee[6], Elina Ikonen[2], Yohei Yamauchi[7], Istvan Ando[4], Jaakko Peltonen[8,9], Vilja Pietiäinen[5], Viktor Honti[4], and Peter Horvath[1,5,10]

[1]Synthetic and Systems Biology Unit, Biological Research Centre (BRC)
[2]Department of Anatomy and Stem Cells and Metabolism Research Program, Faculty of Medicine, University of Helsinki
[3]Istituto Scientifico Romagnolo per lo Studio e la Cura dei Tumori (IRST) IRCCS
[4]Institute of Genetics, Biological Research Center (BRC)
[5]Institute for Molecular Medicine Finland-FIMM, Helsinki Institute of Life Science-HiLIFE, University of Helsinki
[6]Indian Institute of Science Education and Research (IISER)
[7]School of Cellular and Molecular Medicine, University of Bristol
[8]Faculty of Information Technology and Communication Sciences, Tampere University
[9]Department of Computer Science, Aalto University
[10]Single-Cell Technologies Ltd.

# Contents


**Supplementary Figures** **3**

Supplementary Figure 1 . . . . . . . . . . . . . . . . . . . . . . . . . 3

Supplementary Figure 2 . . . . . . . . . . . . . . . . . . . . . . . . . 5

Supplementary Figure 3 . . . . . . . . . . . . . . . . . . . . . . . . . 6

Supplementary Figure 4 . . . . . . . . . . . . . . . . . . . . . . . . . 8

Supplementary Figure 5 . . . . . . . . . . . . . . . . . . . . . . . . . 10

**Supplementary Notes** **11**

Supplementary Note 1 . . . . . . . . . . . . . . . . . . . . . . . . . . 11

Supplementary Note 2 . . . . . . . . . . . . . . . . . . . . . . . . . . 12

Supplementary Note 3 . . . . . . . . . . . . . . . . . . . . . . . . . . 43

Supplementary Note 4 . . . . . . . . . . . . . . . . . . . . . . . . . . 45

# Supplementary Figures

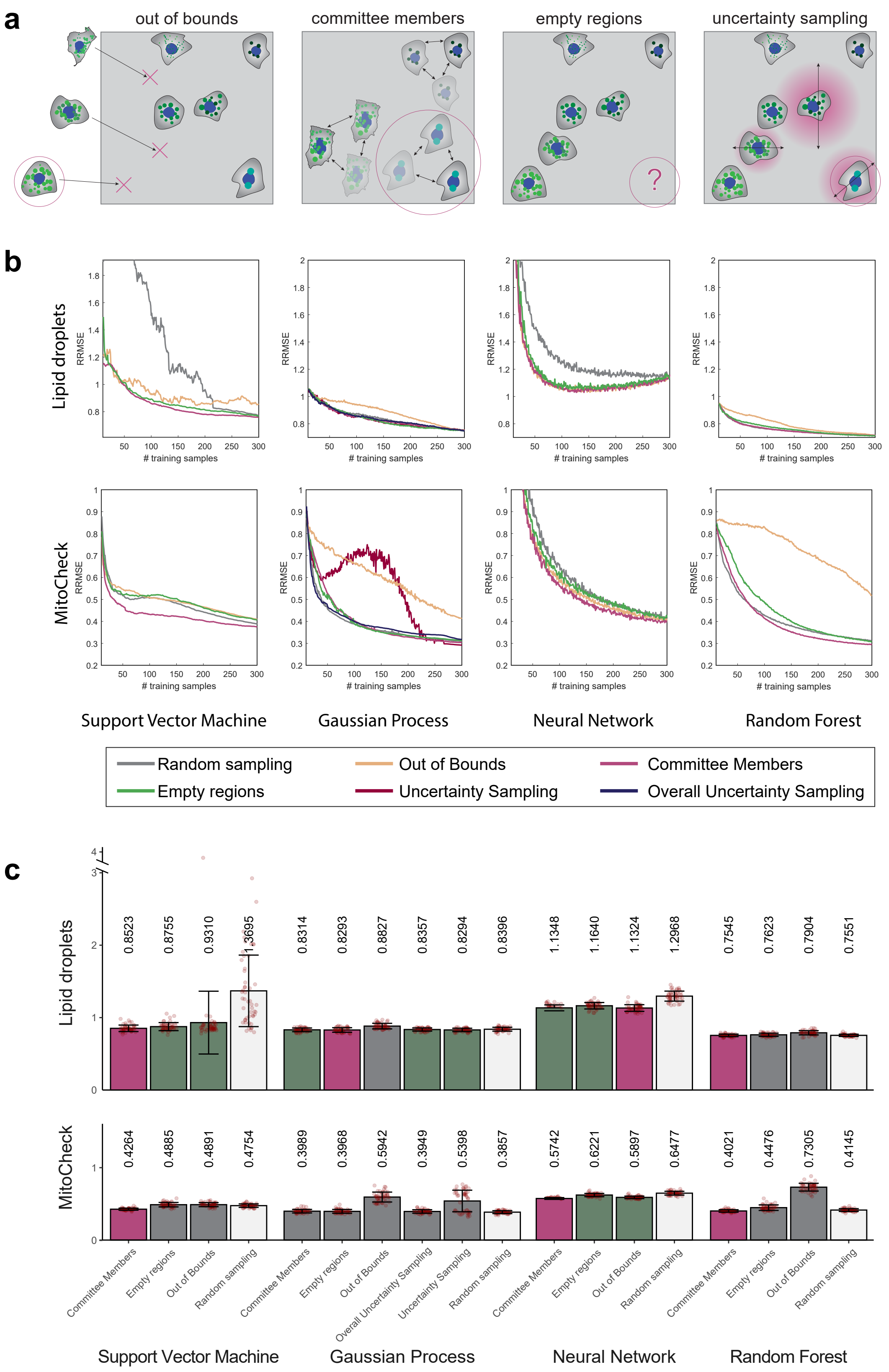

**Supplementary Figure 1: Active regression.** **(a)** Schematic representation of four active regression algorithms implemented in ACC. **(b)** Performance of the proposed methods measured as *Relative Root Mean Squared Error* (RRMSE) **(c)** Performance of the proposed methods measured as the average *Area Under RRMSE Curve* (lower is better). Columns represent the mean, error bars the standard deviation from n=50 independent runs. Methods showing superior performance to random sampling are highlighted with green, and the best among these with pink. The plots in both *(b)* and *(c)* represent the mean from 50 independent runs. Gaussian Processes were trained with constant mean function, squared exponential covariance function with automatic relevance determination (covSEard) for Lipids and with isotropic distance (covSEiso) for MitoCheck. The Neural Networks were trained with a single layer containing 30 nodes with log-sigmoid activation function. Random Forest and Support Vector Machine were trained with default parameters from Weka. The size of the committee in the CommitteeMembers method was 3. Source data are provided as a Source Data file.

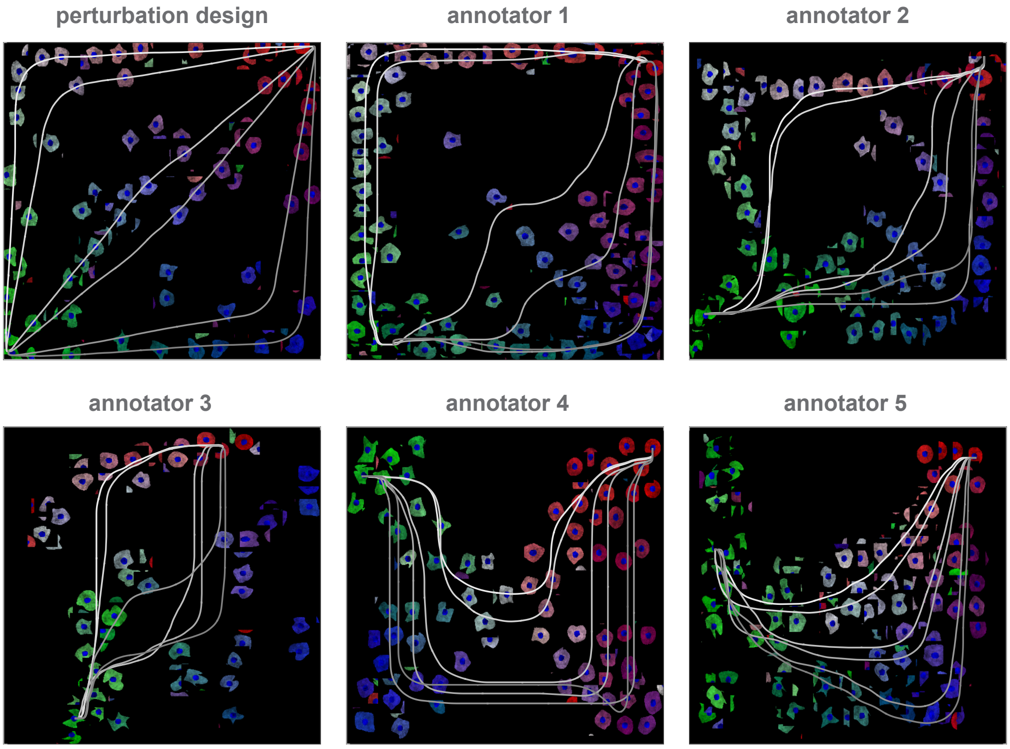


**Supplementary Figure 2: Synthetic dataset: Regression Planes.** The ground truth processes overlayed on a regression plane corresponding to the perturbation design and the annotations created by the 5 microscopy experts (*i.e.* annotators) who analysed the synthetic dataset with RP. The grey lines represent the identified processes and have been computed using a Kernel Density Estimation function and an energy minimization algorithm for finding the shortest path between process endpoints, using Dijkstra's algorithm. Despite the great variety of the regression planes generated by the annotators, in all the cases except for *annotator 1*, the six non-latent continuous processes are represented by separated lines.

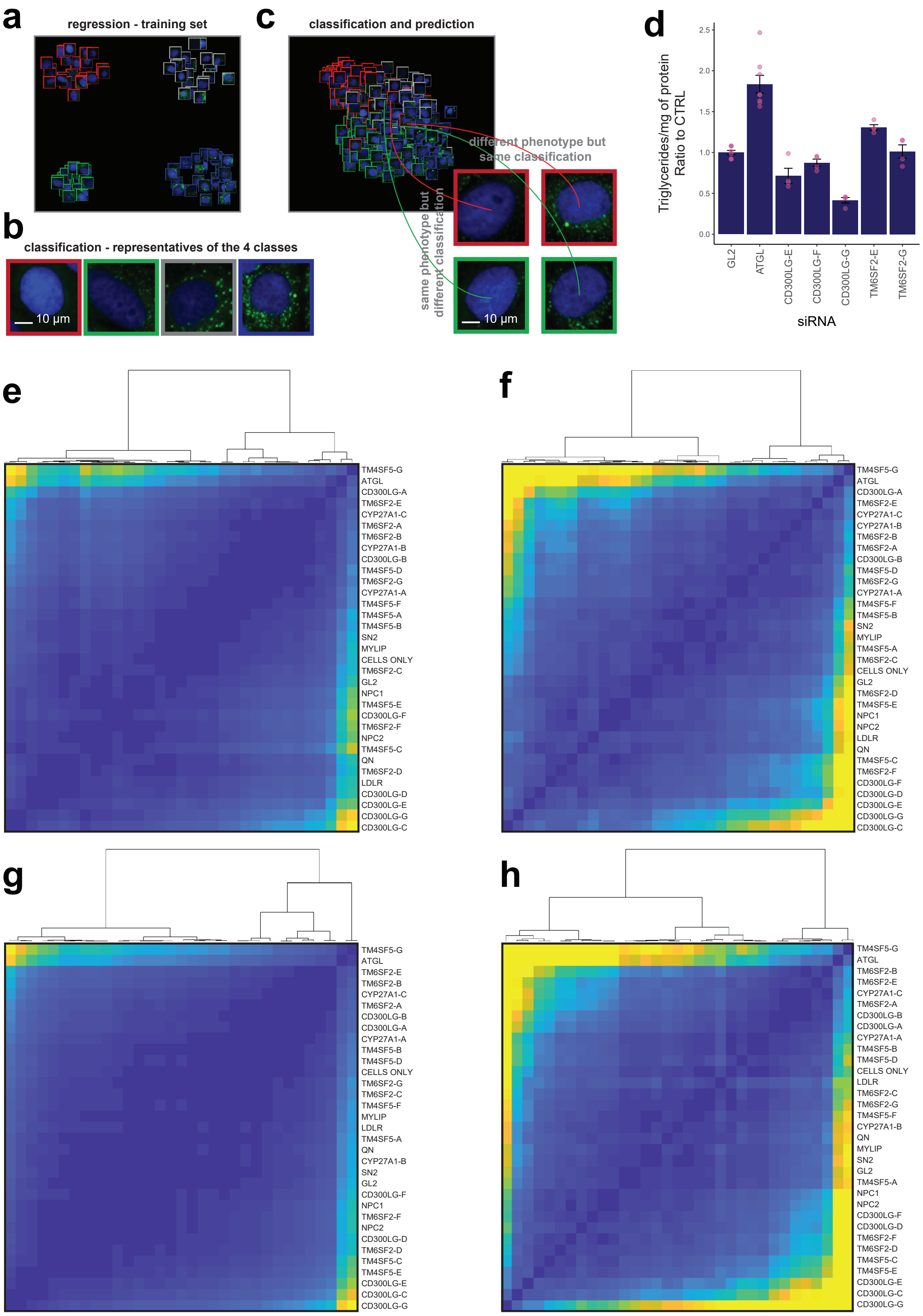
a
regression - training set
c
classification and prediction
different phenotype but
same classification
same phenotype but
different classification
b
classification - representatives of the 4 classes
10 µm
10 µm
d
Triglycerides/mg of protein
Ratio to CTRL
0.0
0.5
1.0
1.5
2.0
2.5
GL2
ATGL
CD300LG-E
CD300LG-F
CD300LG-G
TM6SF2-E
TM6SF2-G
siRNA
e
TM4SF5-G
ATGL
CD300LG-A
TM6SF2-E
CYP27A1-C
TM6SF2-A
TM6SF2-B
CYP27A1-B
CD300LG-B
TM4SF5-D
TM6SF2-G
CYP27A1-A
TM4SF5-F
TM4SF5-A
TM4SF5-B
SN2
MYLIP
CELLS ONLY
TM6SF2-C
GL2
NPC1
TM4SF5-E
CD300LG-F
TM6SF2-F
NPC2
TM4SF5-C
QN
TM6SF2-D
LDLR
CD300LG-D
CD300LG-E
CD300LG-G
CD300LG-C
f
TM4SF5-G
ATGL
CD300LG-A
TM6SF2-E
CYP27A1-C
CYP27A1-B
TM6SF2-B
TM6SF2-A
CD300LG-B
TM4SF5-D
TM6SF2-G
CYP27A1-A
TM4SF5-F
TM4SF5-B
SN2
MYLIP
TM4SF5-A
TM6SF2-C
CELLS ONLY
GL2
TM6SF2-D
TM4SF5-E
NPC1
NPC2
LDLR
QN
TM4SF5-C
TM6SF2-F
CD300LG-F
CD300LG-D
CD300LG-E
CD300LG-G
CD300LG-C
g
TM4SF5-G
ATGL
TM6SF2-E
TM6SF2-B
CYP27A1-C
TM6SF2-A
CD300LG-B
CD300LG-A
CYP27A1-A
TM4SF5-B
TM4SF5-D
CELLS ONLY
TM6SF2-G
TM6SF2-C
TM4SF5-F
MYLIP
LDLR
TM4SF5-A
QN
CYP27A1-B
SN2
GL2
CD300LG-F
NPC1
TM6SF2-F
NPC2
CD300LG-D
TM6SF2-D
TM4SF5-C
TM4SF5-E
CD300LG-E
CD300LG-C
CD300LG-G
h
TM4SF5-G
ATGL
TM6SF2-B
TM6SF2-E
CYP27A1-C
TM6SF2-A
CD300LG-B
CD300LG-A
CYP27A1-A
TM4SF5-B
TM4SF5-D
CELLS ONLY
LDLR
TM6SF2-C
TM6SF2-G
TM4SF5-F
CYP27A1-B
QN
MYLIP
SN2
GL2
TM4SF5-A
NPC1
NPC2
CD300LG-F
CD300LG-D
TM6SF2-F
TM6SF2-D
TM4SF5-C
TM4SF5-E
CD300LG-E
CD300LG-C
CD300LG-G

**Supplementary Figure 3: Lipid Droplet data analysis. (a-c) Classification vs regression applied on real-world data. (a)** Starting from a regression plane including 457 cells created by a microscopy expert, we have automatically selected 25 cells that were the closest to the center of the 4 quadrants of the regression plane, representing the 4 main cell phenotypes. **(b)** To visualize the cells belonging to the 4 different classes, we used borders of different colours (i.e. red, green, grey, blue). **(c)** Next, we classified the unannotated cells, and simultaneously predicted their position in the regression plane. The test revealed several cases of misclassifications: some cells with the same phenotype were classified into different classes, while several cells with a clearly different phenotype were classified into the same class. The experiment was repeated n=6 times independently with similar results. **(d) Biochemical analysis.** Intracellular TG levels (mean and standard error of 4 samples/condition from 2 independent experiments) in cultured hepatocytes (Huh7). siRNA-mediated knockdown of *TM6SF2* gene led to an increased level of TGs. In contrast, siRNAs targeting *CD300LG* decreased intracellular TG levels. Source data are provided as a Source Data file. *(e-h) Discovery tool: clustergram.* Clustergrams obtained by calculating symmetric Kullback-Leibler divergence for the KDE-maps (regression) / class probability distributions (classification) of the wells treated with different siRNAs. Bright (yellow) values indicate high divergence, meaning that the cells in the wells compared to each other have different morphologies. Clustergrams from regression show higher variation in the divergence values, better capturing subtle differences in the cell populations treated with siRNAs. **(e)** *Plate 01*, classification; **(f)** *Plate 01*, regression; **(g)** *Plate 02*, classification results; **(h)** *Plate 02*, regression results.

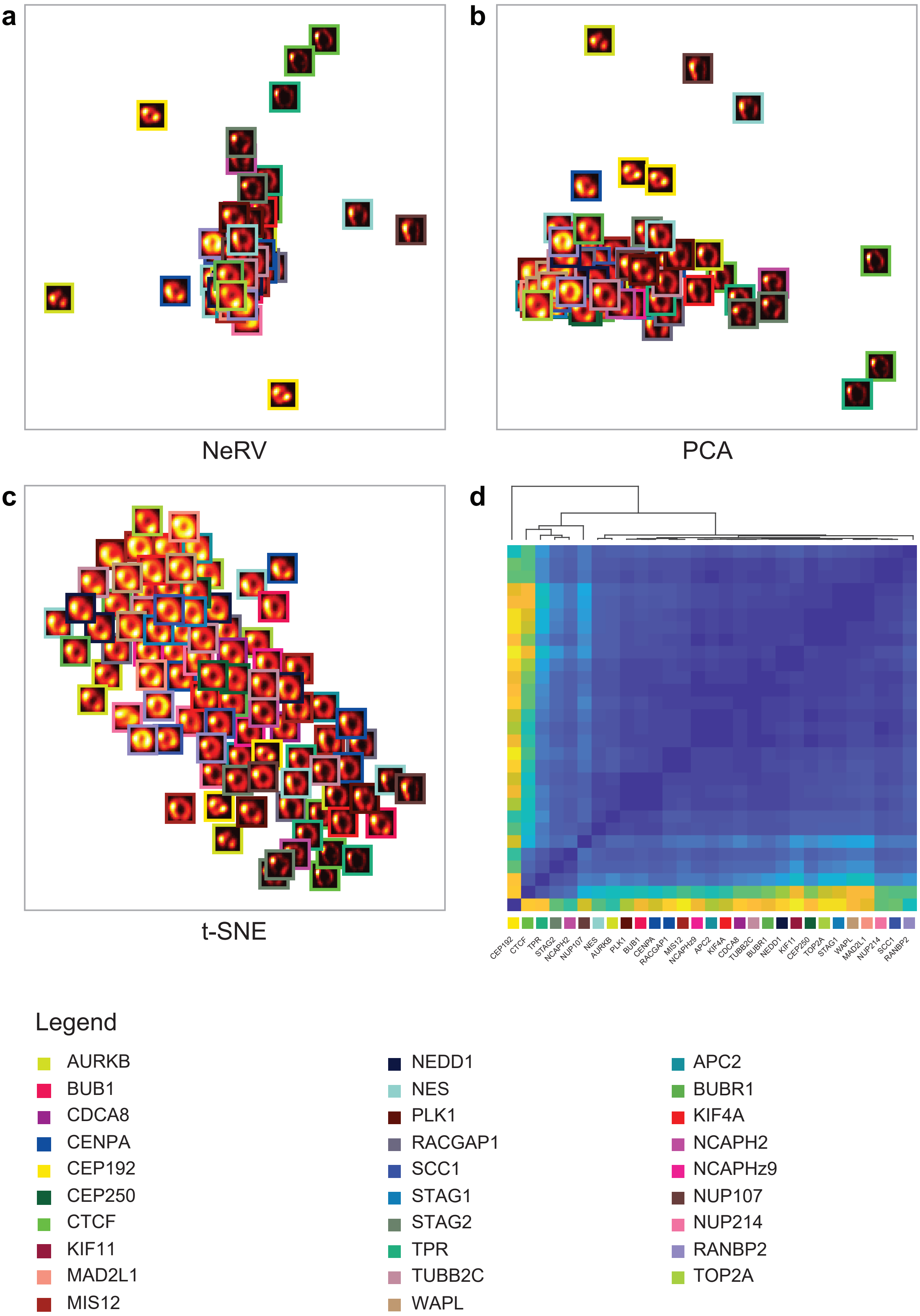
a
NeRV
b
PCA
c
t-SNE
d
CEP192
CTCF
TPR
STAG2
NCAPH2
NUP107
NES
AURKB
PLK1
BUB1
CENPA
RACGAP1
MIS12
NCAPHz9
APC2
KIF4A
CDCA8
TUBB2C
BUBR1
NEDD1
KIF11
CEP250
TOP2A
STAG1
WAPL
MAD2L1
NUP214
SCC1
RANBP2
Legend
AURKB
BUB1
CDCA8
CENPA
CEP192
CEP250
CTCF
KIF11
MAD2L1
MIS12
NEDD1
NES
PLK1
RACGAP1
SCC1
STAG1
STAG2
TPR
TUBB2C
WAPL
APC2
BUBR1
KIF4A
NCAPH2
NCAPHz9
NUP107
NUP214
RANBP2
TOP2A

**Supplementary Figure 4: The effect of gene knock-in on regression plane distribution. (a-c) Unsupervised visualization methods.** Abbreviations: (a) NeRV: Neighbour Retrieval Visualizer (b) PCA: Principal Component Analysis (c) t-SNE: t-distributed stochastic neighbour embedding. Each technical replicate (folder) in the original dataset is represented by a regression plane icon. Altogether 29 different protein tagging were analysed, with 2-5 replicate in each case. The icons are kernel density estimations of the predicted distributions, the colour of their frame represents the knocked-in gene corresponding to the legend in the bottom. Each folder contained 1-11 cells (each cell was followed for 40 frames), the median was 5 cells in a folder. **(d) Hierarchical clustering.** Technical replicates of the same knocked-in gene were aggregated and pairwise symmetric Kullback-Leibler divergence were calculated (heatmap). The hierarchical clustering is performed on the pairwise KLD matrix with correlation as distance and average linkage. We note that the tagging of CEP192 resulted in a different distribution on the regression plane according to PCA, NeRV and hierarchical clustering.

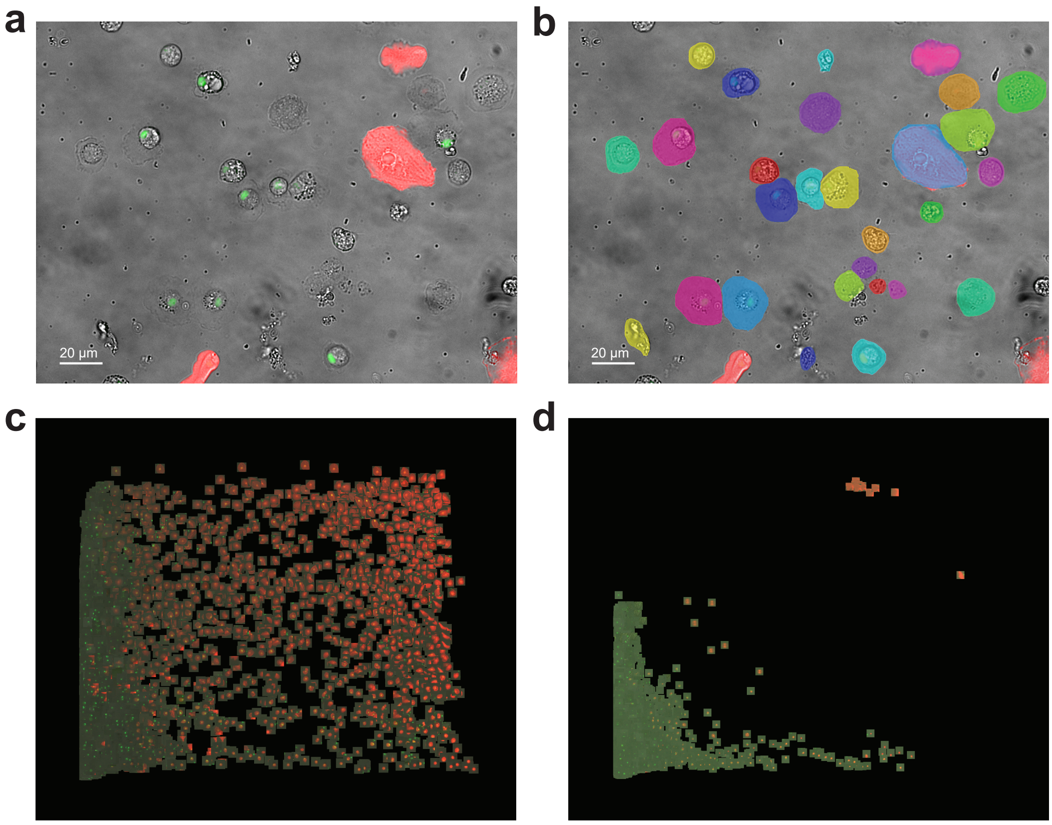


**Supplementary Figure 5: Blood Cells data validation and segmentation. (a)** Exemplary composite image with 3 channels: brightfield (grey), GFP (green), mCherry (red). **(b)** Corresponding results of deep-learning segmentation performed on the brightfield channel. **(c)** Prediction of all the immune induced cells on the regression plane. **(d)** Prediction of all cells from the control experiment on the regression plane.

## Supplementary Note 1.

## Synthetic data experiment configuration

All the microscopy experts (n=10) participating in the experiment received a live tutorial on '*How to use ACC*'. The microscopists (n=5) using RP also received a second tutorial on '*How to use the regression plane*'. In this second tutorial they were trained on how to use the *plot of plots* and the *clustergram* features of RP (**Supplementary Software 1** and **Supplementary Movie 2**). To perform the experiment all the microscopy experts downloaded the synthetic dataset from: *https://data.broadinstitute.org/bbbc/image_sets.html* (dataset ID: BBBC031). Then, the experts using the standard classification approaches (n=5) downloaded *ACC v.2.11* from: *www.cellclassifier.org*, whilst those using regression (n=5) downloaded *ACC v3.0*. We provided a Microsoft Excel sheet with an empty layout of the plate, and asked all the experts to assign a number (starting from *1*) to all the wells belonging to an identified process. We provided the 5 microscopists using the standard classification approaches with a MATLAB script (**Supplementary Software 3**) to cluster their data based on the class probability distribution per well, in order to compensate for the more advanced output opportunities available in RP. Meanwhile, the 5 microscopists using the regression approach were asked to create a single regression class in RP. Finally, we gave a limited time of two hours at a maximum to all the microscopists to explore the dataset.

# Supplementary Note 2.

# Experiment specific Image Analysis pipelines and Regression models

## I. Synthetic Data set (SIMCEP, BBBC031)

### A. Image Segmentation

The exact pipeline is provided in **Supplementary Software 2.**

Nuclei and cytoplasm was detected using the ground truth masks generated by SIMCEP.

### B. Feature Extraction

Standard intensity and morphological features were extracted from the detected cells. The full list of features is provided in **Section V/1** at the end of this file.

### C. Regression (Classification) Models

In this experiment each expert microscopist (user) chose the regression model (in case of the control group, classification model) he/she considered the best.

(1) The 5 classification models selected by the control group users:

- Multilayer Perceptron [MLP_Weka] (3x)
- Random Forest [RandomForest_Weka] (2x)

(2) The 5 regression models selected by the test group users:

- Gaussian Processes [GPMLPredictor - meanConst, covSEard] (3x)
- Linear Regression [WekaLinearRegression]
- Multilayer Perceptron [WekaMLP]

## II. <u>Lipid droplet dataset (siRNA assay)</u>

### A. Image Segmentation

Nuclei were identified using Otsu Adaptive Thresholding. Found objects were filtered to have diameter between 20 and 150. Lower and upper bounds on the threshold was 0.5044,0.505 respectively. Clumped objects were distinguished by the *Shape* method, and divided by the *Intensity* method.

Cytoplasms were identified as a 35 pixel wide rings around the detected nuclei.

The full CellProfiler pipeline is provided in **Section V/2** at the end of this file as text.

### B. Feature Extraction

Standard intensity features were extracted from the nuclei and cytoplasm objects both on the DAPI and the LipidTOX channel.

Morphological features were extracted from the nuclei.

Texture features with scale=3 were extracted from both nuclei and cytoplasm objects on both the DAPI and the LipidTOX channel.

The full list of extracted features is available online at:

https://doi.org/10.6084/m9.figshare.c.5067638.v1

The full list of features can be found in the ACC Project folders under:

LipidDroplets-siRNA-plate0[1-2]/anal2/featureNames.acc

### C. Regression Models

The plots for the Lipid dataset in Figure 2. were created with the Gaussian Processes (GPMLPredictor - meanConst, covSEard) model without feature normalization.

The used control was Qiagen Negative [QN] (for further info see **Supplementary Data 1)**

The results for Supplementary Figure 3a-c were created with Random Forest (both classification [RandomForest_Weka] and regression [WekaRandomForest]).

The dendograms were created with the best models assessed by 10 fold cross validation.

(1) Classification:

- Plate 01: Multilayer Perceptron [MLP_Weka]
- Plate 02: additive logistic regression [LogitBoost_Weka]

(2) Regression:

- Plate 01: Multi Stack Learner [MulanMTStack – SMO_Regression, RandomForest]
- Plate 02: Multi Stack Learner [MulanMTStack – GuassianProcesses, RandomForest]

## III. MitoCheck dataset

### A. Image Segmentation

The public dataset of this experiment contained the cell masks as well, hence there was no need for image segmentation.

The data is publicly available at:

http://www.mitocheck.org/mitotic_cell_atlas/downloads/v1.0.1/mitotic_cell_atlas_v1.0.1_src.zip

### B. Feature Extraction

3D images were maximum projected to extract standard features in 2D. Morphological features were extracted from both the nuclei and cytoplasm objects. Texture features with scale=[3,5] were extracted from the nuclear staining. Additionally, the features were extended with the first order derivatives (calculated with $1^{st}$ order central difference on 2 scales: h = [1,3]) of all features with respect to the cell-trajectories. The full list of extracted features is provided in **Section V/3** at the end of this file as text.

### C. Regression Models

In case of the MitoCheck dataset we used Random Forest as the Regression Model.

## IV. Drosophila blood cell differentiation

### A. Image Analysis

Cell segmentation was carried out by the commercial BIAS software (https://single-cell-technologies.com/bias/), using a deep convolutional neural network (structure of the network is based on maskRCNN, the exact model is described in: Hollandi et al. nucleAIzer: A parameter-free deep learning framework for nucleus segmentation using image style transfer. Cell Systems, 10(5), 453-458 (2020). The nucleAIzer framework was trained with a manually annotated set of selected brightfield images from the experiment to find the cells directly (no nuclei identification was done).

### B. Feature Extraction

Morphological features were extracted from the identified cells. Intensity features were extracted from all channels.

The full list of extracted features is available online at:

https://doi.org/10.6084/m9.figshare.c.5075093.v1

The list of the extracted features can be found in the ACC Project folders under:

[plate01_induced/plate02_control]/anal2/featureNames.acc

### C. Regression Models

In case of the Drosophila blood cell dataset we used Random Forest as the Regression Model.

## V. Lists of extracted features and pipelines:

**1) Synthetic data extracted features.**

Cells.Intensity_OrigBlueFeatures.IntegratedIntensity
Cells.Intensity_OrigBlueFeatures.MeanIntensity
Cells.Intensity_OrigBlueFeatures.StdIntensity
Cells.Intensity_OrigBlueFeatures.MinIntensity
Cells.Intensity_OrigBlueFeatures.MaxIntensity
Cells.Intensity_OrigBlueFeatures.IntegratedIntensityEdge
Cells.Intensity_OrigBlueFeatures.MeanIntensityEdge
Cells.Intensity_OrigBlueFeatures.StdIntensityEdge
Cells.Intensity_OrigBlueFeatures.MinIntensityEdge
Cells.Intensity_OrigBlueFeatures.MaxIntensityEdge
Cells.Intensity_OrigBlueFeatures.MassDisplacement
Cells.Intensity_OrigRedFeatures.IntegratedIntensity
Cells.Intensity_OrigRedFeatures.MeanIntensity
Cells.Intensity_OrigRedFeatures.StdIntensity
Cells.Intensity_OrigRedFeatures.MinIntensity
Cells.Intensity_OrigRedFeatures.MaxIntensity
Cells.Intensity_OrigRedFeatures.IntegratedIntensityEdge
Cells.Intensity_OrigRedFeatures.MeanIntensityEdge
Cells.Intensity_OrigRedFeatures.StdIntensityEdge
Cells.Intensity_OrigRedFeatures.MinIntensityEdge
Cells.Intensity_OrigRedFeatures.MaxIntensityEdge
Cells.Intensity_OrigRedFeatures.MassDisplacement
Cells.Intensity_OrigGreenFeatures.IntegratedIntensity
Cells.Intensity_OrigGreenFeatures.MeanIntensity
Cells.Intensity_OrigGreenFeatures.StdIntensity
Cells.Intensity_OrigGreenFeatures.MinIntensity
Cells.Intensity_OrigGreenFeatures.MaxIntensity
Cells.Intensity_OrigGreenFeatures.IntegratedIntensityEdge

Cells.Intensity_OrigGreenFeatures.MeanIntensityEdge

Cells.Intensity_OrigGreenFeatures.StdIntensityEdge

Cells.Intensity_OrigGreenFeatures.MinIntensityEdge

Cells.Intensity_OrigGreenFeatures.MaxIntensityEdge

Cells.Intensity_OrigGreenFeatures.MassDisplacement

Cells.AreaShapeFeatures.Area

Cells.AreaShapeFeatures.Eccentricity

Cells.AreaShapeFeatures.Solidity

Cells.AreaShapeFeatures.Extent

Cells.AreaShapeFeatures.EulerNumber

Cells.AreaShapeFeatures.Perimeter

Cells.AreaShapeFeatures.FormFactor

Cells.AreaShapeFeatures.MajorAxisLength

Cells.AreaShapeFeatures.MinorAxisLength

Cells.AreaShapeFeatures.Orientation

## 2) Lipid droplets pipeline as text:

Saved Pipeline, in file LipidDroplets_RingDetectionPipe.txt, Saved on 31-Aug-2020

Pixel Size: 1

Pipeline:
LoadImages
RescaleIntensity
RescaleIntensity
RescaleIntensity
IdentifyPrimAutomatic
IdentifySecondary
DetectSpots
DetectSpots
OverlayOutlines
OverlayOutlines
OverlayOutlines
OverlayOutlines
GrayToColor

SaveImages
GrayToColor
SaveImages
MeasureObjectIntensity
MeasureObjectIntensity
MeasureObjectAreaShape
MeasureTexture
MeasureTexture
Relate
Relate
ExportToACC

Module #1: LoadImages revision - 2
How do you want to load these files? Text-Exact match
Type the text that one type of image has in common (for TEXT options), or their position in each group (for ORDER option): Hoechst
What do you want to call these images within CellProfiler? OrigBlue
Type the text that one type of image has in common (for TEXT options), or their position in each group (for ORDER option): Alexa488
What do you want to call these images within CellProfiler? OrigGreen
Type the text that one type of image has in common (for TEXT options), or their position in each group (for ORDER option): /
What do you want to call these images within CellProfiler? /
Type the text that one type of image has in common (for TEXT options), or their position in each group (for ORDER option): /
What do you want to call these images within CellProfiler? /
If using ORDER, how many images are there in each group (i.e. each field of view)? 3
What type of files are you loading? individual images
Analyze all subfolders within the selected folder? No
Enter the path name to the folder where the images to be loaded are located. Type period (.) for default image folder. .
Note - If the movies contain more than just one image type (e.g., brightfield, fluorescent, field-of-view), add the GroupMovieFrames module. n/a

Module #2: RescaleIntensity revision - 2
What did you call the image to be rescaled? OrigBlue
What do you want to call the rescaled image? RescaledBlue
Rescaling method. (S) Stretch the image (0 to 1). (E) Enter the minimum and maximum values in the boxes below. (G) rescale so all pixels are equal to or Greater than one. (M) Match the maximum of one image to the maximum of another. (C) Convert to 8 bit. See the help for details. Enter min/max below
(Method E only): Enter the intensity from the original image that should be set to the lowest value in the rescaled image, or type AA to calculate the lowest intensity automatically from all of the images to be analyzed and AE to calculate the lowest intensity from each image independently. 0.5040

(Method E only): Enter the intensity from the original image that should be set to the highest value in the rescaled image, or type AA to calculate the highest intensity automatically from all of the images to be analyzed and AE to calculate the highest intensity from each image independently.    0.52

(Method E only): What should the lowest intensity of the rescaled image be (range [0,1])?    0

(Method E only): What should the highest intensity of the rescaled image be (range [0,1])?    1

(Method M only): What did you call the image whose maximum you want the rescaled image to match?    OrigBlue

Module #3: RescaleIntensity revision - 2

What did you call the image to be rescaled?    OrigGreen

What do you want to call the rescaled image?    RescaledGreen

Rescaling method. (S) Stretch the image (0 to 1). (E) Enter the minimum and maximum values in the boxes below. (G) rescale so all pixels are equal to or Greater than one. (M) Match the maximum of one image to the maximum of another. (C) Convert to 8 bit. See the help for details.    Enter min/max below

(Method E only): Enter the intensity from the original image that should be set to the lowest value in the rescaled image, or type AA to calculate the lowest intensity automatically from all of the images to be analyzed and AE to calculate the lowest intensity from each image independently.    0.505

(Method E only): Enter the intensity from the original image that should be set to the highest value in the rescaled image, or type AA to calculate the highest intensity automatically from all of the images to be analyzed and AE to calculate the highest intensity from each image independently.    .53

(Method E only): What should the lowest intensity of the rescaled image be (range [0,1])?    0

(Method E only): What should the highest intensity of the rescaled image be (range [0,1])?    1

(Method M only): What did you call the image whose maximum you want the rescaled image to match?    OrigGreen

Module #4: RescaleIntensity revision - 2

What did you call the image to be rescaled?    OrigBlue

What do you want to call the rescaled image?    RescaledRed

Rescaling method. (S) Stretch the image (0 to 1). (E) Enter the minimum and maximum values in the boxes below. (G) rescale so all pixels are equal to or Greater than one. (M) Match the maximum of one image to the maximum of another. (C) Convert to 8 bit. See the help for details.    Enter min/max below

(Method E only): Enter the intensity from the original image that should be set to the lowest value in the rescaled image, or type AA to calculate the lowest intensity automatically from all of the images to be analyzed and AE to calculate the lowest intensity from each image independently.    0.9

(Method E only): Enter the intensity from the original image that should be set to the highest value in the rescaled image, or type AA to calculate the highest intensity automatically from all of the images to be analyzed and AE to calculate the highest intensity from each image independently.    0.91

(Method E only): What should the lowest intensity of the rescaled image be (range [0,1])? 0
(Method E only): What should the highest intensity of the rescaled image be (range [0,1])? 1
(Method M only): What did you call the image whose maximum you want the rescaled image to match? OrigBlue

Module #5: IdentifyPrimAutomatic revision - 12
What did you call the images you want to process? OrigBlue
What do you want to call the objects identified by this module? Nuclei
Typical diameter of objects, in pixel units (Min,Max): 20,150
Discard objects outside the diameter range? Yes
Try to merge too small objects with nearby larger objects? Yes
Discard objects touching the border of the image? Yes
Select an automatic thresholding method or enter an absolute threshold in the range [0,1]. To choose a binary image, select "Other" and type its name. Choosing 'All' will use the Otsu Global method to calculate a single threshold for the entire image group. The other methods calculate a threshold for each image individually. "Set interactively" will allow you to manually adjust the threshold during the first cycle to determine what will work well. Otsu Adaptive
Threshold correction factor 1
Lower and upper bounds on threshold, in the range [0,1] 0.5044,0.505
For MoG thresholding, what is the approximate fraction of image covered by objects? 0.1
Method to distinguish clumped objects (see help for details): Shape
Method to draw dividing lines between clumped objects (see help for details): Intensity
Size of smoothing filter, in pixel units (if you are distinguishing between clumped objects). Enter 0 for low resolution images with small objects (~< 5 pixel diameter) to prevent any image smoothing. Automatic
Suppress local maxima within this distance, (a positive integer, in pixel units) (if you are distinguishing between clumped objects) Automatic
Speed up by using lower-resolution image to find local maxima? (if you are distinguishing between clumped objects) Yes
Enter the following information, separated by commas, if you would like to use the Laplacian of Gaussian method for identifying objects instead of using the above settings: Size of neighborhood(height,width),Sigma,Minimum Area,Size for Wiener Filter(height,width),Threshold /
What do you want to call the outlines of the identified objects (optional)? CellOutline
Do you want to fill holes in identified objects? Yes
Do you want to run in test mode where each method for distinguishing clumped objects is compared? No

Module #6: IdentifySecondary revision - 3
What did you call the primary objects you want to create secondary objects around? Nuclei
What do you want to call the objects identified by this module? Cells
Select the method to identify the secondary objects (Distance - B uses background; Distance - N does not): Distance - N
What did you call the images to be used to find the edges of the secondary objects? For DISTANCE - N, this will not affect object identification, only the final display. OrigGreen

Select an automatic thresholding method or enter an absolute threshold in the range [0,1]. To choose a binary image, select "Other" and type its name. Choosing 'All' will use the Otsu Global method to calculate a single threshold for the entire image group. The other methods calculate a threshold for each image individually. Set interactively will allow you to manually adjust the threshold during the first cycle to determine what will work well.    Otsu Adaptive
Threshold correction factor    1
Lower and upper bounds on threshold, in the range [0,1]    0.5042,0.50545
For MoG thresholding, what is the approximate fraction of image covered by objects?    0.01
For DISTANCE, enter the number of pixels by which to expand the primary objects [Positive integer]    35
For PROPAGATION, enter the regularization factor (0 to infinity). Larger=distance,0=intensity    0.05
What do you want to call the outlines of the identified objects (optional)?    CellOut
Do you want to run in test mode where each method for identifying secondary objects is compared?    No

Module #7: DetectSpots revision - 3
What did you call the images you want to process?    RescaledGreen
What do you want to call the spots identified by this module?    Spots
Size of the A Trous wavelet?    2
Size of smoothing filter, in pixel units.    3
Noise removal factor (removes background + n times std).    3
Static threshold for spot identificaiton.    0.005
What do you want to call the outlines of the identified objects (optional)?    Spots

Module #8: DetectSpots revision - 3
What did you call the images you want to process?    RescaledGreen
What do you want to call the spots identified by this module?    BigSpots
Size of the A Trous wavelet?    2
Size of smoothing filter, in pixel units.    3
Noise removal factor (removes background + n times std).    3
Static threshold for spot identificaiton.    0.03
What do you want to call the outlines of the identified objects (optional)?    BigSpotOutline

Module #9: OverlayOutlines revision - 2
On which image would you like to display the outlines?    RescaledBlue
What did you call the outlines that you would like to display?    CellOut
Would you like to set the intensity (brightness) of the outlines to be the same as the brightest point in the image, or the maximum possible value for this image format?    Max possible
What do you want to call the image with the outlines displayed?    NucleiiImage
For color images, what do you want the color of the outlines to be?    White

Module #10: OverlayOutlines revision - 2
On which image would you like to display the outlines?    RescaledGreen
What did you call the outlines that you would like to display?    CellOutline

Would you like to set the intensity (brightness) of the outlines to be the same as the brightest point in the image, or the maximum possible value for this image format?    Max possible
What do you want to call the image with the outlines displayed?    CytoImage
For color images, what do you want the color of the outlines to be?    White

Module #11: OverlayOutlines revision - 2
On which image would you like to display the outlines?    RescaledRed
What did you call the outlines that you would like to display?    Spots
Would you like to set the intensity (brightness) of the outlines to be the same as the brightest point in the image, or the maximum possible value for this image format?    Max possible
What do you want to call the image with the outlines displayed?    SpotImage
For color images, what do you want the color of the outlines to be?    White

Module #12: OverlayOutlines revision - 2
On which image would you like to display the outlines?    NucleiiImage
What did you call the outlines that you would like to display?    BigSpotOutline
Would you like to set the intensity (brightness) of the outlines to be the same as the brightest point in the image, or the maximum possible value for this image format?    Max possible
What do you want to call the image with the outlines displayed?    NicleiBigSpotImage
For color images, what do you want the color of the outlines to be?    White

Module #13: GrayToColor revision - 2
What did you call the image to be colored red?    SpotImage
What did you call the image to be colored green?    CytoImage
What did you call the image to be colored blue?    NicleiBigSpotImage
What do you want to call the resulting image?    ColorImage
Enter the adjustment factor for the red image    1
Enter the adjustment factor for the green image    1
Enter the adjustment factor for the blue image    1

Module #14: SaveImages revision - 12
What did you call the images you want to save? If you would like to save an entire figure, enter the module number here    ColorImage
Which images' original filenames do you want use as a base for these new images' filenames? Your choice MUST be images loaded directly with a Load module. Alternately, type N to use sequential numbers for the file names, or type =DesiredFilename to use the single file name you specify (replace DesiredFilename with the name you actually want) for all files (this is *required* when saving an avi movie).    OrigBlue
Enter text to append to the image name, type N to use sequential numbers, or leave "" to not append anything.    \n    In what file format do you want to save images (figures must be saved as fig, which is only openable in Matlab)?    png
Enter the pathname to the directory where you want to save the images.  Type period (.) for default output directory or ampersand (&) for the directory of the original image.    &/anal1
Enter the bit depth at which to save the images (Note: some image formats do not support saving at a bit depth of 12 or 16; see Matlab's imwrite function for more details.)    8

Do you want to always check whether you will be overwriting a file when saving images? No
At what point in the pipeline do you want to save the image? When saving in avi (movie) format, choose Every cycle.    Every cycle
If you are saving in avi (movie) format, do you want to save the movie only after the last cycle is processed (enter 'L'), or after every Nth cycle (1,2,3...)? Saving movies is time-consuming. See the help for this module for more details.    L
Do you want to rescale the images to use a full 8 bit (256 graylevel) dynamic range (Y or N)? Use the RescaleIntensity module for other rescaling options.    No
For grayscale images, specify the colormap to use (see help). This is critical for movie (avi) files. Choosing anything other than gray may degrade image quality or result in image stretching. gray
Enter any optional parameters here ('Quality',1 or 'Quality',100 etc.) or leave / for no optional parameters.    /
Update file names within CellProfiler? See help for details.    No
Warning! It is possible to overwrite existing files using this module!    n/a

Module #15: GrayToColor revision - 2
What did you call the image to be colored red?    RescaledRed
What did you call the image to be colored green?    RescaledGreen
What did you call the image to be colored blue?    RescaledBlue
What do you want to call the resulting image?    ColorOrigImage
Enter the adjustment factor for the red image    1
Enter the adjustment factor for the green image    1
Enter the adjustment factor for the blue image    1

Module #16: SaveImages revision - 12
What did you call the images you want to save? If you would like to save an entire figure, enter the module number here    ColorOrigImage
Which images' original filenames do you want use as a base for these new images' filenames? Your choice MUST be images loaded directly with a Load module. Alternately, type N to use sequential numbers for the file names, or type =DesiredFilename to use the single file name you specify (replace DesiredFilename with the name you actually want) for all files (this is *required* when saving an avi movie).    OrigBlue
Enter text to append to the image name, type N to use sequential numbers, or leave "" to not append anything.    \n    In what file format do you want to save images (figures must be saved as fig, which is only openable in Matlab)?    png
Enter the pathname to the directory where you want to save the images. Type period (.) for default output directory or ampersand (&) for the directory of the original image.    &/anal3
Enter the bit depth at which to save the images (Note: some image formats do not support saving at a bit depth of 12 or 16; see Matlab's imwrite function for more details.)    8
Do you want to always check whether you will be overwriting a file when saving images? No
At what point in the pipeline do you want to save the image? When saving in avi (movie) format, choose Every cycle.    Every cycle

If you are saving in avi (movie) format, do you want to save the movie only after the last cycle is processed (enter 'L'), or after every Nth cycle (1,2,3...)? Saving movies is time-consuming. See the help for this module for more details. L

Do you want to rescale the images to use a full 8 bit (256 graylevel) dynamic range (Y or N)? Use the RescaleIntensity module for other rescaling options. No

For grayscale images, specify the colormap to use (see help). This is critical for movie (avi) files. Choosing anything other than gray may degrade image quality or result in image stretching. gray

Enter any optional parameters here ('Quality',1 or 'Quality',100 etc.) or leave / for no optional parameters. /

Update file names within CellProfiler? See help for details. No

Warning! It is possible to overwrite existing files using this module! n/a

Module #17: MeasureObjectIntensity revision - 2

What did you call the greyscale images you want to measure? OrigGreen

What did you call the objects that you want to measure? Nuclei

Cells

Spots

BigSpots

Do not use

Do not use

Module #18: MeasureObjectIntensity revision - 2

What did you call the greyscale images you want to measure? OrigBlue

What did you call the objects that you want to measure? Nuclei

Cells

Do not use

Do not use

Do not use

Do not use

Module #19: MeasureObjectAreaShape revision - 3

What did you call the objects that you want to measure? Nuclei

Do not use

Do not use

Do not use

Do not use

Do not use

Do not use

Would you like to calculate the Zernike features for each object (with lots of objects, this can be very slow)? No

Module #20: MeasureTexture revision - 2

What did you call the greyscale images you want to measure? OrigBlue

What did you call the objects that you want to measure? Nuclei

Cells

Do not use
Do not use
Do not use
Do not use
What is the scale of texture?    3

Module #21: MeasureTexture revision - 2
What did you call the greyscale images you want to measure?    OrigGreen
What did you call the objects that you want to measure?    Nuclei
Cells
Do not use
Do not use
Do not use
Do not use
What is the scale of texture?    3

Module #22: Relate revision - 2
What objects are the children objects (subobjects)?    Spots
What are the parent objects?    Cells
What other object do you want to find distances to? (Must be one object per parent object, e.g. Nuclei)    Nuclei

Module #23: Relate revision - 2
What objects are the children objects (subobjects)?    BigSpots
What are the parent objects?    Cells
What other object do you want to find distances to? (Must be one object per parent object, e.g. Nuclei)    Nuclei

Module #24: ExportToACC revision - 3
Where do you want to save the measurements    anal2
Which images' original filenames do you want use as a base for the export? To make ACC work correctly you should set this to the same original filename with which you saved your images.    OrigBlue
Which objects do you want to export? Cell location will be calculted from the first object. Normally use here nuclei. (Object #1 - PRIMARY)    /
Object #2    /
Is this a multiple object?    No
Object #3    /
Is this a multiple object?    No
Object #4    /
Is this a multiple object?    No

**3) MitoCheck extracted features**

cyto_AreaShape_Area
cyto_AreaShape_Compactness
cyto_AreaShape_Eccentricity

cyto_AreaShape_Extent
cyto_AreaShape_FormFactor
cyto_AreaShape_MajorAxisLength
cyto_AreaShape_MaxFeretDiameter
cyto_AreaShape_MaximumRadius
cyto_AreaShape_MeanRadius
cyto_AreaShape_MedianRadius
cyto_AreaShape_MinFeretDiameter
cyto_AreaShape_MinorAxisLength
cyto_AreaShape_Perimeter
cyto_AreaShape_Solidity
cyto_Texture_AngularSecondMoment_Blue_3_00
cyto_Texture_AngularSecondMoment_Blue_3_01
cyto_Texture_AngularSecondMoment_Blue_3_02
cyto_Texture_AngularSecondMoment_Blue_3_03
cyto_Texture_AngularSecondMoment_Blue_5_00
cyto_Texture_AngularSecondMoment_Blue_5_01
cyto_Texture_AngularSecondMoment_Blue_5_02
cyto_Texture_AngularSecondMoment_Blue_5_03
cyto_Texture_Contrast_Blue_3_00
cyto_Texture_Contrast_Blue_3_01
cyto_Texture_Contrast_Blue_3_02
cyto_Texture_Contrast_Blue_3_03
cyto_Texture_Contrast_Blue_5_00
cyto_Texture_Contrast_Blue_5_01
cyto_Texture_Contrast_Blue_5_02
cyto_Texture_Contrast_Blue_5_03
cyto_Texture_Correlation_Blue_3_00
cyto_Texture_Correlation_Blue_3_01
cyto_Texture_Correlation_Blue_3_02
cyto_Texture_Correlation_Blue_3_03
cyto_Texture_Correlation_Blue_5_00
cyto_Texture_Correlation_Blue_5_01
cyto_Texture_Correlation_Blue_5_02
cyto_Texture_Correlation_Blue_5_03
cyto_Texture_DifferenceEntropy_Blue_3_00
cyto_Texture_DifferenceEntropy_Blue_3_01
cyto_Texture_DifferenceEntropy_Blue_3_02
cyto_Texture_DifferenceEntropy_Blue_3_03
cyto_Texture_DifferenceEntropy_Blue_5_00
cyto_Texture_DifferenceEntropy_Blue_5_01
cyto_Texture_DifferenceEntropy_Blue_5_02
cyto_Texture_DifferenceEntropy_Blue_5_03
cyto_Texture_DifferenceVariance_Blue_3_00
cyto_Texture_DifferenceVariance_Blue_3_01
cyto_Texture_DifferenceVariance_Blue_3_02

cyto_Texture_DifferenceVariance_Blue_3_03
cyto_Texture_DifferenceVariance_Blue_5_00
cyto_Texture_DifferenceVariance_Blue_5_01
cyto_Texture_DifferenceVariance_Blue_5_02
cyto_Texture_DifferenceVariance_Blue_5_03
cyto_Texture_Entropy_Blue_3_00
cyto_Texture_Entropy_Blue_3_01
cyto_Texture_Entropy_Blue_3_02
cyto_Texture_Entropy_Blue_3_03
cyto_Texture_Entropy_Blue_5_00
cyto_Texture_Entropy_Blue_5_01
cyto_Texture_Entropy_Blue_5_02
cyto_Texture_Entropy_Blue_5_03
cyto_Texture_InfoMeas1_Blue_3_00
cyto_Texture_InfoMeas1_Blue_3_01
cyto_Texture_InfoMeas1_Blue_3_02
cyto_Texture_InfoMeas1_Blue_3_03
cyto_Texture_InfoMeas1_Blue_5_00
cyto_Texture_InfoMeas1_Blue_5_01
cyto_Texture_InfoMeas1_Blue_5_02
cyto_Texture_InfoMeas1_Blue_5_03
cyto_Texture_InfoMeas2_Blue_3_00
cyto_Texture_InfoMeas2_Blue_3_01
cyto_Texture_InfoMeas2_Blue_3_02
cyto_Texture_InfoMeas2_Blue_3_03
cyto_Texture_InfoMeas2_Blue_5_00
cyto_Texture_InfoMeas2_Blue_5_01
cyto_Texture_InfoMeas2_Blue_5_02
cyto_Texture_InfoMeas2_Blue_5_03
cyto_Texture_InverseDifferenceMoment_Blue_3_00
cyto_Texture_InverseDifferenceMoment_Blue_3_01
cyto_Texture_InverseDifferenceMoment_Blue_3_02
cyto_Texture_InverseDifferenceMoment_Blue_3_03
cyto_Texture_InverseDifferenceMoment_Blue_5_00
cyto_Texture_InverseDifferenceMoment_Blue_5_01
cyto_Texture_InverseDifferenceMoment_Blue_5_02
cyto_Texture_InverseDifferenceMoment_Blue_5_03
cyto_Texture_SumAverage_Blue_3_00
cyto_Texture_SumAverage_Blue_3_01
cyto_Texture_SumAverage_Blue_3_02
cyto_Texture_SumAverage_Blue_3_03
cyto_Texture_SumAverage_Blue_5_00
cyto_Texture_SumAverage_Blue_5_01
cyto_Texture_SumAverage_Blue_5_02
cyto_Texture_SumAverage_Blue_5_03
cyto_Texture_SumEntropy_Blue_3_00

cyto_Texture_SumEntropy_Blue_3_01
cyto_Texture_SumEntropy_Blue_3_02
cyto_Texture_SumEntropy_Blue_3_03
cyto_Texture_SumEntropy_Blue_5_00
cyto_Texture_SumEntropy_Blue_5_01
cyto_Texture_SumEntropy_Blue_5_02
cyto_Texture_SumEntropy_Blue_5_03
cyto_Texture_SumVariance_Blue_3_00
cyto_Texture_SumVariance_Blue_3_01
cyto_Texture_SumVariance_Blue_3_02
cyto_Texture_SumVariance_Blue_3_03
cyto_Texture_SumVariance_Blue_5_00
cyto_Texture_SumVariance_Blue_5_01
cyto_Texture_SumVariance_Blue_5_02
cyto_Texture_SumVariance_Blue_5_03
cyto_Texture_Variance_Blue_3_00
cyto_Texture_Variance_Blue_3_01
cyto_Texture_Variance_Blue_3_02
cyto_Texture_Variance_Blue_3_03
cyto_Texture_Variance_Blue_5_00
cyto_Texture_Variance_Blue_5_01
cyto_Texture_Variance_Blue_5_02
cyto_Texture_Variance_Blue_5_03
cyto_num_of_cell
nuc_AreaShape_Area
nuc_AreaShape_Compactness
nuc_AreaShape_Eccentricity
nuc_AreaShape_Extent
nuc_AreaShape_FormFactor
nuc_AreaShape_MajorAxisLength
nuc_AreaShape_MaxFeretDiameter
nuc_AreaShape_MaximumRadius
nuc_AreaShape_MeanRadius
nuc_AreaShape_MedianRadius
nuc_AreaShape_MinFeretDiameter
nuc_AreaShape_MinorAxisLength
nuc_AreaShape_Perimeter
nuc_AreaShape_Solidity
nuc_Texture_AngularSecondMoment_Blue_3_00
nuc_Texture_AngularSecondMoment_Blue_3_01
nuc_Texture_AngularSecondMoment_Blue_3_02
nuc_Texture_AngularSecondMoment_Blue_3_03
nuc_Texture_AngularSecondMoment_Blue_5_00
nuc_Texture_AngularSecondMoment_Blue_5_01
nuc_Texture_AngularSecondMoment_Blue_5_02
nuc_Texture_AngularSecondMoment_Blue_5_03

nuc_Texture_Contrast_Blue_3_00
nuc_Texture_Contrast_Blue_3_01
nuc_Texture_Contrast_Blue_3_02
nuc_Texture_Contrast_Blue_3_03
nuc_Texture_Contrast_Blue_5_00
nuc_Texture_Contrast_Blue_5_01
nuc_Texture_Contrast_Blue_5_02
nuc_Texture_Contrast_Blue_5_03
nuc_Texture_Correlation_Blue_3_00
nuc_Texture_Correlation_Blue_3_01
nuc_Texture_Correlation_Blue_3_02
nuc_Texture_Correlation_Blue_3_03
nuc_Texture_Correlation_Blue_5_00
nuc_Texture_Correlation_Blue_5_01
nuc_Texture_Correlation_Blue_5_02
nuc_Texture_Correlation_Blue_5_03
nuc_Texture_DifferenceEntropy_Blue_3_00
nuc_Texture_DifferenceEntropy_Blue_3_01
nuc_Texture_DifferenceEntropy_Blue_3_02
nuc_Texture_DifferenceEntropy_Blue_3_03
nuc_Texture_DifferenceEntropy_Blue_5_00
nuc_Texture_DifferenceEntropy_Blue_5_01
nuc_Texture_DifferenceEntropy_Blue_5_02
nuc_Texture_DifferenceEntropy_Blue_5_03
nuc_Texture_DifferenceVariance_Blue_3_00
nuc_Texture_DifferenceVariance_Blue_3_01
nuc_Texture_DifferenceVariance_Blue_3_02
nuc_Texture_DifferenceVariance_Blue_3_03
nuc_Texture_DifferenceVariance_Blue_5_00
nuc_Texture_DifferenceVariance_Blue_5_01
nuc_Texture_DifferenceVariance_Blue_5_02
nuc_Texture_DifferenceVariance_Blue_5_03
nuc_Texture_Entropy_Blue_3_00
nuc_Texture_Entropy_Blue_3_01
nuc_Texture_Entropy_Blue_3_02
nuc_Texture_Entropy_Blue_3_03
nuc_Texture_Entropy_Blue_5_00
nuc_Texture_Entropy_Blue_5_01
nuc_Texture_Entropy_Blue_5_02
nuc_Texture_Entropy_Blue_5_03
nuc_Texture_InfoMeas1_Blue_3_00
nuc_Texture_InfoMeas1_Blue_3_01
nuc_Texture_InfoMeas1_Blue_3_02
nuc_Texture_InfoMeas1_Blue_3_03
nuc_Texture_InfoMeas1_Blue_5_00
nuc_Texture_InfoMeas1_Blue_5_01

nuc_Texture_InfoMeas1_Blue_5_02
nuc_Texture_InfoMeas1_Blue_5_03
nuc_Texture_InfoMeas2_Blue_3_00
nuc_Texture_InfoMeas2_Blue_3_01
nuc_Texture_InfoMeas2_Blue_3_02
nuc_Texture_InfoMeas2_Blue_3_03
nuc_Texture_InfoMeas2_Blue_5_00
nuc_Texture_InfoMeas2_Blue_5_01
nuc_Texture_InfoMeas2_Blue_5_02
nuc_Texture_InfoMeas2_Blue_5_03
nuc_Texture_InverseDifferenceMoment_Blue_3_00
nuc_Texture_InverseDifferenceMoment_Blue_3_01
nuc_Texture_InverseDifferenceMoment_Blue_3_02
nuc_Texture_InverseDifferenceMoment_Blue_3_03
nuc_Texture_InverseDifferenceMoment_Blue_5_00
nuc_Texture_InverseDifferenceMoment_Blue_5_01
nuc_Texture_InverseDifferenceMoment_Blue_5_02
nuc_Texture_InverseDifferenceMoment_Blue_5_03
nuc_Texture_SumAverage_Blue_3_00
nuc_Texture_SumAverage_Blue_3_01
nuc_Texture_SumAverage_Blue_3_02
nuc_Texture_SumAverage_Blue_3_03
nuc_Texture_SumAverage_Blue_5_00
nuc_Texture_SumAverage_Blue_5_01
nuc_Texture_SumAverage_Blue_5_02
nuc_Texture_SumAverage_Blue_5_03
nuc_Texture_SumEntropy_Blue_3_00
nuc_Texture_SumEntropy_Blue_3_01
nuc_Texture_SumEntropy_Blue_3_02
nuc_Texture_SumEntropy_Blue_3_03
nuc_Texture_SumEntropy_Blue_5_00
nuc_Texture_SumEntropy_Blue_5_01
nuc_Texture_SumEntropy_Blue_5_02
nuc_Texture_SumEntropy_Blue_5_03
nuc_Texture_SumVariance_Blue_3_00
nuc_Texture_SumVariance_Blue_3_01
nuc_Texture_SumVariance_Blue_3_02
nuc_Texture_SumVariance_Blue_3_03
nuc_Texture_SumVariance_Blue_5_00
nuc_Texture_SumVariance_Blue_5_01
nuc_Texture_SumVariance_Blue_5_02
nuc_Texture_SumVariance_Blue_5_03
nuc_Texture_Variance_Blue_3_00
nuc_Texture_Variance_Blue_3_01
nuc_Texture_Variance_Blue_3_02
nuc_Texture_Variance_Blue_3_03

nuc_Texture_Variance_Blue_5_00
nuc_Texture_Variance_Blue_5_01
nuc_Texture_Variance_Blue_5_02
nuc_Texture_Variance_Blue_5_03
nuc_num_of_cell
firstDer_ez3_cyto_AreaShape_Area
firstDer_ez3_cyto_AreaShape_Compactness
firstDer_ez3_cyto_AreaShape_Eccentricity
firstDer_ez3_cyto_AreaShape_Extent
firstDer_ez3_cyto_AreaShape_FormFactor
firstDer_ez3_cyto_AreaShape_MajorAxisLength
firstDer_ez3_cyto_AreaShape_MaxFeretDiameter
firstDer_ez3_cyto_AreaShape_MaximumRadius
firstDer_ez3_cyto_AreaShape_MeanRadius
firstDer_ez3_cyto_AreaShape_MedianRadius
firstDer_ez3_cyto_AreaShape_MinFeretDiameter
firstDer_ez3_cyto_AreaShape_MinorAxisLength
firstDer_ez3_cyto_AreaShape_Perimeter
firstDer_ez3_cyto_AreaShape_Solidity
firstDer_ez3_cyto_Texture_AngularSecondMoment_Blue_3_00
firstDer_ez3_cyto_Texture_AngularSecondMoment_Blue_3_01
firstDer_ez3_cyto_Texture_AngularSecondMoment_Blue_3_02
firstDer_ez3_cyto_Texture_AngularSecondMoment_Blue_3_03
firstDer_ez3_cyto_Texture_AngularSecondMoment_Blue_5_00
firstDer_ez3_cyto_Texture_AngularSecondMoment_Blue_5_01
firstDer_ez3_cyto_Texture_AngularSecondMoment_Blue_5_02
firstDer_ez3_cyto_Texture_AngularSecondMoment_Blue_5_03
firstDer_ez3_cyto_Texture_Contrast_Blue_3_00
firstDer_ez3_cyto_Texture_Contrast_Blue_3_01
firstDer_ez3_cyto_Texture_Contrast_Blue_3_02
firstDer_ez3_cyto_Texture_Contrast_Blue_3_03
firstDer_ez3_cyto_Texture_Contrast_Blue_5_00
firstDer_ez3_cyto_Texture_Contrast_Blue_5_01
firstDer_ez3_cyto_Texture_Contrast_Blue_5_02
firstDer_ez3_cyto_Texture_Contrast_Blue_5_03
firstDer_ez3_cyto_Texture_Correlation_Blue_3_00
firstDer_ez3_cyto_Texture_Correlation_Blue_3_01
firstDer_ez3_cyto_Texture_Correlation_Blue_3_02
firstDer_ez3_cyto_Texture_Correlation_Blue_3_03
firstDer_ez3_cyto_Texture_Correlation_Blue_5_00
firstDer_ez3_cyto_Texture_Correlation_Blue_5_01
firstDer_ez3_cyto_Texture_Correlation_Blue_5_02
firstDer_ez3_cyto_Texture_Correlation_Blue_5_03
firstDer_ez3_cyto_Texture_DifferenceEntropy_Blue_3_00
firstDer_ez3_cyto_Texture_DifferenceEntropy_Blue_3_01
firstDer_ez3_cyto_Texture_DifferenceEntropy_Blue_3_02

firstDer_ez3_cyto_Texture_DifferenceEntropy_Blue_3_03
firstDer_ez3_cyto_Texture_DifferenceEntropy_Blue_5_00
firstDer_ez3_cyto_Texture_DifferenceEntropy_Blue_5_01
firstDer_ez3_cyto_Texture_DifferenceEntropy_Blue_5_02
firstDer_ez3_cyto_Texture_DifferenceEntropy_Blue_5_03
firstDer_ez3_cyto_Texture_DifferenceVariance_Blue_3_00
firstDer_ez3_cyto_Texture_DifferenceVariance_Blue_3_01
firstDer_ez3_cyto_Texture_DifferenceVariance_Blue_3_02
firstDer_ez3_cyto_Texture_DifferenceVariance_Blue_3_03
firstDer_ez3_cyto_Texture_DifferenceVariance_Blue_5_00
firstDer_ez3_cyto_Texture_DifferenceVariance_Blue_5_01
firstDer_ez3_cyto_Texture_DifferenceVariance_Blue_5_02
firstDer_ez3_cyto_Texture_DifferenceVariance_Blue_5_03
firstDer_ez3_cyto_Texture_Entropy_Blue_3_00
firstDer_ez3_cyto_Texture_Entropy_Blue_3_01
firstDer_ez3_cyto_Texture_Entropy_Blue_3_02
firstDer_ez3_cyto_Texture_Entropy_Blue_3_03
firstDer_ez3_cyto_Texture_Entropy_Blue_5_00
firstDer_ez3_cyto_Texture_Entropy_Blue_5_01
firstDer_ez3_cyto_Texture_Entropy_Blue_5_02
firstDer_ez3_cyto_Texture_Entropy_Blue_5_03
firstDer_ez3_cyto_Texture_InfoMeas1_Blue_3_00
firstDer_ez3_cyto_Texture_InfoMeas1_Blue_3_01
firstDer_ez3_cyto_Texture_InfoMeas1_Blue_3_02
firstDer_ez3_cyto_Texture_InfoMeas1_Blue_3_03
firstDer_ez3_cyto_Texture_InfoMeas1_Blue_5_00
firstDer_ez3_cyto_Texture_InfoMeas1_Blue_5_01
firstDer_ez3_cyto_Texture_InfoMeas1_Blue_5_02
firstDer_ez3_cyto_Texture_InfoMeas1_Blue_5_03
firstDer_ez3_cyto_Texture_InfoMeas2_Blue_3_00
firstDer_ez3_cyto_Texture_InfoMeas2_Blue_3_01
firstDer_ez3_cyto_Texture_InfoMeas2_Blue_3_02
firstDer_ez3_cyto_Texture_InfoMeas2_Blue_3_03
firstDer_ez3_cyto_Texture_InfoMeas2_Blue_5_00
firstDer_ez3_cyto_Texture_InfoMeas2_Blue_5_01
firstDer_ez3_cyto_Texture_InfoMeas2_Blue_5_02
firstDer_ez3_cyto_Texture_InfoMeas2_Blue_5_03
firstDer_ez3_cyto_Texture_InverseDifferenceMoment_Blue_3_00
firstDer_ez3_cyto_Texture_InverseDifferenceMoment_Blue_3_01
firstDer_ez3_cyto_Texture_InverseDifferenceMoment_Blue_3_02
firstDer_ez3_cyto_Texture_InverseDifferenceMoment_Blue_3_03
firstDer_ez3_cyto_Texture_InverseDifferenceMoment_Blue_5_00
firstDer_ez3_cyto_Texture_InverseDifferenceMoment_Blue_5_01
firstDer_ez3_cyto_Texture_InverseDifferenceMoment_Blue_5_02
firstDer_ez3_cyto_Texture_InverseDifferenceMoment_Blue_5_03
firstDer_ez3_cyto_Texture_SumAverage_Blue_3_00

firstDer_ez3_cyto_Texture_SumAverage_Blue_3_01
firstDer_ez3_cyto_Texture_SumAverage_Blue_3_02
firstDer_ez3_cyto_Texture_SumAverage_Blue_3_03
firstDer_ez3_cyto_Texture_SumAverage_Blue_5_00
firstDer_ez3_cyto_Texture_SumAverage_Blue_5_01
firstDer_ez3_cyto_Texture_SumAverage_Blue_5_02
firstDer_ez3_cyto_Texture_SumAverage_Blue_5_03
firstDer_ez3_cyto_Texture_SumEntropy_Blue_3_00
firstDer_ez3_cyto_Texture_SumEntropy_Blue_3_01
firstDer_ez3_cyto_Texture_SumEntropy_Blue_3_02
firstDer_ez3_cyto_Texture_SumEntropy_Blue_3_03
firstDer_ez3_cyto_Texture_SumEntropy_Blue_5_00
firstDer_ez3_cyto_Texture_SumEntropy_Blue_5_01
firstDer_ez3_cyto_Texture_SumEntropy_Blue_5_02
firstDer_ez3_cyto_Texture_SumEntropy_Blue_5_03
firstDer_ez3_cyto_Texture_SumVariance_Blue_3_00
firstDer_ez3_cyto_Texture_SumVariance_Blue_3_01
firstDer_ez3_cyto_Texture_SumVariance_Blue_3_02
firstDer_ez3_cyto_Texture_SumVariance_Blue_3_03
firstDer_ez3_cyto_Texture_SumVariance_Blue_5_00
firstDer_ez3_cyto_Texture_SumVariance_Blue_5_01
firstDer_ez3_cyto_Texture_SumVariance_Blue_5_02
firstDer_ez3_cyto_Texture_SumVariance_Blue_5_03
firstDer_ez3_cyto_Texture_Variance_Blue_3_00
firstDer_ez3_cyto_Texture_Variance_Blue_3_01
firstDer_ez3_cyto_Texture_Variance_Blue_3_02
firstDer_ez3_cyto_Texture_Variance_Blue_3_03
firstDer_ez3_cyto_Texture_Variance_Blue_5_00
firstDer_ez3_cyto_Texture_Variance_Blue_5_01
firstDer_ez3_cyto_Texture_Variance_Blue_5_02
firstDer_ez3_cyto_Texture_Variance_Blue_5_03
firstDer_ez3_cyto_num_of_cell
firstDer_ez3_nuc_AreaShape_Area
firstDer_ez3_nuc_AreaShape_Compactness
firstDer_ez3_nuc_AreaShape_Eccentricity
firstDer_ez3_nuc_AreaShape_Extent
firstDer_ez3_nuc_AreaShape_FormFactor
firstDer_ez3_nuc_AreaShape_MajorAxisLength
firstDer_ez3_nuc_AreaShape_MaxFeretDiameter
firstDer_ez3_nuc_AreaShape_MaximumRadius
firstDer_ez3_nuc_AreaShape_MeanRadius
firstDer_ez3_nuc_AreaShape_MedianRadius
firstDer_ez3_nuc_AreaShape_MinFeretDiameter
firstDer_ez3_nuc_AreaShape_MinorAxisLength
firstDer_ez3_nuc_AreaShape_Perimeter
firstDer_ez3_nuc_AreaShape_Solidity

firstDer_ez3_nuc_Texture_AngularSecondMoment_Blue_3_00
firstDer_ez3_nuc_Texture_AngularSecondMoment_Blue_3_01
firstDer_ez3_nuc_Texture_AngularSecondMoment_Blue_3_02
firstDer_ez3_nuc_Texture_AngularSecondMoment_Blue_3_03
firstDer_ez3_nuc_Texture_AngularSecondMoment_Blue_5_00
firstDer_ez3_nuc_Texture_AngularSecondMoment_Blue_5_01
firstDer_ez3_nuc_Texture_AngularSecondMoment_Blue_5_02
firstDer_ez3_nuc_Texture_AngularSecondMoment_Blue_5_03
firstDer_ez3_nuc_Texture_Contrast_Blue_3_00
firstDer_ez3_nuc_Texture_Contrast_Blue_3_01
firstDer_ez3_nuc_Texture_Contrast_Blue_3_02
firstDer_ez3_nuc_Texture_Contrast_Blue_3_03
firstDer_ez3_nuc_Texture_Contrast_Blue_5_00
firstDer_ez3_nuc_Texture_Contrast_Blue_5_01
firstDer_ez3_nuc_Texture_Contrast_Blue_5_02
firstDer_ez3_nuc_Texture_Contrast_Blue_5_03
firstDer_ez3_nuc_Texture_Correlation_Blue_3_00
firstDer_ez3_nuc_Texture_Correlation_Blue_3_01
firstDer_ez3_nuc_Texture_Correlation_Blue_3_02
firstDer_ez3_nuc_Texture_Correlation_Blue_3_03
firstDer_ez3_nuc_Texture_Correlation_Blue_5_00
firstDer_ez3_nuc_Texture_Correlation_Blue_5_01
firstDer_ez3_nuc_Texture_Correlation_Blue_5_02
firstDer_ez3_nuc_Texture_Correlation_Blue_5_03
firstDer_ez3_nuc_Texture_DifferenceEntropy_Blue_3_00
firstDer_ez3_nuc_Texture_DifferenceEntropy_Blue_3_01
firstDer_ez3_nuc_Texture_DifferenceEntropy_Blue_3_02
firstDer_ez3_nuc_Texture_DifferenceEntropy_Blue_3_03
firstDer_ez3_nuc_Texture_DifferenceEntropy_Blue_5_00
firstDer_ez3_nuc_Texture_DifferenceEntropy_Blue_5_01
firstDer_ez3_nuc_Texture_DifferenceEntropy_Blue_5_02
firstDer_ez3_nuc_Texture_DifferenceEntropy_Blue_5_03
firstDer_ez3_nuc_Texture_DifferenceVariance_Blue_3_00
firstDer_ez3_nuc_Texture_DifferenceVariance_Blue_3_01
firstDer_ez3_nuc_Texture_DifferenceVariance_Blue_3_02
firstDer_ez3_nuc_Texture_DifferenceVariance_Blue_3_03
firstDer_ez3_nuc_Texture_DifferenceVariance_Blue_5_00
firstDer_ez3_nuc_Texture_DifferenceVariance_Blue_5_01
firstDer_ez3_nuc_Texture_DifferenceVariance_Blue_5_02
firstDer_ez3_nuc_Texture_DifferenceVariance_Blue_5_03
firstDer_ez3_nuc_Texture_Entropy_Blue_3_00
firstDer_ez3_nuc_Texture_Entropy_Blue_3_01
firstDer_ez3_nuc_Texture_Entropy_Blue_3_02
firstDer_ez3_nuc_Texture_Entropy_Blue_3_03
firstDer_ez3_nuc_Texture_Entropy_Blue_5_00
firstDer_ez3_nuc_Texture_Entropy_Blue_5_01

firstDer_ez3_nuc_Texture_Entropy_Blue_5_02
firstDer_ez3_nuc_Texture_Entropy_Blue_5_03
firstDer_ez3_nuc_Texture_InfoMeas1_Blue_3_00
firstDer_ez3_nuc_Texture_InfoMeas1_Blue_3_01
firstDer_ez3_nuc_Texture_InfoMeas1_Blue_3_02
firstDer_ez3_nuc_Texture_InfoMeas1_Blue_3_03
firstDer_ez3_nuc_Texture_InfoMeas1_Blue_5_00
firstDer_ez3_nuc_Texture_InfoMeas1_Blue_5_01
firstDer_ez3_nuc_Texture_InfoMeas1_Blue_5_02
firstDer_ez3_nuc_Texture_InfoMeas1_Blue_5_03
firstDer_ez3_nuc_Texture_InfoMeas2_Blue_3_00
firstDer_ez3_nuc_Texture_InfoMeas2_Blue_3_01
firstDer_ez3_nuc_Texture_InfoMeas2_Blue_3_02
firstDer_ez3_nuc_Texture_InfoMeas2_Blue_3_03
firstDer_ez3_nuc_Texture_InfoMeas2_Blue_5_00
firstDer_ez3_nuc_Texture_InfoMeas2_Blue_5_01
firstDer_ez3_nuc_Texture_InfoMeas2_Blue_5_02
firstDer_ez3_nuc_Texture_InfoMeas2_Blue_5_03
firstDer_ez3_nuc_Texture_InverseDifferenceMoment_Blue_3_00
firstDer_ez3_nuc_Texture_InverseDifferenceMoment_Blue_3_01
firstDer_ez3_nuc_Texture_InverseDifferenceMoment_Blue_3_02
firstDer_ez3_nuc_Texture_InverseDifferenceMoment_Blue_3_03
firstDer_ez3_nuc_Texture_InverseDifferenceMoment_Blue_5_00
firstDer_ez3_nuc_Texture_InverseDifferenceMoment_Blue_5_01
firstDer_ez3_nuc_Texture_InverseDifferenceMoment_Blue_5_02
firstDer_ez3_nuc_Texture_InverseDifferenceMoment_Blue_5_03
firstDer_ez3_nuc_Texture_SumAverage_Blue_3_00
firstDer_ez3_nuc_Texture_SumAverage_Blue_3_01
firstDer_ez3_nuc_Texture_SumAverage_Blue_3_02
firstDer_ez3_nuc_Texture_SumAverage_Blue_3_03
firstDer_ez3_nuc_Texture_SumAverage_Blue_5_00
firstDer_ez3_nuc_Texture_SumAverage_Blue_5_01
firstDer_ez3_nuc_Texture_SumAverage_Blue_5_02
firstDer_ez3_nuc_Texture_SumAverage_Blue_5_03
firstDer_ez3_nuc_Texture_SumEntropy_Blue_3_00
firstDer_ez3_nuc_Texture_SumEntropy_Blue_3_01
firstDer_ez3_nuc_Texture_SumEntropy_Blue_3_02
firstDer_ez3_nuc_Texture_SumEntropy_Blue_3_03
firstDer_ez3_nuc_Texture_SumEntropy_Blue_5_00
firstDer_ez3_nuc_Texture_SumEntropy_Blue_5_01
firstDer_ez3_nuc_Texture_SumEntropy_Blue_5_02
firstDer_ez3_nuc_Texture_SumEntropy_Blue_5_03
firstDer_ez3_nuc_Texture_SumVariance_Blue_3_00
firstDer_ez3_nuc_Texture_SumVariance_Blue_3_01
firstDer_ez3_nuc_Texture_SumVariance_Blue_3_02
firstDer_ez3_nuc_Texture_SumVariance_Blue_3_03

firstDer_ez3_nuc_Texture_SumVariance_Blue_5_00
firstDer_ez3_nuc_Texture_SumVariance_Blue_5_01
firstDer_ez3_nuc_Texture_SumVariance_Blue_5_02
firstDer_ez3_nuc_Texture_SumVariance_Blue_5_03
firstDer_ez3_nuc_Texture_Variance_Blue_3_00
firstDer_ez3_nuc_Texture_Variance_Blue_3_01
firstDer_ez3_nuc_Texture_Variance_Blue_3_02
firstDer_ez3_nuc_Texture_Variance_Blue_3_03
firstDer_ez3_nuc_Texture_Variance_Blue_5_00
firstDer_ez3_nuc_Texture_Variance_Blue_5_01
firstDer_ez3_nuc_Texture_Variance_Blue_5_02
firstDer_ez3_nuc_Texture_Variance_Blue_5_03
firstDer_ez3_nuc_num_of_cell
firstDer_ez7_cyto_AreaShape_Area
firstDer_ez7_cyto_AreaShape_Compactness
firstDer_ez7_cyto_AreaShape_Eccentricity
firstDer_ez7_cyto_AreaShape_Extent
firstDer_ez7_cyto_AreaShape_FormFactor
firstDer_ez7_cyto_AreaShape_MajorAxisLength
firstDer_ez7_cyto_AreaShape_MaxFeretDiameter
firstDer_ez7_cyto_AreaShape_MaximumRadius
firstDer_ez7_cyto_AreaShape_MeanRadius
firstDer_ez7_cyto_AreaShape_MedianRadius
firstDer_ez7_cyto_AreaShape_MinFeretDiameter
firstDer_ez7_cyto_AreaShape_MinorAxisLength
firstDer_ez7_cyto_AreaShape_Perimeter
firstDer_ez7_cyto_AreaShape_Solidity
firstDer_ez7_cyto_Texture_AngularSecondMoment_Blue_3_00
firstDer_ez7_cyto_Texture_AngularSecondMoment_Blue_3_01
firstDer_ez7_cyto_Texture_AngularSecondMoment_Blue_3_02
firstDer_ez7_cyto_Texture_AngularSecondMoment_Blue_3_03
firstDer_ez7_cyto_Texture_AngularSecondMoment_Blue_5_00
firstDer_ez7_cyto_Texture_AngularSecondMoment_Blue_5_01
firstDer_ez7_cyto_Texture_AngularSecondMoment_Blue_5_02
firstDer_ez7_cyto_Texture_AngularSecondMoment_Blue_5_03
firstDer_ez7_cyto_Texture_Contrast_Blue_3_00
firstDer_ez7_cyto_Texture_Contrast_Blue_3_01
firstDer_ez7_cyto_Texture_Contrast_Blue_3_02
firstDer_ez7_cyto_Texture_Contrast_Blue_3_03
firstDer_ez7_cyto_Texture_Contrast_Blue_5_00
firstDer_ez7_cyto_Texture_Contrast_Blue_5_01
firstDer_ez7_cyto_Texture_Contrast_Blue_5_02
firstDer_ez7_cyto_Texture_Contrast_Blue_5_03
firstDer_ez7_cyto_Texture_Correlation_Blue_3_00
firstDer_ez7_cyto_Texture_Correlation_Blue_3_01
firstDer_ez7_cyto_Texture_Correlation_Blue_3_02

firstDer_ez7_cyto_Texture_Correlation_Blue_3_03
firstDer_ez7_cyto_Texture_Correlation_Blue_5_00
firstDer_ez7_cyto_Texture_Correlation_Blue_5_01
firstDer_ez7_cyto_Texture_Correlation_Blue_5_02
firstDer_ez7_cyto_Texture_Correlation_Blue_5_03
firstDer_ez7_cyto_Texture_DifferenceEntropy_Blue_3_00
firstDer_ez7_cyto_Texture_DifferenceEntropy_Blue_3_01
firstDer_ez7_cyto_Texture_DifferenceEntropy_Blue_3_02
firstDer_ez7_cyto_Texture_DifferenceEntropy_Blue_3_03
firstDer_ez7_cyto_Texture_DifferenceEntropy_Blue_5_00
firstDer_ez7_cyto_Texture_DifferenceEntropy_Blue_5_01
firstDer_ez7_cyto_Texture_DifferenceEntropy_Blue_5_02
firstDer_ez7_cyto_Texture_DifferenceEntropy_Blue_5_03
firstDer_ez7_cyto_Texture_DifferenceVariance_Blue_3_00
firstDer_ez7_cyto_Texture_DifferenceVariance_Blue_3_01
firstDer_ez7_cyto_Texture_DifferenceVariance_Blue_3_02
firstDer_ez7_cyto_Texture_DifferenceVariance_Blue_3_03
firstDer_ez7_cyto_Texture_DifferenceVariance_Blue_5_00
firstDer_ez7_cyto_Texture_DifferenceVariance_Blue_5_01
firstDer_ez7_cyto_Texture_DifferenceVariance_Blue_5_02
firstDer_ez7_cyto_Texture_DifferenceVariance_Blue_5_03
firstDer_ez7_cyto_Texture_Entropy_Blue_3_00
firstDer_ez7_cyto_Texture_Entropy_Blue_3_01
firstDer_ez7_cyto_Texture_Entropy_Blue_3_02
firstDer_ez7_cyto_Texture_Entropy_Blue_3_03
firstDer_ez7_cyto_Texture_Entropy_Blue_5_00
firstDer_ez7_cyto_Texture_Entropy_Blue_5_01
firstDer_ez7_cyto_Texture_Entropy_Blue_5_02
firstDer_ez7_cyto_Texture_Entropy_Blue_5_03
firstDer_ez7_cyto_Texture_InfoMeas1_Blue_3_00
firstDer_ez7_cyto_Texture_InfoMeas1_Blue_3_01
firstDer_ez7_cyto_Texture_InfoMeas1_Blue_3_02
firstDer_ez7_cyto_Texture_InfoMeas1_Blue_3_03
firstDer_ez7_cyto_Texture_InfoMeas1_Blue_5_00
firstDer_ez7_cyto_Texture_InfoMeas1_Blue_5_01
firstDer_ez7_cyto_Texture_InfoMeas1_Blue_5_02
firstDer_ez7_cyto_Texture_InfoMeas1_Blue_5_03
firstDer_ez7_cyto_Texture_InfoMeas2_Blue_3_00
firstDer_ez7_cyto_Texture_InfoMeas2_Blue_3_01
firstDer_ez7_cyto_Texture_InfoMeas2_Blue_3_02
firstDer_ez7_cyto_Texture_InfoMeas2_Blue_3_03
firstDer_ez7_cyto_Texture_InfoMeas2_Blue_5_00
firstDer_ez7_cyto_Texture_InfoMeas2_Blue_5_01
firstDer_ez7_cyto_Texture_InfoMeas2_Blue_5_02
firstDer_ez7_cyto_Texture_InfoMeas2_Blue_5_03
firstDer_ez7_cyto_Texture_InverseDifferenceMoment_Blue_3_00

firstDer_ez7_cyto_Texture_InverseDifferenceMoment_Blue_3_01
firstDer_ez7_cyto_Texture_InverseDifferenceMoment_Blue_3_02
firstDer_ez7_cyto_Texture_InverseDifferenceMoment_Blue_3_03
firstDer_ez7_cyto_Texture_InverseDifferenceMoment_Blue_5_00
firstDer_ez7_cyto_Texture_InverseDifferenceMoment_Blue_5_01
firstDer_ez7_cyto_Texture_InverseDifferenceMoment_Blue_5_02
firstDer_ez7_cyto_Texture_InverseDifferenceMoment_Blue_5_03
firstDer_ez7_cyto_Texture_SumAverage_Blue_3_00
firstDer_ez7_cyto_Texture_SumAverage_Blue_3_01
firstDer_ez7_cyto_Texture_SumAverage_Blue_3_02
firstDer_ez7_cyto_Texture_SumAverage_Blue_3_03
firstDer_ez7_cyto_Texture_SumAverage_Blue_5_00
firstDer_ez7_cyto_Texture_SumAverage_Blue_5_01
firstDer_ez7_cyto_Texture_SumAverage_Blue_5_02
firstDer_ez7_cyto_Texture_SumAverage_Blue_5_03
firstDer_ez7_cyto_Texture_SumEntropy_Blue_3_00
firstDer_ez7_cyto_Texture_SumEntropy_Blue_3_01
firstDer_ez7_cyto_Texture_SumEntropy_Blue_3_02
firstDer_ez7_cyto_Texture_SumEntropy_Blue_3_03
firstDer_ez7_cyto_Texture_SumEntropy_Blue_5_00
firstDer_ez7_cyto_Texture_SumEntropy_Blue_5_01
firstDer_ez7_cyto_Texture_SumEntropy_Blue_5_02
firstDer_ez7_cyto_Texture_SumEntropy_Blue_5_03
firstDer_ez7_cyto_Texture_SumVariance_Blue_3_00
firstDer_ez7_cyto_Texture_SumVariance_Blue_3_01
firstDer_ez7_cyto_Texture_SumVariance_Blue_3_02
firstDer_ez7_cyto_Texture_SumVariance_Blue_3_03
firstDer_ez7_cyto_Texture_SumVariance_Blue_5_00
firstDer_ez7_cyto_Texture_SumVariance_Blue_5_01
firstDer_ez7_cyto_Texture_SumVariance_Blue_5_02
firstDer_ez7_cyto_Texture_SumVariance_Blue_5_03
firstDer_ez7_cyto_Texture_Variance_Blue_3_00
firstDer_ez7_cyto_Texture_Variance_Blue_3_01
firstDer_ez7_cyto_Texture_Variance_Blue_3_02
firstDer_ez7_cyto_Texture_Variance_Blue_3_03
firstDer_ez7_cyto_Texture_Variance_Blue_5_00
firstDer_ez7_cyto_Texture_Variance_Blue_5_01
firstDer_ez7_cyto_Texture_Variance_Blue_5_02
firstDer_ez7_cyto_Texture_Variance_Blue_5_03
firstDer_ez7_cyto_num_of_cell
firstDer_ez7_nuc_AreaShape_Area
firstDer_ez7_nuc_AreaShape_Compactness
firstDer_ez7_nuc_AreaShape_Eccentricity
firstDer_ez7_nuc_AreaShape_Extent
firstDer_ez7_nuc_AreaShape_FormFactor
firstDer_ez7_nuc_AreaShape_MajorAxisLength

firstDer_ez7_nuc_AreaShape_MaxFeretDiameter
firstDer_ez7_nuc_AreaShape_MaximumRadius
firstDer_ez7_nuc_AreaShape_MeanRadius
firstDer_ez7_nuc_AreaShape_MedianRadius
firstDer_ez7_nuc_AreaShape_MinFeretDiameter
firstDer_ez7_nuc_AreaShape_MinorAxisLength
firstDer_ez7_nuc_AreaShape_Perimeter
firstDer_ez7_nuc_AreaShape_Solidity
firstDer_ez7_nuc_Texture_AngularSecondMoment_Blue_3_00
firstDer_ez7_nuc_Texture_AngularSecondMoment_Blue_3_01
firstDer_ez7_nuc_Texture_AngularSecondMoment_Blue_3_02
firstDer_ez7_nuc_Texture_AngularSecondMoment_Blue_3_03
firstDer_ez7_nuc_Texture_AngularSecondMoment_Blue_5_00
firstDer_ez7_nuc_Texture_AngularSecondMoment_Blue_5_01
firstDer_ez7_nuc_Texture_AngularSecondMoment_Blue_5_02
firstDer_ez7_nuc_Texture_AngularSecondMoment_Blue_5_03
firstDer_ez7_nuc_Texture_Contrast_Blue_3_00
firstDer_ez7_nuc_Texture_Contrast_Blue_3_01
firstDer_ez7_nuc_Texture_Contrast_Blue_3_02
firstDer_ez7_nuc_Texture_Contrast_Blue_3_03
firstDer_ez7_nuc_Texture_Contrast_Blue_5_00
firstDer_ez7_nuc_Texture_Contrast_Blue_5_01
firstDer_ez7_nuc_Texture_Contrast_Blue_5_02
firstDer_ez7_nuc_Texture_Contrast_Blue_5_03
firstDer_ez7_nuc_Texture_Correlation_Blue_3_00
firstDer_ez7_nuc_Texture_Correlation_Blue_3_01
firstDer_ez7_nuc_Texture_Correlation_Blue_3_02
firstDer_ez7_nuc_Texture_Correlation_Blue_3_03
firstDer_ez7_nuc_Texture_Correlation_Blue_5_00
firstDer_ez7_nuc_Texture_Correlation_Blue_5_01
firstDer_ez7_nuc_Texture_Correlation_Blue_5_02
firstDer_ez7_nuc_Texture_Correlation_Blue_5_03
firstDer_ez7_nuc_Texture_DifferenceEntropy_Blue_3_00
firstDer_ez7_nuc_Texture_DifferenceEntropy_Blue_3_01
firstDer_ez7_nuc_Texture_DifferenceEntropy_Blue_3_02
firstDer_ez7_nuc_Texture_DifferenceEntropy_Blue_3_03
firstDer_ez7_nuc_Texture_DifferenceEntropy_Blue_5_00
firstDer_ez7_nuc_Texture_DifferenceEntropy_Blue_5_01
firstDer_ez7_nuc_Texture_DifferenceEntropy_Blue_5_02
firstDer_ez7_nuc_Texture_DifferenceEntropy_Blue_5_03
firstDer_ez7_nuc_Texture_DifferenceVariance_Blue_3_00
firstDer_ez7_nuc_Texture_DifferenceVariance_Blue_3_01
firstDer_ez7_nuc_Texture_DifferenceVariance_Blue_3_02
firstDer_ez7_nuc_Texture_DifferenceVariance_Blue_3_03
firstDer_ez7_nuc_Texture_DifferenceVariance_Blue_5_00
firstDer_ez7_nuc_Texture_DifferenceVariance_Blue_5_01

firstDer_ez7_nuc_Texture_DifferenceVariance_Blue_5_02
firstDer_ez7_nuc_Texture_DifferenceVariance_Blue_5_03
firstDer_ez7_nuc_Texture_Entropy_Blue_3_00
firstDer_ez7_nuc_Texture_Entropy_Blue_3_01
firstDer_ez7_nuc_Texture_Entropy_Blue_3_02
firstDer_ez7_nuc_Texture_Entropy_Blue_3_03
firstDer_ez7_nuc_Texture_Entropy_Blue_5_00
firstDer_ez7_nuc_Texture_Entropy_Blue_5_01
firstDer_ez7_nuc_Texture_Entropy_Blue_5_02
firstDer_ez7_nuc_Texture_Entropy_Blue_5_03
firstDer_ez7_nuc_Texture_InfoMeas1_Blue_3_00
firstDer_ez7_nuc_Texture_InfoMeas1_Blue_3_01
firstDer_ez7_nuc_Texture_InfoMeas1_Blue_3_02
firstDer_ez7_nuc_Texture_InfoMeas1_Blue_3_03
firstDer_ez7_nuc_Texture_InfoMeas1_Blue_5_00
firstDer_ez7_nuc_Texture_InfoMeas1_Blue_5_01
firstDer_ez7_nuc_Texture_InfoMeas1_Blue_5_02
firstDer_ez7_nuc_Texture_InfoMeas1_Blue_5_03
firstDer_ez7_nuc_Texture_InfoMeas2_Blue_3_00
firstDer_ez7_nuc_Texture_InfoMeas2_Blue_3_01
firstDer_ez7_nuc_Texture_InfoMeas2_Blue_3_02
firstDer_ez7_nuc_Texture_InfoMeas2_Blue_3_03
firstDer_ez7_nuc_Texture_InfoMeas2_Blue_5_00
firstDer_ez7_nuc_Texture_InfoMeas2_Blue_5_01
firstDer_ez7_nuc_Texture_InfoMeas2_Blue_5_02
firstDer_ez7_nuc_Texture_InfoMeas2_Blue_5_03
firstDer_ez7_nuc_Texture_InverseDifferenceMoment_Blue_3_00
firstDer_ez7_nuc_Texture_InverseDifferenceMoment_Blue_3_01
firstDer_ez7_nuc_Texture_InverseDifferenceMoment_Blue_3_02
firstDer_ez7_nuc_Texture_InverseDifferenceMoment_Blue_3_03
firstDer_ez7_nuc_Texture_InverseDifferenceMoment_Blue_5_00
firstDer_ez7_nuc_Texture_InverseDifferenceMoment_Blue_5_01
firstDer_ez7_nuc_Texture_InverseDifferenceMoment_Blue_5_02
firstDer_ez7_nuc_Texture_InverseDifferenceMoment_Blue_5_03
firstDer_ez7_nuc_Texture_SumAverage_Blue_3_00
firstDer_ez7_nuc_Texture_SumAverage_Blue_3_01
firstDer_ez7_nuc_Texture_SumAverage_Blue_3_02
firstDer_ez7_nuc_Texture_SumAverage_Blue_3_03
firstDer_ez7_nuc_Texture_SumAverage_Blue_5_00
firstDer_ez7_nuc_Texture_SumAverage_Blue_5_01
firstDer_ez7_nuc_Texture_SumAverage_Blue_5_02
firstDer_ez7_nuc_Texture_SumAverage_Blue_5_03
firstDer_ez7_nuc_Texture_SumEntropy_Blue_3_00
firstDer_ez7_nuc_Texture_SumEntropy_Blue_3_01
firstDer_ez7_nuc_Texture_SumEntropy_Blue_3_02
firstDer_ez7_nuc_Texture_SumEntropy_Blue_3_03

firstDer_ez7_nuc_Texture_SumEntropy_Blue_5_00
firstDer_ez7_nuc_Texture_SumEntropy_Blue_5_01
firstDer_ez7_nuc_Texture_SumEntropy_Blue_5_02
firstDer_ez7_nuc_Texture_SumEntropy_Blue_5_03
firstDer_ez7_nuc_Texture_SumVariance_Blue_3_00
firstDer_ez7_nuc_Texture_SumVariance_Blue_3_01
firstDer_ez7_nuc_Texture_SumVariance_Blue_3_02
firstDer_ez7_nuc_Texture_SumVariance_Blue_3_03
firstDer_ez7_nuc_Texture_SumVariance_Blue_5_00
firstDer_ez7_nuc_Texture_SumVariance_Blue_5_01
firstDer_ez7_nuc_Texture_SumVariance_Blue_5_02
firstDer_ez7_nuc_Texture_SumVariance_Blue_5_03
firstDer_ez7_nuc_Texture_Variance_Blue_3_00
firstDer_ez7_nuc_Texture_Variance_Blue_3_01
firstDer_ez7_nuc_Texture_Variance_Blue_3_02
firstDer_ez7_nuc_Texture_Variance_Blue_3_03
firstDer_ez7_nuc_Texture_Variance_Blue_5_00
firstDer_ez7_nuc_Texture_Variance_Blue_5_01
firstDer_ez7_nuc_Texture_Variance_Blue_5_02
firstDer_ez7_nuc_Texture_Variance_Blue_5_03
firstDer_ez7_nuc_num_of_cell

**4) Blood cell differentiation extracted features:**

Area_AreaShape_1pxM1m
Eccentricity_AreaShape_1pxM1m
Solidity_AreaShape_1pxM1m
Extent_AreaShape_1pxM1m
EulerNumber_AreaShape_1pxM1m
Perimeter_AreaShape_1pxM1m
FormFactor_AreaShape_1pxM1m
MajorAxisLength_AreaShape_1pxM1m
MinorAxisLength_AreaShape_1pxM1m
Orientation_AreaShape_1pxM1m
IntegratedIntensity_IntensityGray_ch2::OrigBlue
MeanIntensity_IntensityGray_ch2::OrigBlue
StdIntensity_IntensityGray_ch2::OrigBlue
MinIntensity_IntensityGray_ch2::OrigBlue
MaxIntensity_IntensityGray_ch2::OrigBlue
IntegratedIntensityEdge_IntensityGray_ch2::OrigBlue
MeanIntensityEdge_IntensityGray_ch2::OrigBlue
StdIntensityEdge_IntensityGray_ch2::OrigBlue
MinIntensityEdge_IntensityGray_ch2::OrigBlue
MaxIntensityEdge_IntensityGray_ch2::OrigBlue
MassDisplacement_IntensityGray_ch2::OrigBlue
IntegratedIntensity_IntensityGray_ch3::OrigRed

MeanIntensity_IntensityGray_ch3::OrigRed
StdIntensity_IntensityGray_ch3::OrigRed
MinIntensity_IntensityGray_ch3::OrigRed
MaxIntensity_IntensityGray_ch3::OrigRed
IntegratedIntensityEdge_IntensityGray_ch3::OrigRed
MeanIntensityEdge_IntensityGray_ch3::OrigRed
StdIntensityEdge_IntensityGray_ch3::OrigRed
MinIntensityEdge_IntensityGray_ch3::OrigRed
MaxIntensityEdge_IntensityGray_ch3::OrigRed
MassDisplacement_IntensityGray_ch3::OrigRed
AngularSecondMoment_TextureGray_ch2::OrigBlue_3
Contrast_TextureGray_ch2::OrigBlue_3
Correlation_TextureGray_ch2::OrigBlue_3
Variance_TextureGray_ch2::OrigBlue_3
InverseDifferenceMoment_TextureGray_ch2::OrigBlue_3
SumAverage_TextureGray_ch2::OrigBlue_3
SumVariance_TextureGray_ch2::OrigBlue_3
SumEntropy_TextureGray_ch2::OrigBlue_3
Entropy_TextureGray_ch2::OrigBlue_3
DifferenceVariance_TextureGray_ch2::OrigBlue_3
DifferenceEntropy_TextureGray_ch2::OrigBlue_3
InfoMeas1_TextureGray_ch2::OrigBlue_3
InfoMeas2_TextureGray_ch2::OrigBlue_3
GaborX_TextureGray_ch2::OrigBlue_3
GaborY_TextureGray_ch2::OrigBlue_3
AngularSecondMoment_TextureGray_ch3::OrigRed_3
Contrast_TextureGray_ch3::OrigRed_3
Correlation_TextureGray_ch3::OrigRed_3
Variance_TextureGray_ch3::OrigRed_3
InverseDifferenceMoment_TextureGray_ch3::OrigRed_3
SumAverage_TextureGray_ch3::OrigRed_3
SumVariance_TextureGray_ch3::OrigRed_3
SumEntropy_TextureGray_ch3::OrigRed_3
Entropy_TextureGray_ch3::OrigRed_3
DifferenceVariance_TextureGray_ch3::OrigRed_3
DifferenceEntropy_TextureGray_ch3::OrigRed_3
InfoMeas1_TextureGray_ch3::OrigRed_3
InfoMeas2_TextureGray_ch3::OrigRed_3
GaborX_TextureGray_ch3::OrigRed_3
GaborY_TextureGray_ch3::OrigRed_3

# Supplementary Note 3.

# Hyperparameter initialization for GP

The Gaussian Processes (GP) framework by Rasmussen & Nickisch [1] that we adapted for our regression plane provides several mean and covariance functions. We included the following functions and initialized their hyperparameters as given in the tables below.

**Notations:**

- $D_{out}$ : the output dimension (in regression plane this is 2)
- $D_{in}$ : the input dimension, i.e. the number of features
- $y_i$: the training output values in the $i$th output dimension in a column
- $rc_i$: regression plane center in the $i$th dimension
- $N$: number of training samples
- $X$: The input matrix, columns are the observations, size is $D_{in}$x$N$
- $\bar{a}$: the mean of all values in vector $a$

## I Mean functions

| Function name | Description | # parameters | Initialization |
|---|---|---|---|
| `meanNN` | Nearest neighbour mean function | 0 | |
| `meanConst` | A constant mean function | $D_{out}$ | $[rc_1, \ldots, rc_{D_{out}}]$ |
| `meanLinear` | A linear function of the inputs | $D_{in} * D_{out}$ | $(XX^T)^{-1}Xy_i, \forall i \in \{1, \ldots, D_{out}\}$ |

## II Covariance functions

Note: The covariance initializations are done separately in each output dimension, the actual number of parameters is $D_{out}$ times the number indicated below.

| Function name | Description | # parameters | Initialization |
|---|---|---|---|
| `covSEiso` | Squared Exponential with isotropic distance | 2 | $[\overline{ic}, \log(\sqrt{V_i})]$ |
| `covNNone` | Neural network covariance with single distance parameter. | 2 | Same as `covSEiso` |
| `covSEard` | Squared exponential with automatic relevance determination | $D_{in} + 1$ | $[ic, \log(\sqrt{V_i})]$ |

**Notations:**

- $ic$: initial estimated variation locally in the target space for each input variable. This is a vector with length $D_{in}$ and calculated as follows:

$$ic_i = \sum_{j=1}^{N} \frac{\overline{\text{KNN}(i,j,10)}}{N}, \forall i \in \{1,\ldots,D_{in}\}$$

  , where $\text{KNN}(i,j,k)$ stands for the set of the distances in the $i$th dimension of the $k$ closest object from the $j$th object where closeness is measured by traditional Euclidean distance in the target space.

- $V$: is the expected variance assuming uniform distribution between the bounds of the regression plane. Formally that is $\frac{(ub_i - lb_i)^2}{12}$, where $ub_i$ and $lb_i$ stand for the upper and lower bounds of the regression plane's $i$th dimension respectively.

# References

[1] Carl Edward Rasmussen and Hannes Nickisch. Gaussian processes for machine learning (gpml) toolbox. *Journal of machine learning research*, 11(Nov):3011–3015, 2010.

## Supplementary Note 4.

## Available regression models and instructions to extend the set

### I. Available regression models

Most of the models are bridged directly from available Machine Learning libraries including Weka and Mulan.

| Model | Module name in ACC | Origin | Multi-target model | Provides uncertainty |
|---|---|---|---|---|
| Gaussian Processes | WekaGP | Weka | ✗ | ✗ |
| K-Nearest Neighbours | WekaKNN | Weka | ✗ | ✗ |
| Linear Regression | WekaLinearRegression | Weka | ✗ | ✗ |
| Multi-Layer Perceptron | WekaMLP | Weka | ✗ | ✗ |
| Random Forest | WekaRandomForest | Weka | ✗ | ✗ |
| Fast Decision Tree | WekaREPTree | Weka | ✗ | ✗ |
| Support Vector Machine for Regression | WekaSMOreg | Weka | ✗ | ✗ |
| Neural Network | NNPredictor | Matlab | ✓ | ✗ |
| Gaussian Processes for ML | GPMLPredictor | Rasmussen & Nickish | ✗ | ✓ |
| Multi-target stacking | MulanMTStack | Mulan | ✓ | ✗ |
| Ensemble of Chains | MulanEnsembleOfChains | Mulan | ✓ | ✗ |

### II. Instructions to implement new models

*NOTE:* To be able to implement a new regression module you need to run ACC from the Matlab source code. The last stable version is available at www.cellclassifier.org, the latest developer version can be freely cloned via git with the following command:

```
git clone --recurse-submodules https://bitbucket.org/biomag/advanced-cell-
classifier.git
```

#### A) Regression models

The Regression Plane (RP) module of ACC uses Object-Oriented Programming to provide interface between regression models (predictors) and the core of the module. Any novel regression model can be easily integrated to the software by the following procedure:

1. Create a class in Matlab that implements the abstract `Predictor` class located in the ACC/Utils/ActiveRegression/Model folder.

2. Create a default constructor (no parameters) for your fresh class
3. Implement ALL the abstract methods from the `Predictor` class specifically for your module. `Predictor` class has extensive documentation to help the developer to get on with the process, and you may check any previously implemented model as well (they are in the ACC/Utils/ActiveRegression/Model/Predictors folder)
4. Save the new class next to the other implementations (in the ACC\Utils\ActiveRegression\Model\Predictors folder)

<u>User-parameters for your model:</u>

In case you wish to define user-tunable parameters for your module (for instance you wish to ask the parameter 'k' for K-Nearest Neighbour classifier from the user), you may easily do this via overloading the following methods of the `Predictor` parent class:

1) `getParameters` (static class function)
   Use this static function to define how many and what types of parameters your module has.
2) `setParameters` (member function)
   This is the member function which is called after the construction of your predictor class to fill out its parameters with the correct values. It receives a cellarray matching the format you specified with the `getParameters` function.
3) `getParameterValues` (member function)
   This member function should return the actual parameter settings of the module in the exact same format as the `setParameters` function gets it. It is called before cross validation so that a clean predictor can be initialized in each different batch with the exact same parameters as the original method.

For the exact formats of the input and output of these functions please check the `generateUIControls` and `fetchUIControlValues` functions in the following folder: ACC/Utils/ActiveRegression/Utils/matlab_settings_gui or at https://github.com/szkabel/matlab_settings_gui .

## B) <u>Active regression techniques</u>

Similarly to Regression Models, Active Learning is also implemented via Object-Oriented Programming (this holds for both Regression and Classification active learning algorithms). If you wish to extend ACC with your own Active Regression/Classification method you should do the following steps:

1. Create a class that implements the abstract `ALalgorithm` class (for Regression found in: ACC/Utils/ActiveRegression/Model) or `ActiveLearner` (for Classification found in: ACC/Utils/ActiveLearning).
2. Create a constructor that takes one single parameter, the cellarray of values of the user-tunable parameters as defined in the `getParameters` static function.

3. Implement ALL abstract methods, following the documentation of the class. You may check any existing implementation which are located in: ACC\Utils\ActiveRegression\Model\ALalgorithms (regression) and ACC\Utils\ActiveLearning\Implementations (classification)
4. Save your class in the appropriate folder (next to the other implementations, their location is in the previous point)

User-parameters for your active learner:

The functions for user-tunable parameters are all abstract in case of Active Learners, so an implementation must be provided. If you do not need user-parameters then safely return empty cellarrays (e.g. created by `cell(0)`). Otherwise please follow the instructions given in the documentation of `generateUIControls` and `fetchUIControlValues` functions in the following folder: ACC/Utils/ActiveRegression/Utils/matlab_settings_gui or at https://github.com/szkabel/matlab_settings_gui .
