# Supplementary material for: Regression plane concept for analysing continuous cellular processes with machine learning
Source: Nat Commun. 2021 May 5;12:2532. doi: 10.1038/s41467-021-22866-x (PMC8100172; doi:10.1038/s41467-021-22866-x)
Supplement: Supplementary file 3 — Description of Additional Supplementary Files [file 41467_2021_22866_MOESM3_ESM.pdf]

Description of Additional Supplementary Files

File Name: Supplementary Data 1
Description: Plate layout reporting the targeted genes of lipid droplet screen.

File Name: Supplementary Data 2
Description: Training set (in ACC project format) for the MitoCheck analysis, containing the trained 585 cells.

File Name: Supplementary Movie 1
Description: Video tutorial: "*Regression Plane: Annotation Possibilitie*s".

File Name: Supplementary Movie 2
Description: Video tutorial: "*Regression Plane: Output Possibilities*".

File Name: Supplementary Movie 3-8
Description: The supplementary movies are dynamically visualizing the regression plane analysis of live-cell experiments. The rectangle in the videos represents the regression plane itself and the trajectories are derived from the live-cells' *predicted positions* on the plane. Each individual trajectory is assigned to a single-cell and the animation shows how the cells traverse on the regression plane as the live-cell screening progresses (the path between the actual frames were linearly interpolated).

File Name: Supplementary Movie 3
Description: Drosophila Plasmatocyte Differentiating into Type II Lamellocyte

File Name: Supplementary Movie 4
Description: Drosophila Plasmatocyte Differentiating into Type I Lamellocyte

File Name: Supplementary Movie 5
Description: Drosophila Plasmatocyte Differentiation, dynamic visualization of trajectories using protein expression. Trajectories are coloured dynamically, visualizing changes in 2 selected cell features.
Head-colour: integrated intensity value of eaterGFP representing its expression level.
Tail-colour: integrated intensity value of MSNF9MOmCherry representing its expression level.
Expression of eaterGFP was observed in a fraction of both type I and type II lamellocytes, however, type II lamellocytes express GFP more frequently and at a higher level.

File Name: Supplementary Movie 6
Description: Drosophila Plasmatocyte Differentiation, differentiation speed. Trajectories are coloured dynamically visualizing the speed of the cells on the Regression Plane. According to the defined training strategy, this reflects the speed of differentiation. Colours are ranging from blue (slow) to red (fast). Following immune induction, type II lamellocytes start differentiation later than type I lamellocytes, however type II lamellocytes differentiate faster and in a continuous manner.

File Name: Supplementary Movie 7
Description: Dynamic Visualization of Mitosis. Trajectories show how the cells are traversing on the Regression Plane. The highlighted 4 cells are reported in detail in further supplementary movies.

File Name: Supplementary Movie 8
Description: A montage of single-cell videos of the highlighted cells in Supplementary Movie 5.

File Name: Supplementary Software 1
Description: Regression Plane user manual.

File Name: Supplementary Software 2
Description: Customized version of SIMCEP, distributed as MATLAB source code under the GNU General Public License version 3.

File Name: Supplementary Software 3
Description: MATLAB script provided to the 5 microscopists analysing the synthetic dataset using standard classification approaches.
