# Supplementary material for: Regression plane concept for analysing continuous cellular processes with machine learning
Source: Nat Commun. 2021 May 5;12:2532. doi: 10.1038/s41467-021-22866-x (PMC8100172; doi:10.1038/s41467-021-22866-x)
Supplement: Supplementary file 14 — Supplementary Software 1 [file 41467_2021_22866_MOESM14_ESM.pdf]

Advanced Cell Classifier (ACC) – user manual

www.cellclassifier.org

REGRESSION PLANE

July 2020

Prof. Peter Horvath, PhD
Synthetic and Systems Biology Unit
Biological Research Centre (BRC)
Szeged, Hungary
horvath.peter@brc.hu

INDEX

1	**Brief description**	p.	4
2	**License**	p.	4
	2.1 Third-party tools	.	.
3	**System requirements**	p.	5
4	**Installation**	p.	5
5	**Getting started with the Regression Plane**	p.	6
6	**Regression Plane Graphical User Interface**	p.	9
	6.1 Main view	.	.
	6.2 Visualization options	.	.
	6.3 Annotation options	.	.
	6.4 Toolbar buttons	.	.
7	**Annotation with the Regression Plane**	p.	12
	7.1 Manual annotation	.	.
	7.2 Guided annotation	.	.
	7.3 Active regression	.	.
8	**Output of the Regression Plane**	p.	19
	8.1 Cell-by-cell prediction	.	.
	8.2 Predict images	.	.
	8.3 Predict plates	.	.
	8.4 Well-based analysis of treatments	.	.
	8.5 Trajectory Plot	.	.
9	**Additional information**	p.	45

LIST OF FIGURES

1	Main GUI of ACC (when a dataset is loaded)	p. 6
2	GUI for creating a regression class	p. 7
3	List of available regressors	p. 8
4	Regression Plane GUI	p. 9
5	Regression Plane with a selected cell	p. 10
6	Regression Plane with a block of selected cell	p. 11
7	Toolbar buttons of the Regression Plane	p. 11
8	Icon of a regression class	p. 13
9	Main window of the Color Frame module	p. 14
10	Color Frame module: example of visualisation	p. 15
11	Image selector of the Predict Image GUI	p. 16
12	Prediction plane with the "Close prediction" button	p. 16
13	Active regression settings GUI	p. 18
14	Path for changing the active regression settings	p. 18
15	Cell-by-cell prediction	p. 20
16	Error vectors	p. 20
17	GUI: predict plate settings	p. 21
18	Heatmap of a single well (shown at different resolutions)	p. 22
19	Unsupervised learning methods for data discovery	p. 23
20	Regression report	p. 24
21	Plot of plots: heatmaps	p. 26
22	Distance-based clustergram	p. 27
23	Output folder containing the "temp" folder	p. 28
24	Meta-visualization of the heatmaps	p. 29
25	Trajectory Plot GUI with main sections	p. 30
26	Trajectory Plot toolbar buttons	p. 30
27	Trajectory Plot GUI, main window with groups of trajectories	p. 31
28	Trajectory Plot GUI with trajectories manually selected	p. 34
29	Trajectory Plot module: main window of the Slider Selector.	p. 36
30	Trajectory Plot module: Slider Selector Property list	p. 36
31	Trajectory Plot module: list of possible measurements.	p. 37
32	Trajectory Plot GUI, Line Property window	p. 38
33	Trajectory Plot module: feature list	p. 40
34	Line with thumbnails of the cell	p. 40
35	Trajectory Plot, column overviews of the input “.csv” file	p. 41
36	Trajectory Plot – Input file. Example	p. 42
37	Trajectory information generation	p. 43
38	Trajectory generator wizard: name analysis	p. 43
39	Trajectory generator wizard: cell tracking	p. 44
40	ACC logo	p. 45

1. BRIEF DESCRIPTION

Advanced Cell Classifier (ACC) is a user friendly, data visualization and analyser software tool for cell-based high-content screens and tissue section images. The main aim of ACC is to provide accurate phenotypic analysis using advanced machine learning methods with minimal user interaction.

One of the main functionalities of ACC is to classify cells into separable phenotypic classes. However, most of the biological processes are inherently continuous (*e.g*. cell division, uptake of a chemical compound that initiates a new cell state). By discretizing these continuous stages, the analysis can be significantly restricted. The *Regression Plane* gives an opportunity to analyse cells in a continuous way, without the need of defining discrete stages.

This document is a short help tutorial to describe the main functions of the *Regression Plane*. It is written for non-experts. Additional information, video tutorials, source code, and literature references are available at: *www.cellclassifier.org*

2. LICENSE

The software and all the materials are available at the *www.cellclassifier.org* website and are copyright protected.

Copyright (©) 2019 Peter Horvath. All rights reserved.
Advanced Cell Classifier (ACC) is licensed under the:
GNU General Public License version 3

ACC is a free software: you can redistribute it and/or modify it under the terms of the GNU General Public License as published by the Free Software Foundation, either version 3 of the License, or any later versions (at your option).

This program is distributed in the hope that it will be useful, but WITHOUT ANY WARRANTY; without even the implied warranty of MERCHANTABILITY or FITNESS FOR A PARTICULAR PURPOSE. See the GNU General Public License for more details.

2.1 THIRD-PARTY TOOLS

We included in our code the following third-party tools:

1) “dredviz”, https://research.cs.aalto.fi/pml/software/dredviz/. License: LGPL.

2) “track.pro”, by John C. Crocker. License: Freeware.

3) “Mulan”, http://mulan.sourceforge.net/. License: GNU General Public License.

4) “Weka”, https://www.cs.waikato.ac.nz/ml/weka/ License: GNU General Public License.

3. SYSTEM REQUIREMENTS

ACC is written in MATLAB (The MathWorks, Inc., Natick, MA, USA). The standalone version requires MATLAB Runtime. The source code requires the full software environment and additional toolboxes: Deep Learning Toolbox, Image Processing Toolbox, Statistics and Machine Learning Toolbox, and Bioinformatics Toolbox. The software is designed for MATLAB R2018b or newer versions. ACC works under Windows, Linux and OS X operating systems.

4. INSTALLATION

Starting from a compiled version of ACC:

1. Download the ACC compiled version for your operating system (*e.g*. Windows, Linux, and OS X environments) from: *www.cellclassifier.org*
2. Follow the instructions of the "How to install the Matlab runtime and run ACC" manual at: *http://www.cellclassifier.org/about-acc/*
3. Enjoy!

Starting from the source files of ACC:

1. Download the ACC source files from: *www.cellclassifier.org*
2. Extract the files from "ACC_v#.zip" to a folder without any white space in its path.
3. Open MATLAB.
4. Set MATLAB's path to the ACC folder containing the *startup.m* file.
5. Type "startup" in the MATLAB Command Window.

Please, read carefully the ACC's Documentation Manual to understand what type of files ACC requires as input, and what the main features of ACC are. Note: this manual focuses on the *Regression Plane* only, which is a module of ACC available at ACC version 3 (ACC v3.0) or higher.

5. GETTING STARTED WITH THE REGRESSION PLANE

As a start, follow the steps described in **"Sect. 4. Installation"** to install ACC to your computer and proceed as follows:

1. Download the sample dataset (named *Test-ProjectFolder01.zip*) available at the website: *www.cellclassifier.org*.
2. Extract the files into a local folder of your computer.
3. Open the ACC main window and click on: "File" -> "New project" to load the dataset (for "Plate type" select "*24 well (6x4)*").

Upon clicking the “OK” button, the "Image selector" window appears, and the main window of ACC changes as follows:

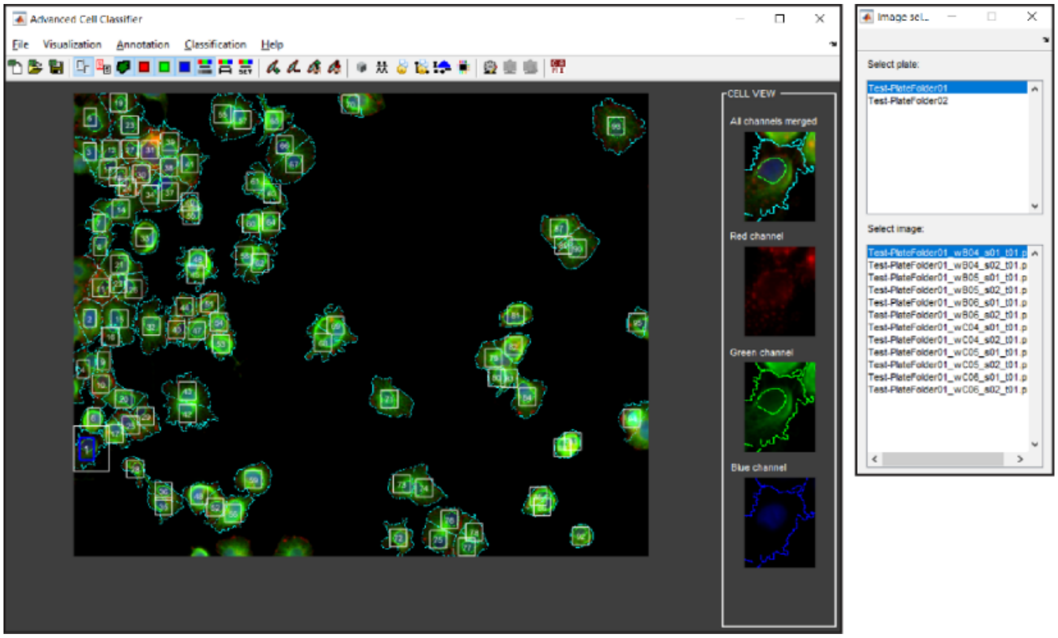

Fig. 1: Main GUI of ACC (when a dataset is loaded).

When a dataset is loaded, ACC is ready for cellular analysis. To analyse cells in the classical way, that is by categorizing the cells into separate phenotypic classes, follow these steps:

1. Create classes of interest.
2. Annotate cells to show different phenotypes of interest.
3. Train a classifier so that it can automatically classify non-annotated cells.

A more detailed description of the different classification options can be found in "*Video tutorial 3: Annotation possibilities*" at: *http://www.cellclassifier.org/about-acc/*

With the *Regression Plane* module, the user has now the opportunity to define "regression classes" for classifying the cells in a continuous way (*i.e.* without the need to define an infinite number of sub-classes for the intermediate stages).

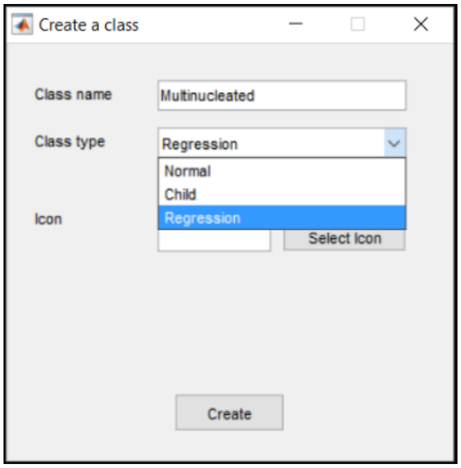

Fig. 2: GUI for creating a regression class.

The *Regression Plane* automatically opens by clicking on a cell and then on the icon of the regression class. The selected cell may then be placed by the user in a specific *(x, y)* position on the *Regression Plane*. Finally, once a regressor is trained, all cells of an image (or more images) will be automatically associated to an *(x, y)* position of the plane. Please note that in this documentation "classifier" refers to the model trained to classify cells onto different discrete phenotypic classes (including the regression ones), while "regressor" refers to the model trained to predict an *(x, y)* position on the *Regression Plane*.

A typical sample workflow:

1. Start ACC by typing "startup" in the MATLAB Command Window.
2. Open a dataset with "File" -> "New project".
3. Create a regression class with "Annotation" -> "Create a new class".

4. Browse the images and annotate several cells for the defined regression class by selecting a cell and then locating it in an *(x, y)* position of the *Regression Plane*.
5. Train a regressor from the *Regression Plane* GUI with the "Train regressor" button.
6. Check the training quality from the *Regression Plane* GUI with the "Predict images" button.
7. Classify the cells of an entire plate with "Predict plates" and see the automatically generated output files.

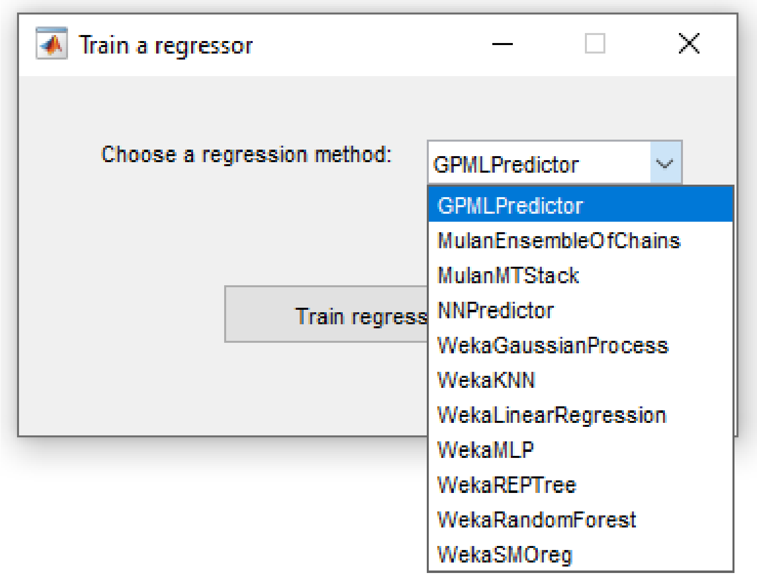

Fig. 3: List of available regressors.

6. REGRESSION PLANE GRAPHICAL USER INTERFACE (GUI)

Once a regression class is defined, the *Regression Plane* GUI opens.

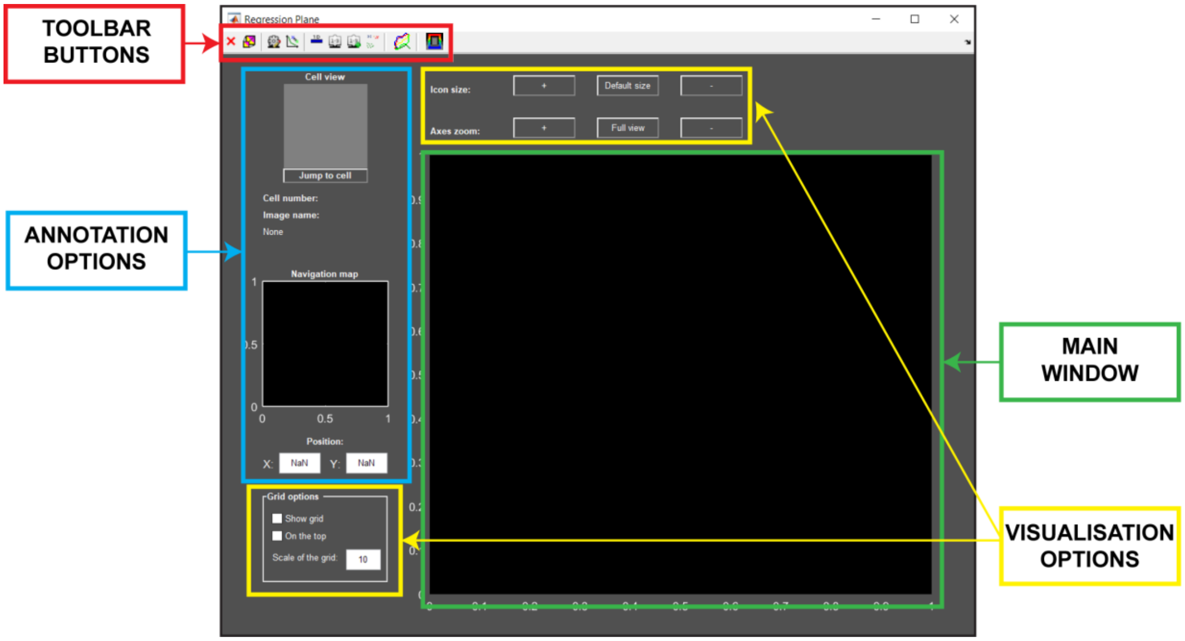

Fig. 4: *Regression Plane* GUI.

6.1 MAIN WINDOW

The *Regression Plane* is like a 2D blackboard with axis labels ranging from *0* to *1*. Each annotated cell will be displayed directly on the *Regression Plane*. Click the left-button of the mouse to place a cell anywhere on the plane. Select a cell on the *Plane* by clicking the left-button: a blue frame will appear around the selected cell. The selected cell can then be dragged and dropped anywhere on the Plane.

6.2 VISUALIZATION OPTIONS

We provided several buttons to adjust the magnification of the axis and the size of the cell icons displayed on the main window of the *Regression Plane*. The "Grid options" buttons simply allow the visualization of a grid on the main window to help the user better define the *(x, y)* position of the cells in the *Regression Plane*. The user can set the scale of the grid (*i.e*. distance between the parallel lines), and the position of the

grid with respect to the cells' icons. Finally, mouse scrolling results in zooming-in and -out in the *Regression Plane*.

6.3 ANNOTATION OPTIONS

To select a cell that is already located on the *Regression Plane* simply click on it. The selected cell will be displayed on the "Cell view" window. The ID of the cell and the name of the original image are shown under the "Cell view" window. The position of the selected cell on the *Regression Plane* is indicated by a red star in the "Navigation Map", while its coordinates appear in the "X-" and "Y-position" boxes, which are editable. To visualise the image containing the selected cell in ACC, just click on the “Jump to cell” button, placed under the “Cell view” window.

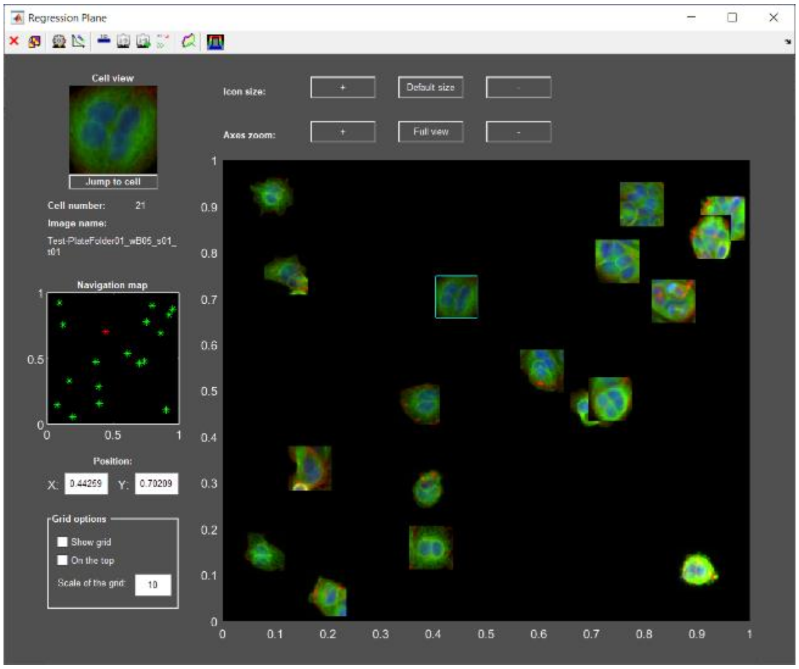

Fig. 5: *Regression Plane* with a selected cell.

To select more than one single cell and move the block of selected cells into a new position of the *Regression Plane*:

1) Press the ‘R’ key on the keyboard;
2) Move the mouse, the cursor will change into a white-cross;
3) Click on several points of the *Regression Plane* to define a polygon around the cells;
4) Double-click with the left-button of the mouse to close the polygon.

After that it will be possible to drag-and-drop the block of selected cells, to place them in a new position of the *Regression Plane*.

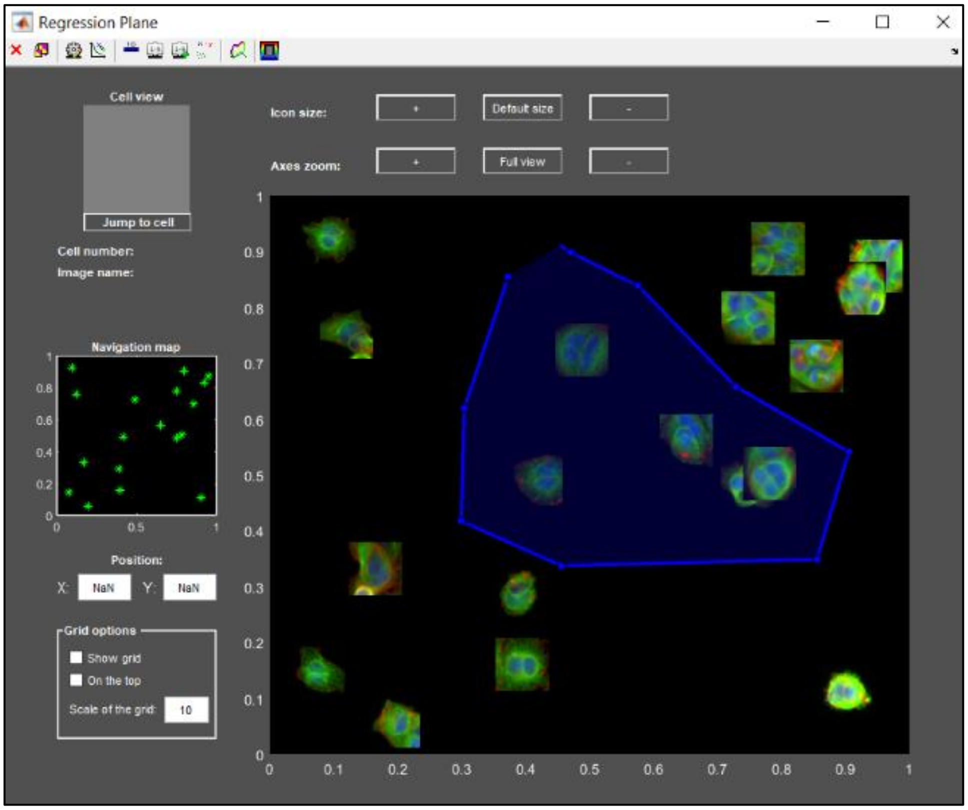

Fig. 6: *Regression Plane* with a block of selected cells.

6.4 TOOLBAR BUTTONS

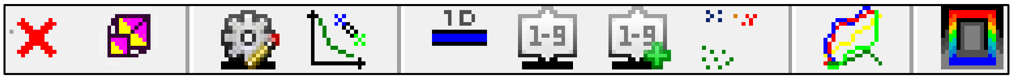

Fig. 7: Toolbar buttons of the *Regression Plane*.

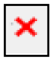

Delete button: To delete a cell from the *Regression Plane*.

Adapt cell size with zooming: To fix the ratio between the size of the icon of the annotated cells and the *Regression Plane* magnification. Practically, if this button is active the icons of the cells will enlarge by scrolling the mouse.

Train a regressor: To train a regressor using the annotated cells. For non-computer experts we suggest using the "GPML Predictor" algorithm that generally provides good results.

Measure performance: by clicking this button it is possible to check how the regressor performs on the training set. White vectors indicate the difference between the position defined by the user and predicted by the regressor. The predicted coordinates are calculated by 3-fold cross validation, and the results can also be exported to a csv file.

Constrain to 1D annotation: To force the annotation of the cells to a line of the *Regression Plane*.

Predict image(s): To predict all the cells of selected images.

Predict plates: To classify the cells of all the images of selected plates.

Meta-visualization of treatments: Heatmaps (*i.e.* well-based 2D distribution of cells in the 2D *Regression Plane*) visualization for each well of the plate obtained by using the Dredviz package (see: *http://research.cs.aalto.fi/pml/software/dredviz/*), typically corresponding to a specific cell treatment.

Trajectory Plot: To visualise lines connecting instances of the same cell. Time-point information are needed to run this functionality.

Color Frame: To visually analyse the values of a selected feature of cells displaced on the *Regression Plane*. The feature's value is represented with a border with colour ranging from blue to red, shown around the thumbnails of the cells.

7. ANNOTATION WITH THE REGRESSION PLANE

ACC offers three possibilities to annotate cells in the *Regression Plane*. First the user creates a regression class. Then, the user can annotate cells in the *Regression Plane* by:

- Manually defining the *(x, y)* position.
- Predicting the cells of one image and then moving the cells of interest to specific *(x, y)* positions, or simply double clicking on a cell to annotate it in the training set.
- Using the active regression module.

7.1 MANUAL ANNOTATION

To manually define the position of a cell on the *Regression Plane* the user simply left-clicks on a cell in the main window, and then left-clicks on the icon of a regression class. The *Regression Plane* of that specific class will appear. To locate the cell in a specific *(x, y)* position, click on the *Regression Plane*. To redefine the position of a cell, click on the cell (a blue border will appear around the selected cell) and write the coordinate values in the "X-" and "Y-position" editable boxes (below the "Navigator map", on the left side of the *Regression Plane* GUI), or simply drag and drop the cell in the desired position. The user can follow this strategy also to redefine the *(x, y)* position of cells already annotated in the *Regression Plane*. Note that the *Regression Plane* opens simply by clicking on the small "R" button in the bottom-right-corner of the class icon in the main GUI of ACC.

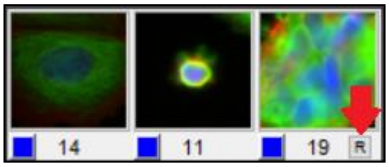

Fig. 8: Icon of a regression class.

To increase the precision of the manual annotation strategy, we implemented the *Color Frame* module. It allows to qualitative analyse the value of any selected feature of the cell, so to better understand which one can be the best position of that cell in the *Regression Plane*. In particular, by selecting a feature and enabling the *Color Frame*, all the cells will be visualised with a coloured border representing the feature value associated to that specific cells. The colour of the border will range from blue to red, and this helps the user to see the differences between cells. By clicking on the *Color Frame* toolbar icon, the *Color Frame* parameter selector window will appear. The user can then select a feature from the list and check the distribution of values of selected feature. The colour bar represents the colour tone that will be assigned to the border of the cells displaced on the *Regression Plane*. The user can limit the range of values of interest and choose between linear and logarithmic colour scale types.

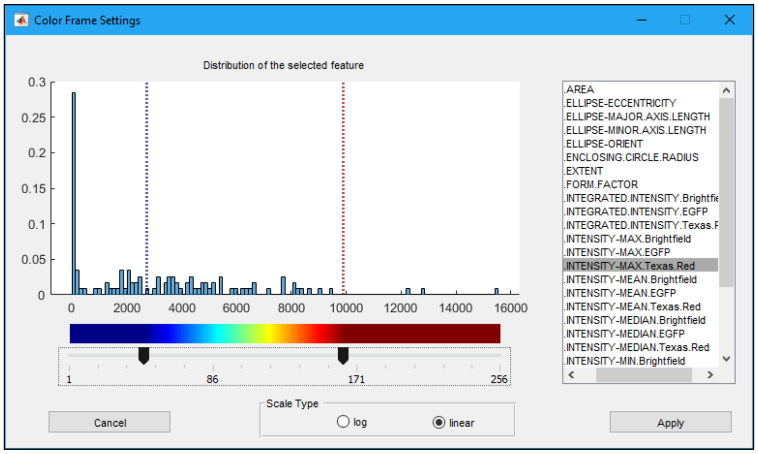

Fig. 9: *Color Frame* module: main window.

It is worth noting that the *Color Frame* module can be useful in the "Prediction" mode too. By enabling the *Color Frame* In the "Prediction" mode, the user will be able to qualitative analyse the distribution of value of the selected features. Note that if there are picks for which most of the cells will be represented in only a few bins of the histogram, these overpopulated bins will make invisible other bins with a small number of cells. In this case with a right-click on the histogram, the user can change the scale type of the y-axis to logarithmic and make the small bins visible.

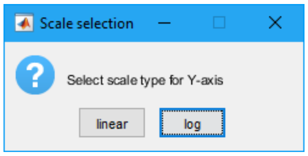

Wizard for selecting the scale for the Y-axis. To open it, right click on the histogram.

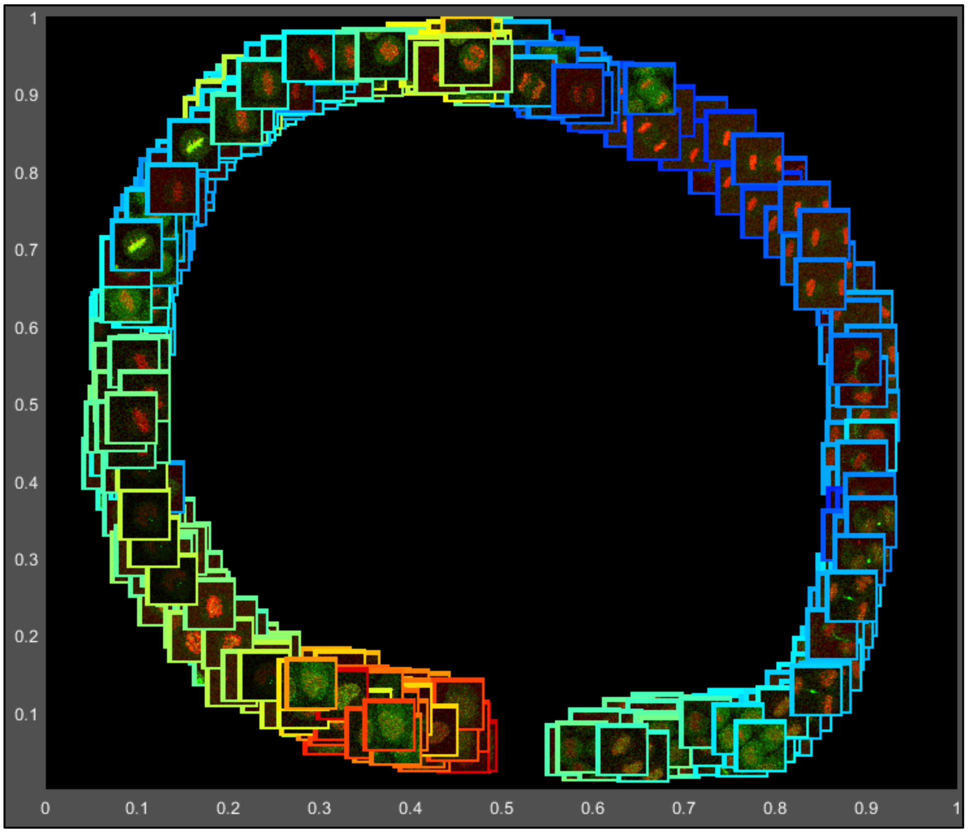

Fig. 10: *Color Frame* module: example of visualisation, feature “nuclear area”.

7.2 GUIDED ANNOTATION

The user can use the "Predict images" button to automatically annotate all the cells of selected images. An image selector will appear after pressing the "Predict Images" toolbar button in the *Regression Plane* GUI.

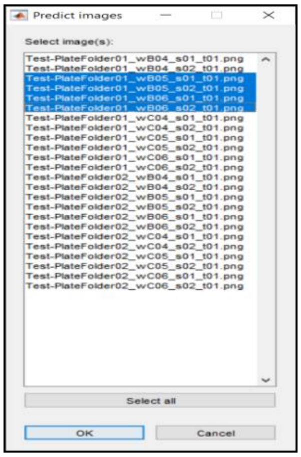

Fig. 11: Image selector of the Predict Image GUI.

Every cell in the selected images, classified into this regression class, will be located on the *Regression Plane*.

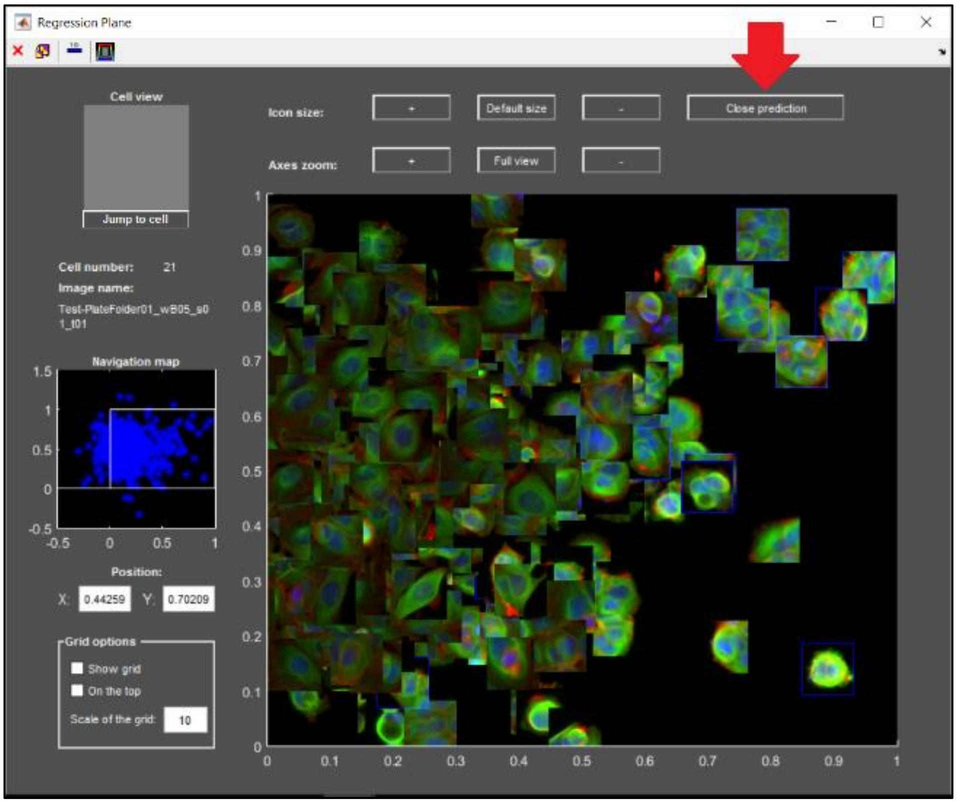

Fig. 12: Prediction plane with the "Close prediction" button.

To move any of these cells into the training dataset the user may:

1. Left-click on the cell and drag and drop it to the desired *(x, y)* position.
2. Alternatively, the user can simply double click on the cell (multiple selections are also available). This way the cell(s) will be considered as part of the training dataset.

Note that the annotated cells will appear with a blue frame.

7.3 ACTIVE REGRESSION

The general idea of "Active learning" algorithms is to help the user to improve the decision ability of the machine learning model by on-line selection of the minimal set of cells. In ACC, "Active learning" algorithms first automatically select cells that are difficult to classify, then ask the user to manually annotate them. This process results in a better boundary definition between the classes. We extended the idea of "Active learning" to "Active regression", forcing the computer to propose cells that are difficult to be automatically located in the *Regression Plane* or to decrease uncertainty as much as possible.

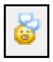

Active learning: To enable/disable active learning, to force the classifier to propose cells difficult to be classified.

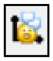

Active regression: To enable/disable active regression, to force the regressor to propose cells difficult to be automatically located in the *Regression Plane*.

The "Active regression" algorithms currently implemented in ACC are:

- Empty Regions 2D.
- Greatest Distance.
- Out of Bounds.
- Overall Uncertainty Sampling.
- Query by Committee.
- Uncertainty Based.

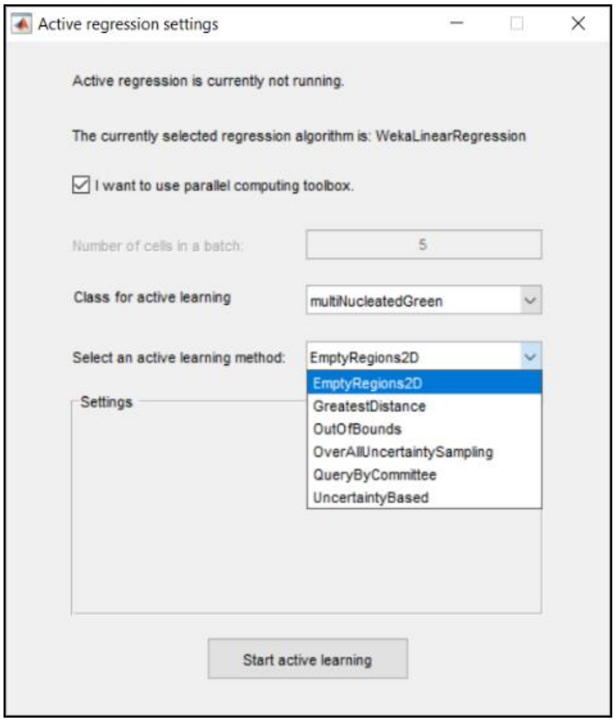

Fig. 13: Active regression settings GUI.

For non-computer experts we suggest using the "Empty Regions 2D" algorithm, which does not require settings and generally provides good results.

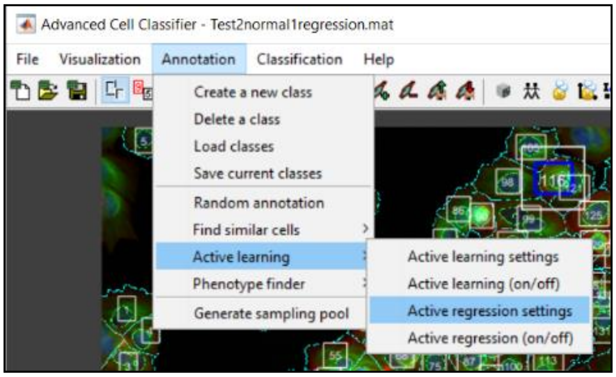

Fig. 14: Path for changing the active regression settings.

It is worth noting that after pressing the "Active regression" button the computer will require the generation of a sampling pool to speed up the computation. The "Generate sampling pool" function reduces the dataset of cells to be considered for analyses. When the "Generate sampling pool" button is clicked, a window appears asking the user for the "desired sampling ratio", that is a number *x*, where $0 < x \leq 1$ represents the

percentage of the original dataset to be kept for further consideration. Accordingly, if "desired sampling ratio" is set to *1* the entire original dataset is used.

8. OUTPUT OF THE REGRESSION PLANE

The main aim of the *Regression Plane* is to classify cells in a continuous manner.

8.1 CELL-BY-CELL PREDICTION

Each cell will be associated to an *(x, y)* position on the *Regression Plane*. To see the coordinates of each cell classified in a regression class, follow these steps:

1. Create at least one regression class.
2. Open the *Regression Plane*.
3. Train the regressor on the main GUI of the *Regression Plane*.
4. From the main GUI of ACC, select one image of interest, and press the "Predict current image" button.
5. Enable the "Cell classes" visualization.
6. Click on a cell belonging to the regression class.

A red box will appear over the selected cells, showing the coordinates of that specific cell in the *Regression Plane*.

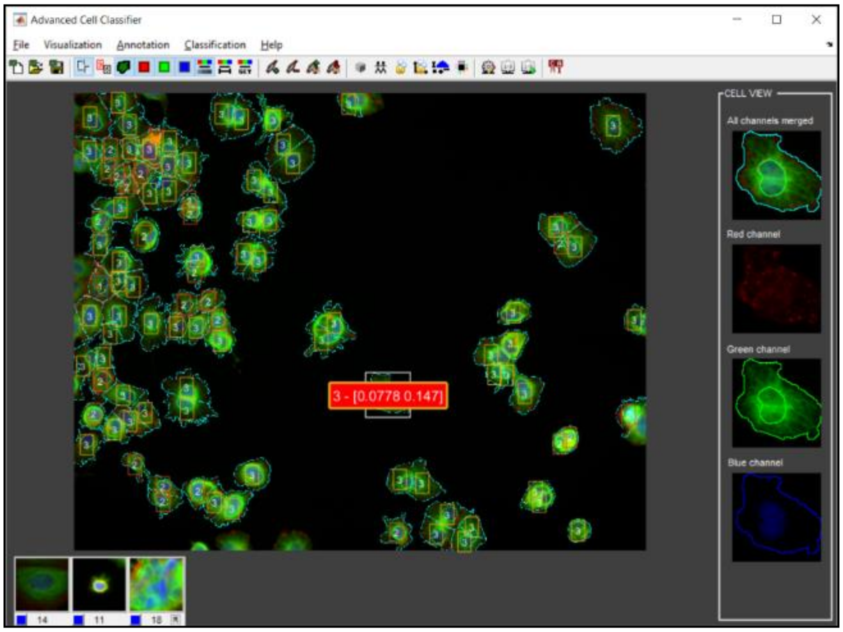

Fig. 15: Cell-by-cell prediction.

If the "Measure performance" button is active, white vectors will show the difference between the position defined by the user and the position predicted by the regressor for all the cells of the training set.

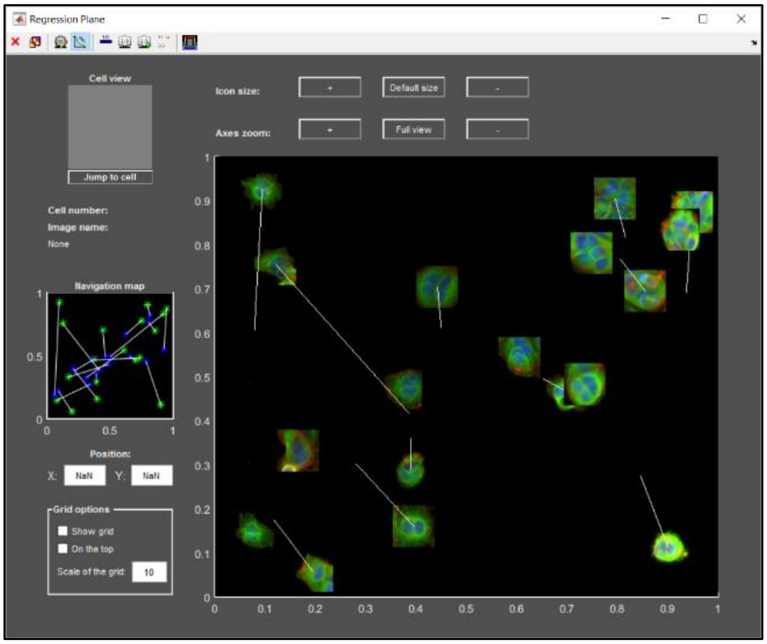

Fig. 16: Error vectors between predicted and defined position of the annotated cells.

8.2 PREDICT IMAGES

Once several cells have been annotated in the *Regression Plane*, it is possible to use the "Predict images" toolbar button of the *Regression Plane* GUI to predict the *(x, y)* position of all the cells (of the considered regression class) belonging to selected images. The cells will be automatically located in the *Regression Plane*.

8.3 PREDICT PLATES

Press the "Predict plates" button to run a regression analysis of all the images (of one or more plates). A user-friendly GUI automatically opens by clicking on the "Predict plates" button.

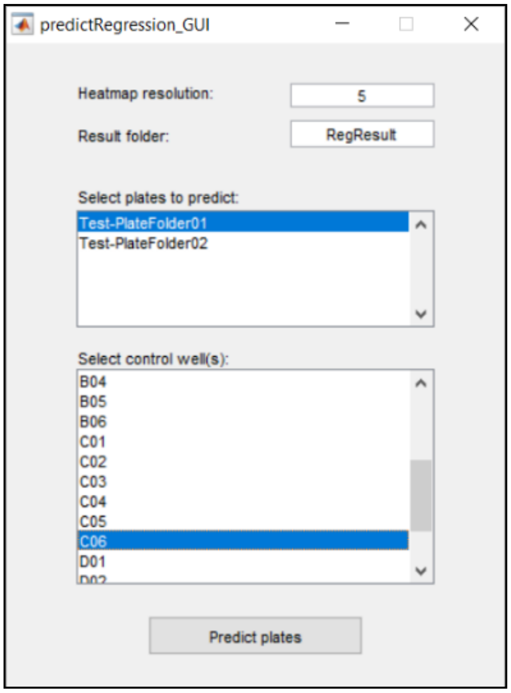

Fig. 17: GUI: predict plate settings.

The number of "pixels" used to represent the heatmap of the *Regression Plane*, obtained for each analysed well, can be defined with the "Heatmap resolution" setting. A high value (*e.g. 10*) generates detailed maps,

generally less useful to explore the principal components of the *Regression Plane*. We set *5* as default value. In general, the average number of cells in the considered image must be significantly higher than the resolution value set.

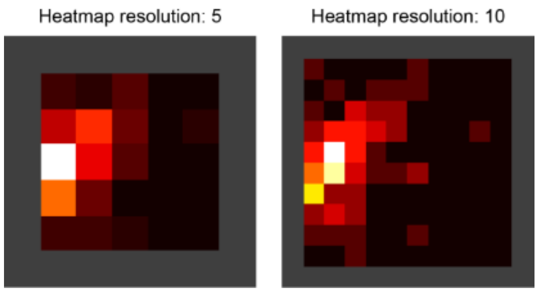

Fig. 18: Heatmap of a single well (shown at different resolutions).

The output files will automatically be saved in a folder named "RegResult" ("RegResult" is the default name, the user can change it) in the path of the root-folder of the images analysed. Once computation finishes, a pop-up message reports the full path of the output folder. It is possible to select the plates to be analysed and the wells to be consider as control for the analysis from an intuitive plate and well selector.

Once the parameters are set, a pop-up message will ask for a *.csv* file containing a description of the treatment (typically, the drug name) of each well. The *.csv* file is composed of two columns and one row for each well of the plate that has been imaged. Each row is composed of two items separated by a comma. The first line of the file simply reports the column identifiers: "WellID" and "TreatmentName". Then, each row reports the ID of the well and the name of the treatment.

```
WellID,         TreatmentName
B04,            Treatment-01
B05,            Treatment-02
B06,            Treatment-03
C04,            Treatment-04
C05,            Treatment-05
C06,            Control
```

In case the plate layout is not available, simply select the "plate layout not available" option in the pop-up message appearing. The computer will then generate a default plate layout.

Whilst running a full-plate prediction, *Regression Plane* offers various unsupervised methods to help you understand the results. There are 4 methods available for this currently:

- Principal Component Analysis (PCA)

- Neighbourhood Retrieval Visualizer (NeRV)
- Stochastic Neighbour Embedding (t-SNE)
- Hierarchical clustering of the treatments (hierarchical clustering)

When you are asked, you may specify which of these methods you wish to run for your data (including all or none of these).

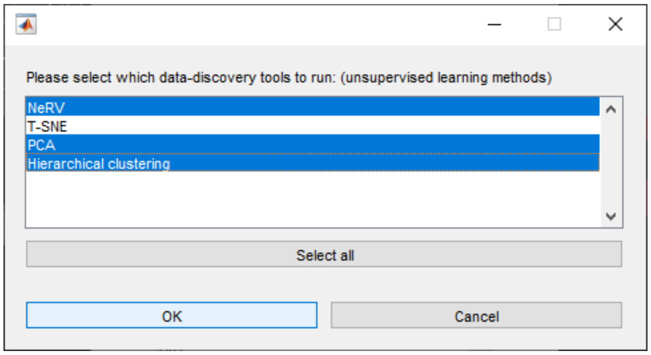

Fig. 19: Unsupervised learning methods for data discovery.

Additionally, these methods require an icon that represents the well, which may be the discrete *Heatmap* or the smoother *Kernel Density Estimation*.

Finally, the files automatically saved to the "RegResult" folder are:

- *[PlateName]_regressionReport_[classID].pdf*
- *[PlateName]_singleCellRegressionPositions.csv*
- *PlotOfPlots_[PCA/NeRV/T-SNE].pdf*
- *treatmentComparison_Distances.pdf*

DESCRIPTION OF THE "*[PlateName]_regressionReport_[classID].pdf*" FILE

The file *[PlateName]_regressionReport_[classID].pdf* (where *[PlateName]* is the name of the plate analysed, and *[classID]* is the progressive ID number of the considered class, *e.g.* Test-*PlateFolder01_regressionReport_class03.pdf*) contains the following, in a plate-based format (*e.g*. multi-well plate with 24 wells, disposed in 4 rows, 6 columns):

- Heatmaps of the single wells.
- Kernel density estimations of the single wells.

- Shift vectors of the population centum, showing the displacement between the maximum value in the control well (in this case *C06*) and the maximum value in the other heatmaps.
- Difference between the heatmap considered as control, and the other heatmaps, with blue areas representing regions where the current heatmap has lower values than the control one, and red areas where the current heatmap has higher values.
- Number of cells classified to the given regression plane.

Please note that if more wells are defined as a control in the "Predict plates" settings, the "shift vectors of the population centum" and the "difference between the control heatmap and the other heatmaps" will be computed with an average heatmap obtained by averaging the heatmaps of the wells considered as control.

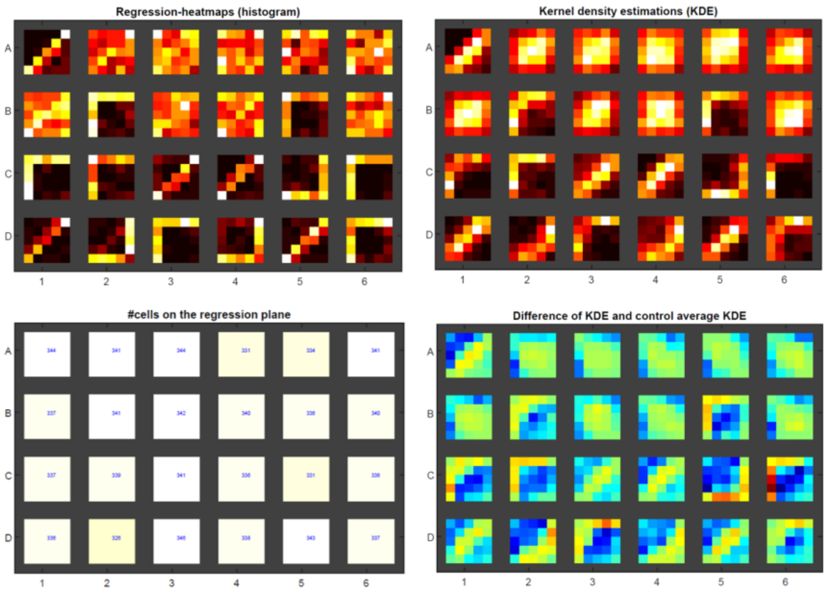

Fig. 20: Regression report.

DESCRIPTION OF THE "*[PlateName]_singleCellRegressionPositions.csv*" FILE

It is a comma separated file containing several parameters for each cells of the plate, considered in the regression class analysed. It contains the following:

- Plate name.
- Row of the considered well.

- Column of the considered well.
- Image name.
- Object number (*i.e.* ID of the cell in the analysed image).
- x-coordinate position of the cell in the analysed image.
- y-coordinate position of the cell in the analysed image.
- x-coordinate position of the cell in the *Regression Plane*.
- y-coordinate position of the cell in the *Regression Plane*.

Each row of the *[PlateName]_singleCellRegressionPositions.csv* file corresponds to a cell belonging to the analysed regression class:

```
PlateName,   Row, Col, ImageName, ImageNumber, ObjectNumber, xPos,  yPos,  regPosX, regPosY
TestPlate01, B,   4,   wB04_s01,  1,           4,            30.6,  785.4, 0.535,   0.534
TestPlate01, B,   4,   wB04_s01,  1,           6,            37.4,  496.9, 0.148,   0.127
TestPlate01, B,   4,   wB04_s01,  1,           30,           106.1, 255.3, 0.922,   0.534
TestPlate01, B,   4,   wB04_s01,  1,           37,           128.1, 733.3, 0.112,   0.510
...
TestPlate01, B,   5,   wB05_s02,  2,           1,            18.3,  538.1, 0.324,   0.498
...
```

DESCRIPTION OF THE "*PlotOfPlots_[PCA/NeRV/T-SNE].pdf*" FILES

*PlotOfPlots.pdf*s offer several visualization methods to help users discover treatment similarities. These methods compare your wells and treatments to each other according to the characteristics of the regression plane. In principle, they automatically highlight differences and similarities in your treatments by visualizing them on a 2D plane. On this plane the wells placed close are similar to whilst those more distant are different from each other. If replicates are present in the plate layout, they are highlighted in the *PlotOfPlots* with the same colour.

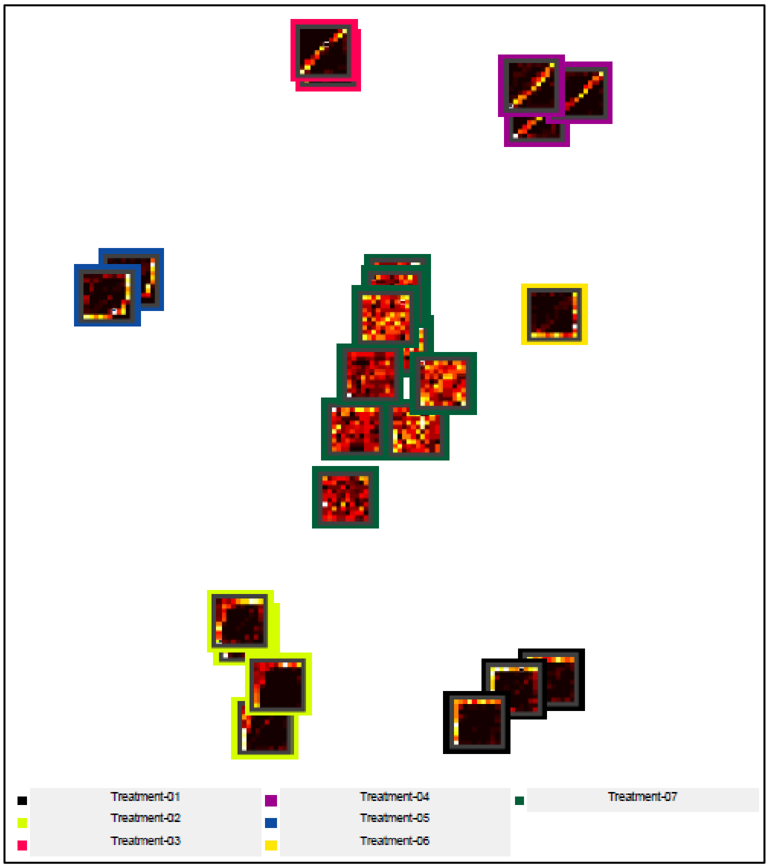

Fig. 21: Plot of plots: heatmaps.

DESCRIPTION OF THE "*treatmentComparison_Distances.pdf*" FILE

Besides the visualization in the *PlotOfPlots*, a hierarchical clustering of the treatments is also performed. A clustergram showing hierarchical connections between the different treatments is reported in the *treatmentComparison_Distances.pdf* file. This provides an alternative way to compare different treatments. The dendrogram on the left represents the connections between different treatments; similar treatments are connected with shorter lines. The metric used to create the clustergram shown in this file is the "Distance" in the *Regression Plane* of the different heatmaps. "Distance" refers to the Kullback-Leibler Divergence of the kernel density estimations of the treatments considered, which measures how similar the two population is.

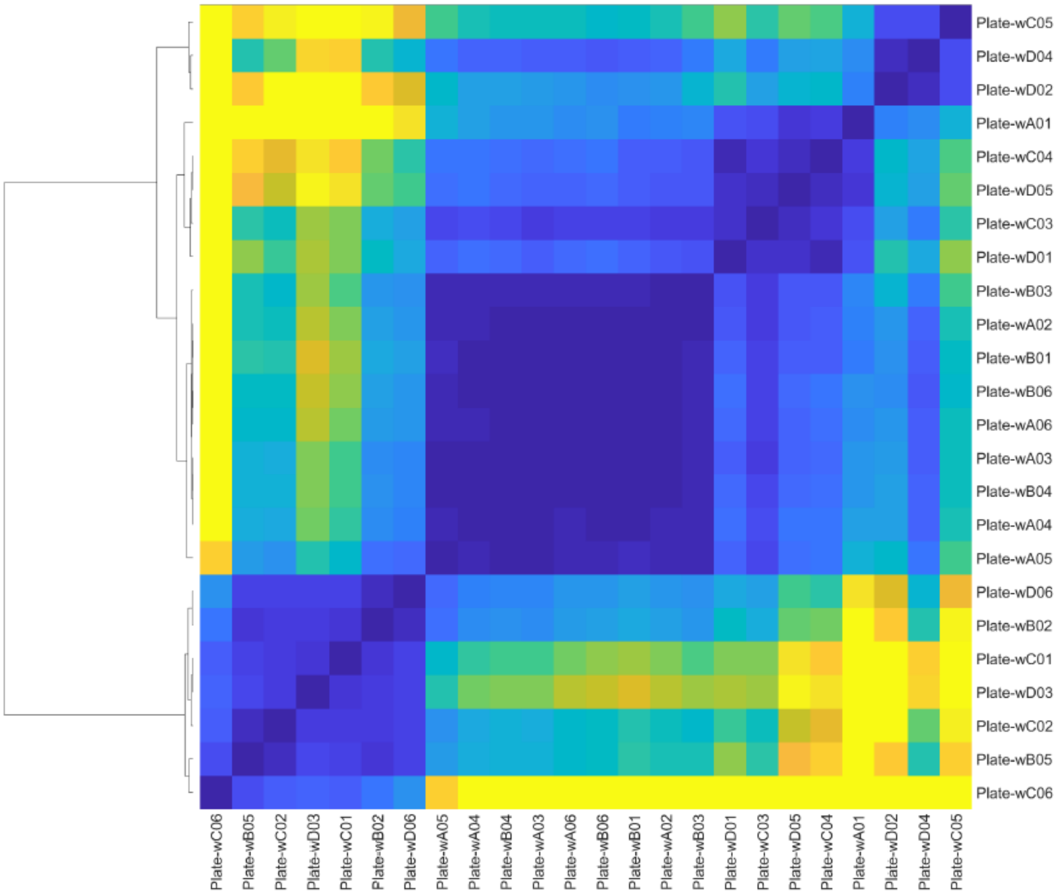

Fig. 22: Distance-based clustergram.

8.4 WELL-BASED ANALYSIS OF THE TREATMENTS

The *Regression Plane* can also be used to visualize the position of the heatmaps of the different wells, corresponding to different treatments. After clicking on the "Meta-visualization" button, the user has to select the output folder containing the regression analysis to be visualized. The output folders containing the regression analysis are automatically saved inside the "RegResult" folder, using the following nomenclature:

yyyymmddhhmm_[PlateName]

y stands for year, *m* for month, *d* for day, *h* for hour, *m* for minute and *[PlateName]* for the name of the analysed plate, for instance:

201705201837_Test-PlateFolder01

Please note that the output folder containing the regression analysis always contains a folder named "*tmp*" with the files required for the visualization in the *Regression Plane* of the heatmaps.

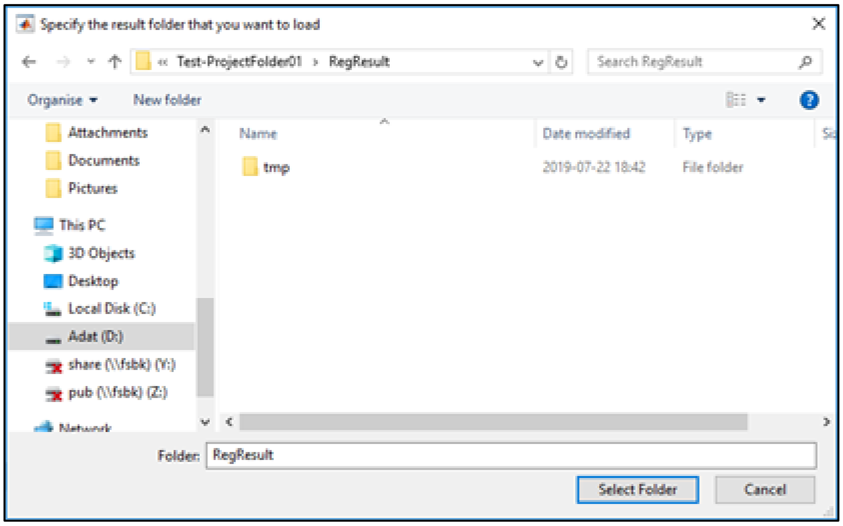

Fig. 23: Output folder containing the "temp" folder.

Once an output folder containing the regression analysis files is selected, the heatmaps, corresponding to the different wells of the analysed plate, will be visualized on the *Regression Plane* to help quantitative comparisons of the different treatments. The name of the corresponding well will automatically be visualized on the left side of the GUI by clicking on a heatmap.

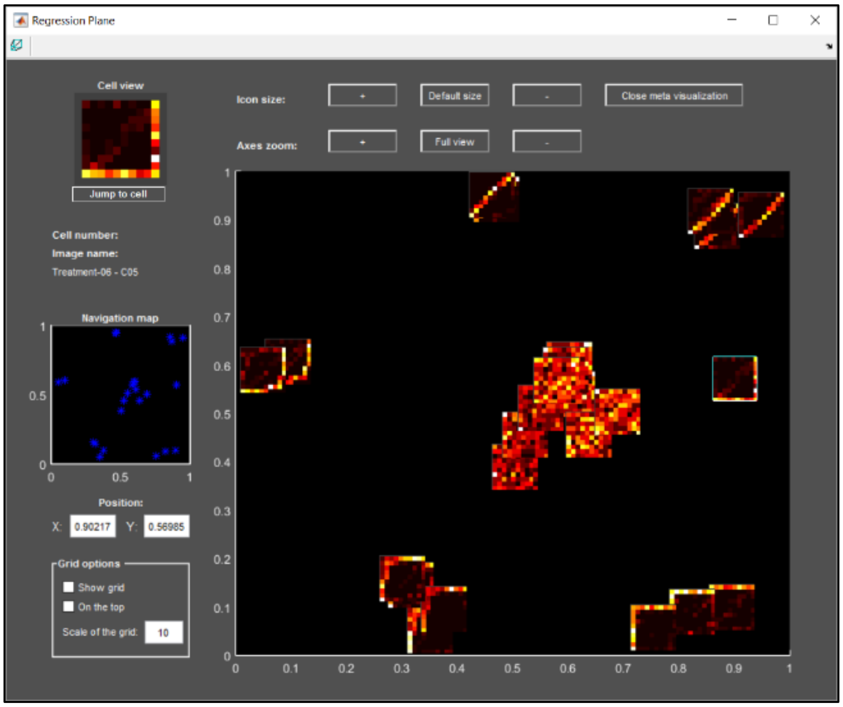

Fig. 24: Meta-visualization of the heatmaps.

8.5 TRAJECTORY PLOT

Trajectory Plot is a multifunctional visualization tool that allows a deeper understanding of processes represented in the *Regression Plane*. Practically speaking, the *Trajectory Plot* uses time-point information to create trajectories (*i.e*. lines) between instances of the same cell displaced in the *Regression Plane*. It draws a line between the subsequent instances keeping their coordinates on the *Regression Plane*.

Trajectory Plot requires time-point information and provides several options to:

(*a*) filter the data,

(*b*) create groups of cells with similar trajectories,

(*c*) visualise the different positions in the *Regression Plane* of the instances of the single cells,

(*d*) compare groups of trajectories coming from cells with similar characteristics.

In the next sections, all main features of the *Trajectory Plot* GUI will be described in detail.

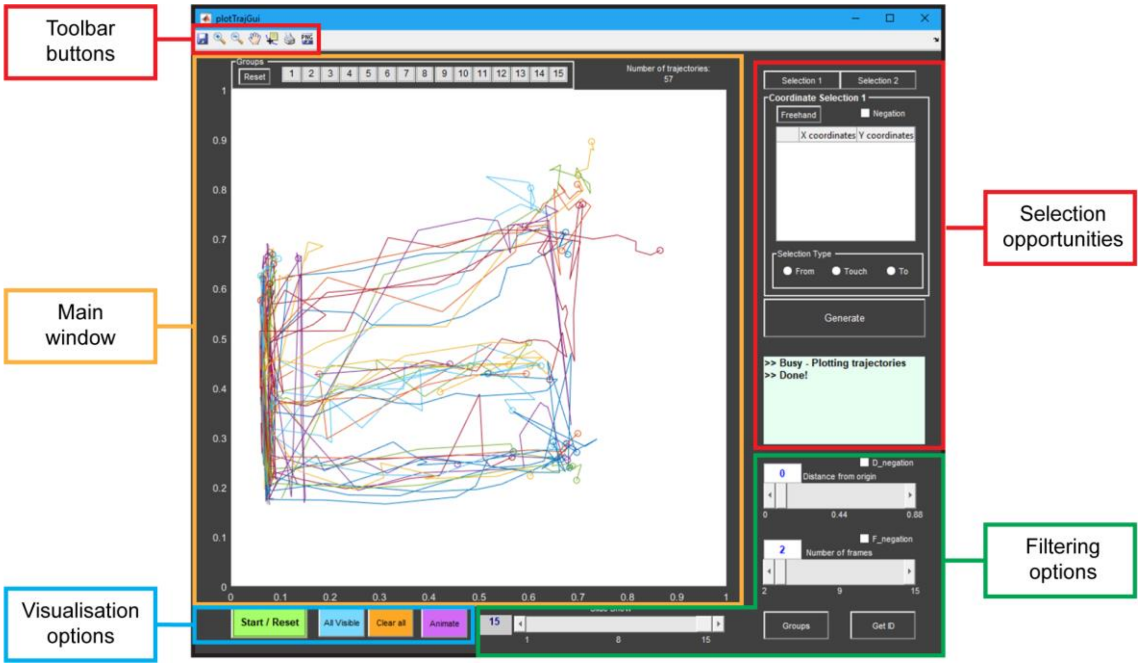

Fig. 25: *Trajectory Plot* GUI with main sections.

SECTION: TOOLBAR BUTTONS

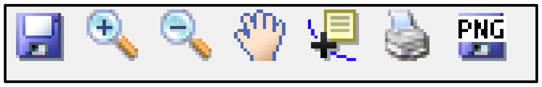

Fig. 26: *Trajectory Plot* toolbar buttons.

What follows is a brief description of the different toolbar buttons available on the *Trajectory Plot*:

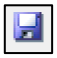

Save button, to save the current figure shown on the main window.

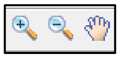

Zoom-in, zoom-out, and pan tool, to zoom and drag the object on the main window.

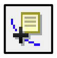

Data tip: to show the coordinates on the *Regression Plane* of the selected point.

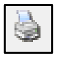

Print, to print the current figure shown on the main window

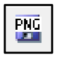

Export, to save a printscreen image of the axes.

SECTION: MAIN WINDOW

The *Trajectory Plot* main window offers an alternative representation of the *Regression Plane* main window. Instead of icons of the cells, the *Trajectory Plot* Main Window shows the trajectories (*i.e.* lines) connecting the different instances of the same cell, with a dot representing the coordinates where the cell icon is shown in the *Regression Plane*. The axes' labels range from 0 to 1, like in the *Regression Plane*.
Click on a trajectory to select it: the line will turn to a blue colour and a *Line Property* window automatically opens to allow changing the visual appearance of the trajectory. With the buttons described in the next sections, it is also possible to create groups of trajectories of similar cells, to have a better understanding of the biological process.

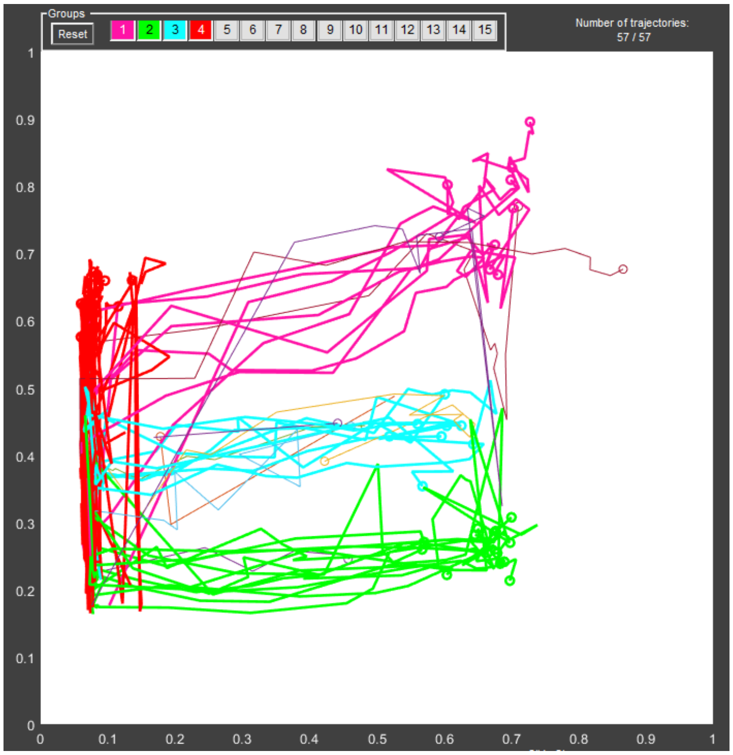

Fig. 27: *Trajectory Plot* GUI, main window with groups of trajectories.

Note that the starting point of each trajectory is represented by a small circle of the same colour:

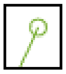

End point of a trajectory

The “Trajectory group buttons” and the display reporting the number of trajectories currently visualised can be found at the top of the Main Window of the *Trajectory Plot* GUI.

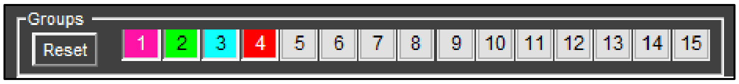

“Trajectory groups”: The user has the possibility to group trajectories in “groups”. Once a group is created, a button in this section will be associated to that group and will enable/disable the visualisation of the trajectories of that group.

SECTION: VISUALISATION OPTIONS

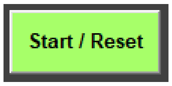

“Start/Reset” button: to visualise the trajectories, just click on this button once. If you click on it again, it will clear the Main Window by setting all the options and values to default.

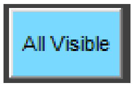

“All Visible” button: to render all the trajectories on the Main Window, resetting the filters.

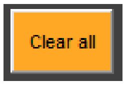

“Clear All” button: to hide all trajectories currently visualised on the Main Window.

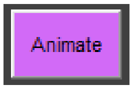

“Animate” button: to create and render a movie of the visible trajectories.

SECTION: FILTERING OPTIONS

The *Trajectory Plot* GUI provides several filter options to visualise/select trajectories based on their attributes.

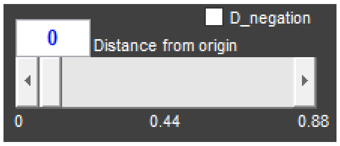

"Distance Slider": Hides the trajectories which have a distance between their start and end points that is lower than the "selected value" on the slider, used as threshold. The "threshold" value can also be directly written on the text window. Note that the "threshold" value must be between the min and max values shown under the slider. "D_negation": the distance negation flag makes the Distance Slider working in the inverse modality.

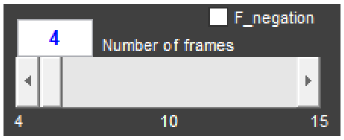

"Frame Slider": Hides trajectories with less time-points then the "selected value" on the slider. The "threshold" value can also be directly written on the text window. Note that the "threshold" value must be between the min and max values shown under the slider. F_negation: the frame negation flag makes the Frame Slider working in inverse modality.

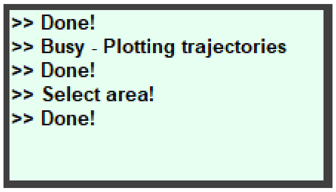

"Status Window": shows the status of the processor after the request of an action.

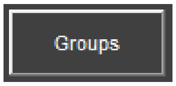

"Groups" button: to create a new group of trajectories or attach all the selected trajectories to an existing group.

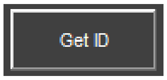

"Get ID" button: it allows creating a list of properties of the visible trajectories. The list of properties contains the "Trajectory ID", the original "Image name", where the cell comes from, and the "Cell ID" that is the original ID of the cell on the *Regression Plane*.

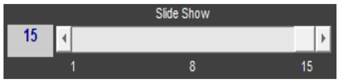

"Time Lapse Slider": Hides the trajectories which have at least one cell instance on a time-point higher than the "selected value" on the slider, used as threshold. The "threshold" value can also be directly written on the text window. Note that the "threshold" value must be between the min and max values shown under the slider.

SECTION: SELECTION OPPORTUNITIES

In this section we describe the opportunities provided by the *Trajectory Plot* to select trajectories based on their coordinates.

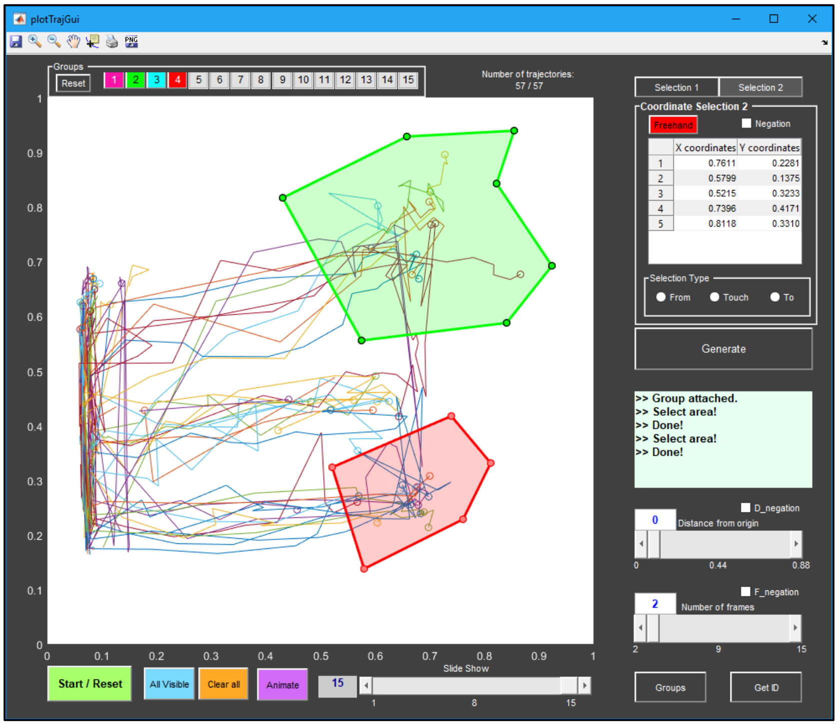

Fig. 28: *Trajectory Plot* GUI with trajectories manually selected.

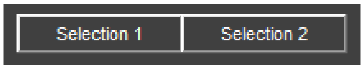

"Selection tab" buttons: to switch between two different selection areas. Note that both options can be used together at the same time.

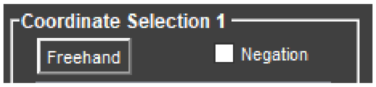

“Freehand selection”: it allows the selection of trajectories on the *Trajectory Plot*; select an area by defining the corners of a polygon. To close the polygon, just double click. “Negation”: it means an inverse selection of “Freehand selection”.

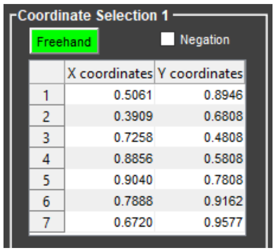

“Coordinates selection”: shows the corner points’ coordinates of the polygon used to define the “Selection 1”/”Selection 2” area.

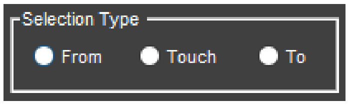

“Selection Type” buttons: They allow the filtering of trajectories based on one of the options described here: (a) “From”, selects all the trajectories with a starting point inside the selected area; (b) “Touch”, selects all the trajectories that have at least one cell instance inside the selected are; (c) “To” selects all the trajectories with an end point inside the selected area.

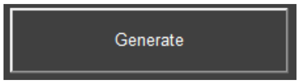

“Generate” button: to apply a selected filtering method on the trajectories with the rule previously set (*i.e.* “Selection Type” From/Touch/To).

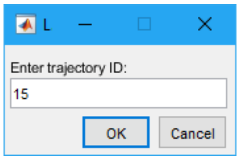

”Look-up” function: “f” key opens an input window to select a trajectory by its ID number, and opens its Line Property window.

SECTION: SLIDER SELECTOR

When right-clicking on the name of the sliders, the *Slider Selection* window opens. It gives the opportunity to calculate and set new values to the slider, so to filter the trajectories by different properties. There are a list-box of selectable properties which can be changed with radio buttons at the top “*Trajectory level”* and *“Cell level”*, a pop-up menu to select calculation *“Method”*, and two text boxes for setting *“Coordinate”* inputs.

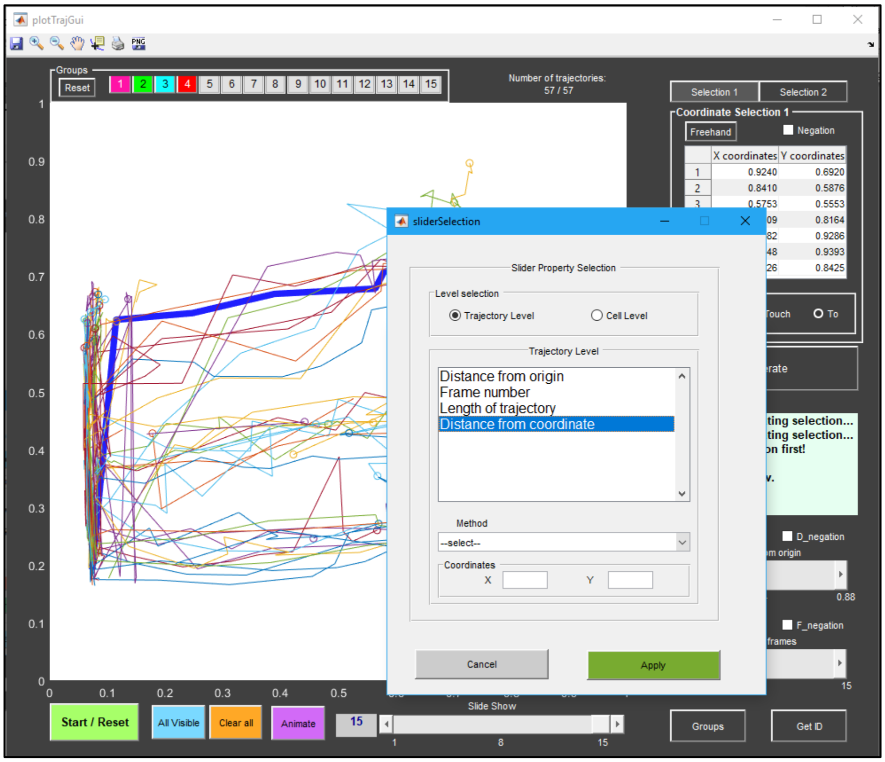

Fig. 29: *Trajectory Plot* module: main window of the Slider Selector.

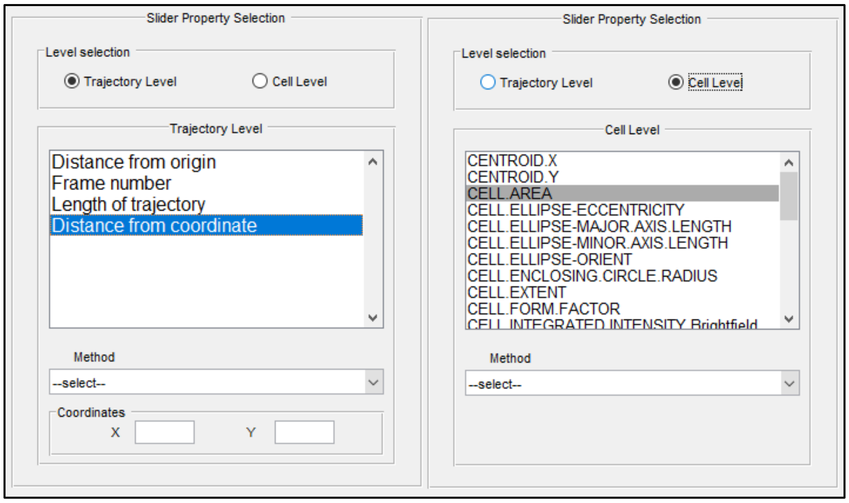

Fig. 30: *Trajectory Plot* module: Slider Selector Property list. On the "Trajectory Level" list-box (reported on the left), the user can select the properties of trajectories; on the "Cell Level" list-box (reported on the right) the user can select any "single-cell" feature then used to calculate the new values of the slider.

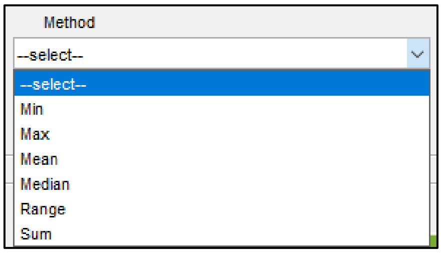

Fig. 31: *Trajectory Plot* module: list of possible measurements.

The Trajectory Level option calculates the value of the selected properties for each trajectory. Cell Level option calculates the new slider value by the given method on the selected feature of cells. For example, by selecting the "Area" feature and the "MAX" method, the calculation will compare the area values of a cell in each time-point of a trajectory and then will returns the maximum value. Clicking on the "Apply" button the new set of values will be used in the selected slider and rename the slider to the selected trajectory property or cell feature, then it is ready to filter by the new values.

SECTION: LINE PROPERITIES

When clicking on a trajectory, the *Line Properties* window opens. It contains information about the trajectory and the connected cell instances, giving an opportunity to: (*a*) modify the appearance of the trajectory, (*b*) jump back to the *Regression Plane*, (*c*) import thumbnail-images of the cell from the *Regression Plane*.

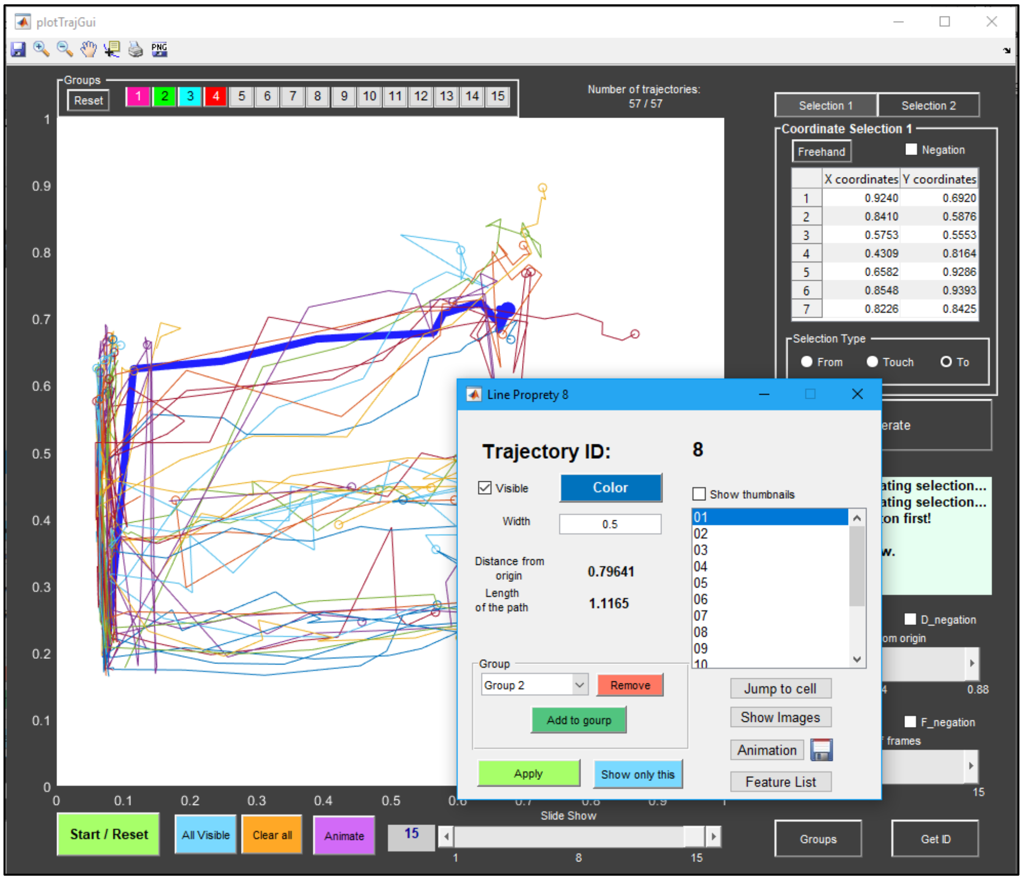

Fig. 32: *Trajectory Plot* GUI, Line Property window.

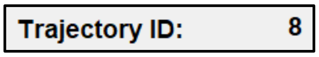

“Trajectory ID”: shows the ID of the selected trajectory in the *Trajectory Plot*. Note that this number is different from the Cell ID in the *Regression Plane*.

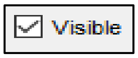

“Visibility” button: to set the visibility of the selected trajectory on/off.

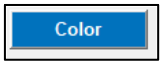

“Color” button: to change the colour of the selected trajectory.

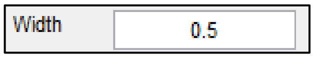

“Line width” box: to modify the actual value of the trajectory width.

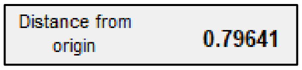

“Distance from origin” value: it reports the distance value between

the start and the end point of the trajectory.

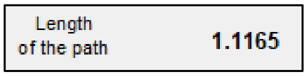

"Length of the path" value: it reports the length of the path, which is the sum of distances between the subsequent time-point instances of the cell.

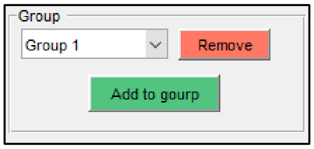

"Group" module: it allows to include/remove a trajectory from a group. The "Group pop-up list" shows the name of the groups currently available. The "Remove" button removes the selected trajectory from the groups. The "Add to group" button adds the selected trajectory to a specific group.

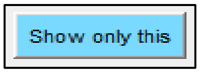

"Show only this" button: to hide all the other trajectories and show only the selected one.

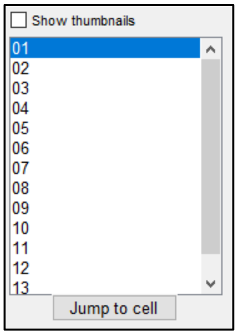

"Show Thumbnails": to import the thumbnail-images of the cell from the *Regression Plane* to the *Trajectory Plot*. "Thumbnail list": list of available thumbnails, ordered by time-point.

"Jump to cell" button: to jump back to the selected cell on the *Regression Plane*.

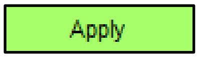

"Apply button": to set the changes and make them visible on the trajectory.

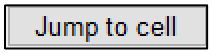

"Jump to cell": jumps back to ACC main window to this cell.

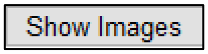

"Show Images": shows a new window containing all the representations (i.e. image) of the selected cell, extracted from the different frames.

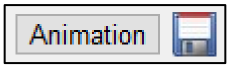

"Animation": creates a new window containing the animation of the "cell path" and the "cell images", simultaneously reporting for each point of the cell path the representation (i.e. image) of the selected cell. Use the "Save Icon" to save the animation (it will take a little bit of time).

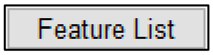

"Feature List": computes statistics and creates a table of selected features of the current cell. The table can be exported to a .CSV file.

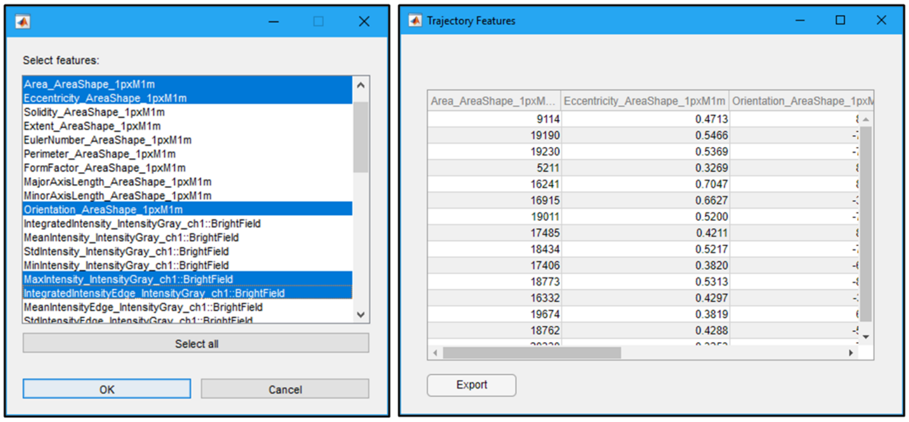

Fig. 33: *Trajectory Plot* module: feature list

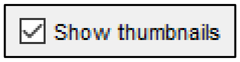

"Show thumbnails": places the images of the selected cell (i.e thumbnails) to the correct position in the 2D plane.

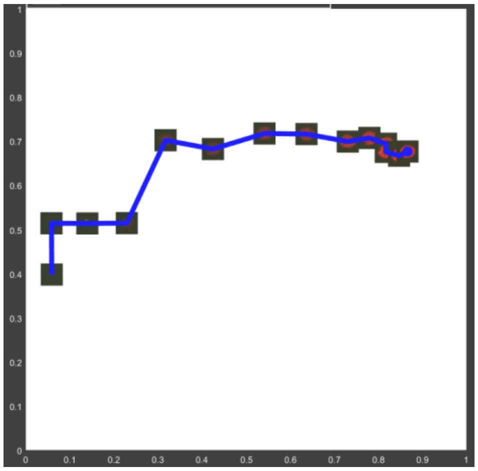

Fig. 34: Line with thumbnails of the cell

DESCRIPTION OF THE FILE CONTAINING THE TIME INFORMATION

Note that the *Trajectory Plot* requires time-point information to be able to create trajectories (i.e. lines) between the different instances of the same cell. There are two solutions to provide ACC with this information. First, in ACC there is a wizard to generate the *trajectory information* (i.e. time-point ".csv" file) based on the image nomenclature. Second, it is possible to use external resources to generate a tracking file, the format of which is described below.

What follows is a brief description of the different columns of the trajectory information file.

Column#1	*Column#2*	*Column#3*	*Column#4*	*Column#5*	*Column#6*
"Plate"	"ImageName"	"Frame"	"TrackID"	"tracking__center_x"	"tracking__center_y"
string	string	number≥0	number≥0	number≥0	number≥0

Fig. 35: *Trajectory Plot*, column overviews of the input ".csv" file

Column#1: "Plate" (string). Name of the folder containing the images of a plate.

Column#2: "ImageName" (string). Name of the image inside the "Plate" folder, containing the cell of interest.

Column#3: "Frame" (number≥0). Ordinal number indicating the discrete time point when the image containing the cell of interest was taken in the time-lapse sequence of images of the same field of view.

Column#4: "TrackID" (number≥0). Unique ID used to indicate a trajectory.

Column#5: "tracking__center_x" (number≥0). *x*-coordinate on the original "ImageName" image of the centre of the cell of interest (i.e. horizontal axis increasing from left to right).

Column#5: "tracking__center_y" (number≥0). *y*-coordinate on the original "ImageName" image of the centre of the cell of interest (i.e. vertical axis increasing from top to bottom).

Plate	ImageName	Frame	TrackID	tracking__center_x	tracking__center_y
Chromatin_Microtubules_P0013	Chromatin_Microtubules_P0013_T00031.jpg	31	120	1204	406
Chromatin_Microtubules_P0013	Chromatin_Microtubules_P0013_T00032.jpg	32	120	1200	404
Chromatin_Microtubules_P0013	Chromatin_Microtubules_P0013_T00033.jpg	33	120	1198	403
Chromatin_Microtubules_P0013	Chromatin_Microtubules_P0013_T00034.jpg	34	120	1196	403
Chromatin_Microtubules_P0013	Chromatin_Microtubules_P0013_T00035.jpg	35	120	1196	403
Chromatin_Microtubules_P0013	Chromatin_Microtubules_P0013_T00036.jpg	36	120	1196	402
Chromatin_Microtubules_P0013	Chromatin_Microtubules_P0013_T00037.jpg	37	120	1198	401
Chromatin_Microtubules_P0013	Chromatin_Microtubules_P0013_T00038.jpg	38	120	1199	400
Chromatin_Microtubules_P0013	Chromatin_Microtubules_P0013_T00039.jpg	39	120	1194	401
Chromatin_Microtubules_P0013	Chromatin_Microtubules_P0013_T00040.jpg	40	120	1195	402
Chromatin_Microtubules_P0013	Chromatin_Microtubules_P0013_T00041.jpg	41	120	1196	397
Chromatin_Microtubules_P0013	Chromatin_Microtubules_P0013_T00042.jpg	42	120	1187	394
Chromatin_Microtubules_P0013	Chromatin_Microtubules_P0013_T00043.jpg	43	120	1185	387
Chromatin_Microtubules_P0013	Chromatin_Microtubules_P0013_T00044.jpg	44	120	1187	384
Chromatin_Microtubules_P0013	Chromatin_Microtubules_P0013_T00045.jpg	45	120	1190	381
Chromatin_Microtubules_P0013	Chromatin_Microtubules_P0013_T00046.jpg	46	120	1192	377
Chromatin_Microtubules_P0013	Chromatin_Microtubules_P0013_T00047.jpg	47	120	1194	363
Chromatin_Microtubules_P0013	Chromatin_Microtubules_P0013_T00048.jpg	48	120	1195	364
Chromatin_Microtubules_P0013	Chromatin_Microtubules_P0013_T00049.jpg	49	120	1191	363
Chromatin_Microtubules_P0013	Chromatin_Microtubules_P0013_T00050.jpg	50	120	1186	364
Chromatin_Microtubules_P0013	Chromatin_Microtubules_P0013_T00051.jpg	51	120	1187	360
Chromatin_Microtubules_P0013	Chromatin_Microtubules_P0013_T00052.jpg	52	120	1188	360
Chromatin_Microtubules_P0013	Chromatin_Microtubules_P0013_T00053.jpg	53	120	1189	364
Chromatin_Microtubules_P0013	Chromatin_Microtubules_P0013_T00054.jpg	54	120	1186	362

Fig. 36: *Trajectory Plot* – Input file. Example.

WIZARD TO GENERATE THE TRAJECTORY INFORMATION

ACC provides a wizard to generate the trajectory information (i.e. time-point ".csv" file) based on the image nomenclature. The following section explains how this works.

1) Open the *Regression Plane* and predict the plates of interest using the toolbar button "Predict Plates".
2) A "*PlateName*_singleCellRegressionPositions.csv" file for each predicted plate will be generated in the "RegResults" folder. Copy all the "*PlateName*_singleCellRegressionPositions.csv" files of the plates of interest into a single folder.
3) Now you are ready to generate the trajectory information requested by the *Trajectory Plot*. Click on the *Regression Plane* toolbar button "Plot Trajectories".
4) The computer will ask you to select the "*PlateName*_singleCellRegressionPositions.csv" of the plates of interest (you can select multiple files only if you copied them into the same folder, as described in point 2).
5) At this point, the computer will ask whether you already have the trajectory information file. If not, just click on: "Calculate now".

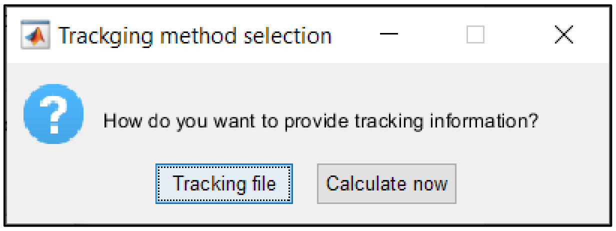

Fig. 37: Trajectory information generation

6) The wizard to generate the trajectory file appears. Now, the user must specify how the images are organized into time-lapse series by exploiting their names. Practically, he/she must define *separators* to split the image name, to tell the computer which part of the name indicates the fields of interest, and which part of the name indicates the time positions.

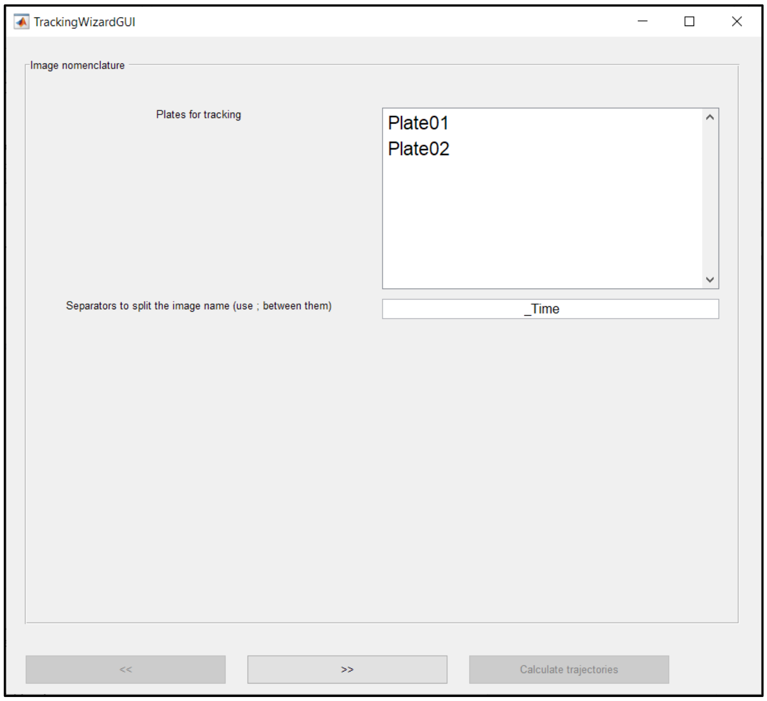

Fig. 38: Trajectory generator wizard: name analysis

For instance, if the names of the plates are:

```
Plate01
Plate02
```

and the names of the images are:

```
Field01_Time01.jpg
Field01_Time02.jpg
Field01_Time03.jpg
Field02_Time01.jpg
Field02_Time02.jpg
Field02_Time03.jpg
```

The user must define *"Time"* as a separator for the names of the images, and then select the option between those proposed, representing the field of interest, that is: "*1-____Time*" in this case to indicate the part: *"Field0*_"*. After that, he/she must select the numeric part of the image name indicating the time position, in this specific case: "*2-Time____*" to indicate the part: *"0*"*. At the end of this procedure, the computer will know how to connect the images in the different fields in the order of their time points.

7) A new window appears, allowing the user to define a few parameters for tracking the cells by exploiting a third-party tool (i.e. "track.pro", by John C. Crocker. License: Freeware).

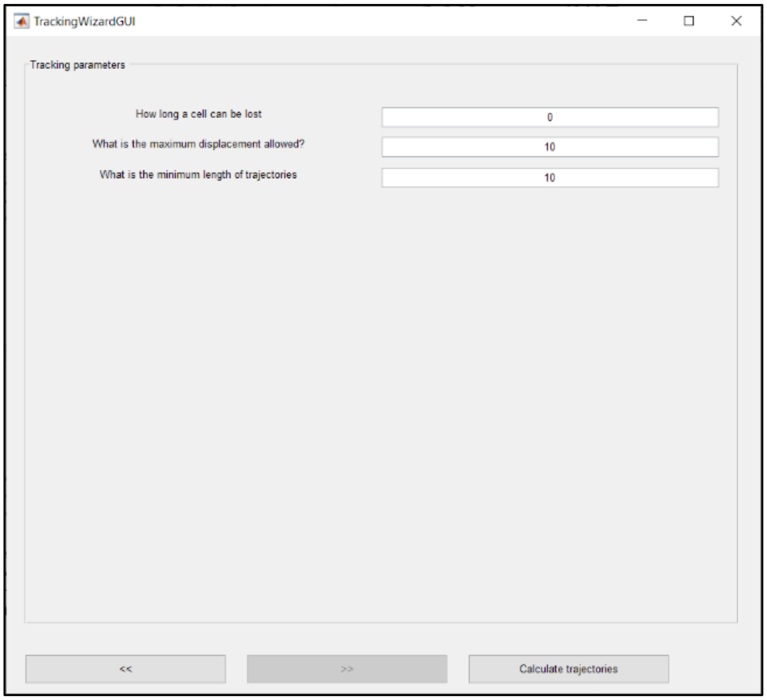

Fig. 39: Trajectory generator wizard: cell tracking

8) Finally, the computer asks where to save the "*Date*_Tracks.csv" file, and the *Trajectory Plot* GUI appears, with the trajectory file just generated automatically loaded.

9. ADDITIONAL INFORMATION

For additional information please, visit the ACC website and watch the video tutorials provided:

www.cellclassifier.org

And if you need our help, simply contact us!

Fig. 40: ACC logo.
