# Supplementary material for: Regression plane concept for analysing continuous cellular processes with machine learning
Source: Nat Commun. 2021 May 5;12:2532. doi: 10.1038/s41467-021-22866-x (PMC8100172; doi:10.1038/s41467-021-22866-x)
Supplement: Supplementary file 17 — Reporting Summary [file 41467_2021_22866_MOESM17_ESM.pdf]

nature research

Corresponding author(s): Peter Horvath

Last updated by author(s): 16/3/21

Reporting Summary

Nature Research wishes to improve the reproducibility of the work that we publish. This form provides structure for consistency and transparency in reporting. For further information on Nature Research policies, see our Editorial Policies and the Editorial Policy Checklist.

Statistics

For all statistical analyses, confirm that the following items are present in the figure legend, table legend, main text, or Methods section.

n/a	Confirmed	
☐	☒	The exact sample size (*n*) for each experimental group/condition, given as a discrete number and unit of measurement
☐	☒	A statement on whether measurements were taken from distinct samples or whether the same sample was measured repeatedly
☒	☐	The statistical test(s) used AND whether they are one- or two-sided<br>*Only common tests should be described solely by name; describe more complex techniques in the Methods section.*
☒	☐	A description of all covariates tested
☒	☐	A description of any assumptions or corrections, such as tests of normality and adjustment for multiple comparisons
☐	☒	A full description of the statistical parameters including central tendency (e.g. means) or other basic estimates (e.g. regression coefficient) AND variation (e.g. standard deviation) or associated estimates of uncertainty (e.g. confidence intervals)
☐	☒	For null hypothesis testing, the test statistic (e.g. *F*, *t*, *r*) with confidence intervals, effect sizes, degrees of freedom and *P* value noted<br>*Give P values as exact values whenever suitable.*
☒	☐	For Bayesian analysis, information on the choice of priors and Markov chain Monte Carlo settings
☒	☐	For hierarchical and complex designs, identification of the appropriate level for tests and full reporting of outcomes
☒	☐	Estimates of effect sizes (e.g. Cohen's *d*, Pearson's *r*), indicating how they were calculated

Our web collection on statistics for biologists contains articles on many of the points above.

Software and code

Policy information about availability of computer code

Data collection	The customized SIMCEP version is available as Supplementary Software 2.
Data analysis	CellProfiler v1 is available freely at: https://cellprofiler.org/previous-releases<br>The CIDRE framework is freely available at: https://github.com/smithk/cidre<br>The nucleAIzer pipeline source code is available at: https://github.com/spreka/biomagdsb<br>ACC's source code and standalone versions are available at: www.cellclassifier.org<br><br>The experiments involving Matlab were conducted with Matlab v9.5.0.1298439 (R2018b).<br>The data analysis involving ImageJ was conducted with version 1.49b.

For manuscripts utilizing custom algorithms or software that are central to the research but not yet described in published literature, software must be made available to editors and reviewers. We strongly encourage code deposition in a community repository (e.g. GitHub). See the Nature Research guidelines for submitting code & software for further information.

Data

Policy information about availability of data

All manuscripts must include a data availability statement. This statement should provide the following information, where applicable:

- Accession codes, unique identifiers, or web links for publicly available datasets
- A list of figures that have associated raw data
- A description of any restrictions on data availability

Synthetic dataset: https://data.broadinstitute.org/bbbc/image_sets.html (dataset ID: BBBC031).
Lipid droplet dataset: https://doi.org/10.6084/m9.figshare.c.5067638.v1.

Mitocheck dataset (public dataset previously described in [Cai et al. 2018]): http://www.mitocheck.org/mitotic_cell_atlas/downloads/v1.0.1/mitotic_cell_atlas_v1.0.1_fulldata.zip
The training set generated in this study is available as Supplementary Data 2.
Drosophila dataset: https://doi.org/10.6084/m9.figshare.c.5075093.v1.
Source data are provided with this paper for Fig. 1f, Suppl. Fig. 1c and 3d.

Field-specific reporting

Please select the one below that is the best fit for your research. If you are not sure, read the appropriate sections before making your selection.

☒ Life sciences ☐ Behavioural & social sciences ☐ Ecological, evolutionary & environmental sciences

For a reference copy of the document with all sections, see nature.com/documents/nr-reporting-summary-flat.pdf

Life sciences study design

All studies must disclose on these points even when the disclosure is negative.

Sample size	a) Synthetic dataset: Synthetic microscopy images were organized into a 24-well plate format, and the dataset was composed of 9 images/well, ~40 cells/image, for a total of 216 images and 8117 cells. The numbers chosen here are corresponding to a moderately-sized high-content screening experiment. The sample size in the plate was determined based on two considerations: 1) The plate size is not overwhelming so that users can identify the partitioning reasonably (possible plate sizes were 1,2,6,12,24,96,384) and 2) there are enough cells in each well (~9x40=360 cells/well) to separate the distributions clearly. In our study design at least half of the cell populations were separated by a distance of 0.1*sqrt(2)/2, which requires 52 samples/treatment with estimated variance of 0.01 to achieve 95% power and 5% false positive rate (Bernard Rosner: Fundamentals of Biostatistics, 8th edition 2016). However, to better resemble to a real high-throughput dataset we increased this number to 360/well. The number of testers (n = 10) were determined by the availability of recruited volunteers.<br><br>b) Lipid Droplet dataset: The cells were displaced into a 384-well plate and 9 images/well were acquired per channel for 2 identical plates yielding a total of 3,956 images of 232,084 cells (>2,200 cells per siRNA). The sample sizes correspond to the accepted standard in the field, similar numbers in high-throughput siRNA studies have been reported before for instance in Bartz et al. Cell Metabolism (2017) - https://www.sciencedirect.com/science/article/pii/S1550413109001570#sec4 and Chapuis et al. Acta Neuropathologica (2017) - https://link.springer.com/article/10.1007/s00401-016-1652-z#Sec2<br><br>c) MitoCheck dataset [Cai et al. 2018]: The downloaded dataset included images of 29 endogenously tagged proteins distributed to 92 folders. The cell number ranged from 1 to 11 in the folders, resulting altogether in 498 analyzed cell. Each cell was imaged over 40 frames, hence the total number of images in the experiment was 19920. Our sample number was determined by the original data in this case.<br><br>d) Blood cell differentiation dataset: Blood samples of 10 larvae were collected. We acquired 15-frame image sequences/field (141 fields) on 3 channels: brightfield, mCherry, and EGFP yielding a total of 4,230 images (2 plates, 2,115 images in each). The sample sizes in this experiment correspond to the accepted standard in the field (in comparison, in the mitosis study from above at most 55 cells/condition were examined in our case this number ranged between and 900-2500)<br><br>e) Active learning experiments: We repeated each experiment 50 times to assess the performance of the Active Learning strategies. The sample size matches with the current standard in the field, similar repetition numbers, initial training set sizes and active queries have been reported in the field of active learning in Yang et al. Pattern Recognition (2018) - https://www.sciencedirect.com/science/article/abs/pii/S0031320318302140, Settles et al. Proc. of EMNLP (2008) - https://www.aclweb.org/anthology/D08-1112.pdf and Schein et al. Machine Learning (2007) - https://link.springer.com/content/pdf/10.1007/s10994-007-5019-5.pdf
Data exclusions	a) Synthetic dataset: none.<br><br>b) Lipid Droplet dataset: we acquired 3 multi-well plates but we only used and shared 2 due to batch effects verified on the one removed.<br><br>c) MitoCheck dataset [Cai et al. 2018]: removed 7 cells due to segmentation errors.<br><br>d) Blood cell differentiation dataset: none.<br><br>e) Active learning experiments: None.
Replication	a) Synthetic dataset: The experiments were conducted by 10 independent users. All attempts at replication were successful.<br><br>b) Lipid Droplet dataset: replicated on 2 multi-well plates, each siRNA treatment had 3-9 replicates in different wells each plate. Both attempts at replication were successful.<br><br>c) MitoCheck dataset [Cai et al. 2018]: Replication corresponding to the original paper. The dataset included 2-5 replicates for each fluroescent tagging. All attempts at replication were successful.<br><br>d) Blood cell differentiation dataset: We used 10 larvae as biological replicates. The experiments were replicated successfully 3 times.<br><br>e) Active learning experiments: repeatability was ensured by using fixed pseudo-random generator seed values. The experiment was replicated two times yielding the same result.
Randomization	a) Synthetic dataset: Users were randomly put to different groups (regression vs classification).

Randomization	b) Lipid Droplet dataset: The siRNA treatments to the wells were allocated randomly.<br><br>c) MitoCheck dataset [Cai et al. 2018]: not applicable, we did not have to take any biased decisions in performing the experiment.<br><br>d) Blood cell differentiation dataset: not applicable, we did not have to take any biased decisions in performing the experiment.<br><br>e) Active learning experiments: The initial test sets were randomized.
Blinding	a) Synthetic dataset: The testers were blinded from any information regarding the ground truth of the experiment.<br><br>b) Lipid Droplet dataset: not applicable, there was no ground-truth information available on this data beforehand.<br><br>c) MitoCheck dataset [Cai et al. 2018]: not applicable, there was no ground-truth information to be hidden from the annotator.<br><br>d) Blood cell differentiation dataset: not applicable, there was no ground-truth information available on this data beforehand.<br><br>e) Active learning experiments: not applicable, there were no users involved in this study (naturally the algorithm did not have access to the ground truth information).

Reporting for specific materials, systems and methods

We require information from authors about some types of materials, experimental systems and methods used in many studies. Here, indicate whether each material, system or method listed is relevant to your study. If you are not sure if a list item applies to your research, read the appropriate section before selecting a response.

Materials & experimental systems

n/a	Involved in the study
☒	☐ Antibodies
☐	☒ Eukaryotic cell lines
☒	☐ Palaeontology and archaeology
☐	☒ Animals and other organisms
☐	☒ Human research participants
☒	☐ Clinical data
☒	☐ Dual use research of concern

Methods

n/a	Involved in the study
☒	☐ ChIP-seq
☒	☐ Flow cytometry
☒	☐ MRI-based neuroimaging

Eukaryotic cell lines

Policy information about cell lines

Cell line source(s)	The Huh7 hepatocellular carcinoma cell line (Nakabayashi et al., 1982; from Prof. Ilkka Julkunen, THL, Finland) is commercially available through different vendors (for instance https://cellbank.nibiohn.go.jp/~cellbank/en/search_res_det.cgi?ID=385).
Authentication	The cell line was authenticated using Promega StemEliteTM ID System at Genomics Unit of Technology Centre, Institute for Molecular Medicine Finland (FIMM), University of Helsinki. According to the manufacturer, the authentication method is based on DNA fingerprinting by short tandem repeat (STR) analysis, allowing co-amplification and three-color detection of ten human loci.
Mycoplasma contamination	The Huh7 cell line tested negative for mycoplasma contamination.
Commonly misidentified lines (See ICLAC register)	No commonly misidentified lines were involved in this study.

Animals and other organisms

Policy information about studies involving animals; ARRIVE guidelines recommended for reporting animal research

Laboratory animals	Species: Drosophila melanogaster. Sex: undefined. Age: 3rd instar larva. Strain: eaterGFP as a marker of plasmatocytes, MSNF9MOmCherry as a marker of lamellocytes.
Wild animals	No wild animals were captured or observed in this study.
Field-collected samples	No field-collected samples were involved in this study.
Ethics oversight	The work with genetically modified drosophila melanogaster does not require ethical permit, however the creation of GMO requires a permit. The Biological Research Centre in Szeged, Hungary was authorized for working and creating GMO by the Ministry of Rural Development (Hungary) on 20th January 2014 with document registration number SF/66/2014.

Note that full information on the approval of the study protocol must also be provided in the manuscript.

Human research participants

Policy information about studies involving human research participants

Population characteristics	Our user-base consisted of microscopy experts with varying experience in the field. The median age of users was 29.5.
Recruitment	We recruited the users from our network of collaborators to form a heterogeneous population of testers.
Ethics oversight	Our study design did not require ethical review.

Note that full information on the approval of the study protocol must also be provided in the manuscript.
